# Global current systems in the magnetosphere of Mercury

**DOI:** 10.1038/s41467-026-74847-7

**Published:** 2026-06-23

**Authors:** Rentong Lin, Shiyong Huang, Zhigang Yuan, James A. Slavin, Fouad Sahraoui, Jim M. Raines, Kui Jiang, Lihui Chai, Honghong Wu, Yue Dong, Brian J. Anderson, Yunyun Wei, Sibo Xu, Qiyang Xiong, Jian Zhang, Zhao Wang, Lin Yu

**Affiliations:** 1 School of Earth and Space Science and Technology, Wuhan University, Wuhan, China; 2 Department of Climate and Space Sciences and Engineering, University of Michigan, Ann Arbor, MI USA; 3 Laboratoire de Physique des Plasmas, CNRS-Ecole Polytechnique-Sorbonne Université-Paris-Saclay-Observatoire de Paris-Meudon, Palaiseau, France; 4 Key Laboratory of Earth and Planetary Physics, Institute of Geology and Geophysics, Chinese Academy of Sciences, Beijing, China; 5 The Johns Hopkins University Applied Physics Laboratory, Laurel, MD USA

**Keywords:** Magnetospheric physics, Inner planets

## Abstract

The global current systems of a planet represent electrodynamic interactions between its different parts, and between its magnetosphere and the ambient solar wind. Although some local currents of Mercury’s magnetosphere have already been revealed by previous observational studies, the global picture of current systems in Mercury’s magnetosphere remains unknown. Here we reconstruct the global current systems in the Mercury’s magnetosphere, using five years magnetic field and plasma measurements made by the MErcury Surface, Space ENvironment, GEochemistry, and Ranging (MESSENGER) spacecraft. It reveals a complex picture: a magnetopause current, cross-tail current, inner and outer equatorial ring currents and an unexpected polar ring current. The equatorial ring currents and the polar ring current agree with the prediction of the plasma drifting model in a region with a pressure gradient. These ring currents could reshape Mercury’s magnetosphere and reveal new dynamics and energy transfer processes in Mercury.

## Abstract (web summary)

Mercury’s magnetic environment is shaped by electric currents driven by the solar wind. Here, the authors show a global map of these currents, revealing equatorial ring currents and a previously unknown current around Mercury’s north pole.

## Introduction

Mercury has an intrinsic dipole magnetic field characterized by a northward offset^1–3^. Its interaction with the solar wind forms a magnetosphere, first discovered by Mariner 10^4^ and subsequently studied by NASA’s MErcury Surface, Space ENvironment, GEochemistry, and Ranging (MESSENGER^5^;) and the ESA-JAXA BepiColombo missions^6^. Due to the planet’s proximity to the Sun, Mercury’s magnetosphere is highly compressed on the dayside by the dense solar wind.

Electric currents play an important role in the deformation of the magnetosphere as an agent that couples different plasma regimes and mediates stresses^7^. For this reason, identifying the major current systems is essential for understanding how Mercury’s magnetosphere is shaped and how it evolves. Mercury is used to be regarded as a mini-size Earth, thus, current systems in its magnetosphere were regarded as the same as those in Earth’s magnetosphere until NASA’s MESSENGER orbiter distinguished unique features in Mercury’s magnetosphere. Mercury’s small magnetospheric size, strong solar wind forcing, conducting planetary interior, and tenuous exosphere all differs from those of Earth, which can all affect the flow pattern and the closure of magnetospheric currents.

Over the past decade, several types of magnetospheric currents have been reported at Mercury^7–13^. Anderson et al. ^12^ found evidence for steady-state field-aligned (Birkeland currents) over Mercury’s northern polar region based on magnetic field data from MESSENGER. These currents were inferred to flow downward on the dawn side and upward on the dusk side, and were proposed to close through the planetary interior based on a spherical-shell conductance model. And hybrid simulation results showed that Region 2-like currents can form below the perigee of MESSENGER when the sodium exosphere is sufficiently dense^14^.

Evidence of equatorial currents was also found in Mercury’s magnetosphere. Zhao et al. ^9^ presented the evidence of westward ring current using particle measurements from MESSENGER, whereas Shi et al. ^10^ proposed an eastward current in Mercury’s magnetotail instead using magnetic field measurements. While the westward ring current has been validated by test-particle simulations^9^, while the eastward current is well supported by hybrid simulations^10^ and by the empirical magnetospheric model of Pump et al. ^15^.

Transient current systems have also been identified. In particular, substorm current wedge (SCW) currents^13,16^ are expected to occur at low to middle latitudes on the night side during storm time^17,18^. Poh et al. ^19^ reported the formation of SCW in Mercury, which couples Mercury to its nightside magnetosphere.

Previous studies focused on local magnetospheric currents and characterized specific current systems, which significantly advanced our understanding of Mercury’s magnetospheric currents. However, there existed discrepancies or contradictions across studies and comprehensive observational studies that can establish or deny that Mercury’s global currents are, to date, still lacking.

The present study fills this gap by presenting a global map of current systems in Mercury’s magnetosphere based on the MESSENGER data. In particular, this study reveals the existence of a polar ring current and unravels the plasma species carrying the magnetospheric currents, which brings new perspectives in considering the dynamics of Mercury’s magnetosphere.

## Results

We obtain a three-dimensional (3D) map of the magnetic field in Mercury’s magnetosphere (Supplementary Fig. 1) by time averaging of 5 years (2011–2015) of magnetic field measurements by MESSENGER. This approach is intended to recover the large-scale quasi-steady magnetic configuration and associated current systems, rather than short-time-scale transient structures observed along individual spacecraft passes. Current density is then derived by applying the first-order central finite difference method to this averaged field at the spatial resolution of 0.15 *R*_M_, where *R*_M_ is Mercury’s Radius, about 2440 km. We obtain a three-dimensional map of current density in Mercury’s magnetosphere in the Mercury Solar Orbital (MSO) coordinates, where the +x points sunward, +z points to the north pole and +y completes the right-handed coordinate system (Supplementary Fig. 2). Details of the MESSENGER data, the first-order central finite difference method and the error analysis are provided in the “Methods” section.

We transform the current density from the Cartesian coordinates into cylindrical coordinates, which makes the different current systems easier to identify from their patterns. In this cylindrical coordinate system, the azimuthal direction is defined around the +Z axis following the right-hand rule, with the +X direction taken as zero of *θ*, where *θ* is the azimuthal angle.

**Fig. 1:**
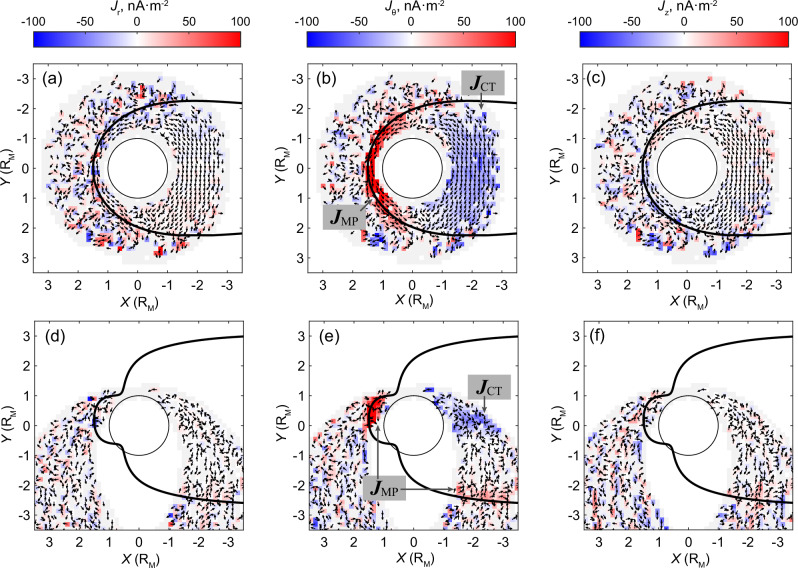
Slices of the Mercury’s current systems in the X-Y and X-Z planes. **a**–**c** Show the electric current density in equatorial plane within |*z*| ≤ 0.15 *R*_M_. **d–f** Show the current in the day–night plane within |*y*| ≤ 0.15 *R*_M_. *R*_M_ is the Mercury’s radius, about 2440 km. *J*_MP_ is the magnetopause current, and *J*_CT_ is the cross-tail current. Each column of panels is colored by the components of the current density in cylindrical coordinates, respectively. The three mutually orthogonal current density components in the cylindrical coordinate system are the radial component *J*_r_ which is directed outward perpendicular to the central longitudinal z-axis, the azimuthal component *J*_*θ*_ which is directed tangentially around the *z*-axis in the direction of increasing azimuthal angle, and the axial component *J*_z_, which is directed parallel to the central *z*-axis. Arrows show the current direction in the projected plane of each panel. The thin black circle in each panel represents Mercury. The bolded black curves represent the magnetopause^23^. The grid cells in gray represent no valid measurements. Source data are provided as a Source data file.

Figure 1 presents the current density components in cylindrical coordinates in X–Y plane of the equator and in X–Z plane of the meridian. At the dayside magnetopause, strong consecutive current, over 100 nA m^−2^, flows eastward (the azimuthal component of current density *J*_*θ*_ > 0 in Fig. 1b) from the dawn to the dusk along the magnetopause. At the flank of the magnetotail (−2.5 *R*_M_ < *z* < −1.5 *R*_M_ and *x* < −1 *R*_M_ in Fig. 1e), the current flows eastward within *z* < 0 (*J*_*θ*_ > 0). The current at the nightside flank magnetopause is less than 50 nA m^−2^, smaller than that at dayside magnetopause. This current can be clarified as the magnetopause current (*J*_MP_).

Owing to the orbital geometry of the MESSENGER, current at the dayside magnetopause within *z* < 0 and current at the nightside magnetopause within *z* > 0 cannot be estimated because the corresponding regions were not well sampled.

**Fig. 2:**
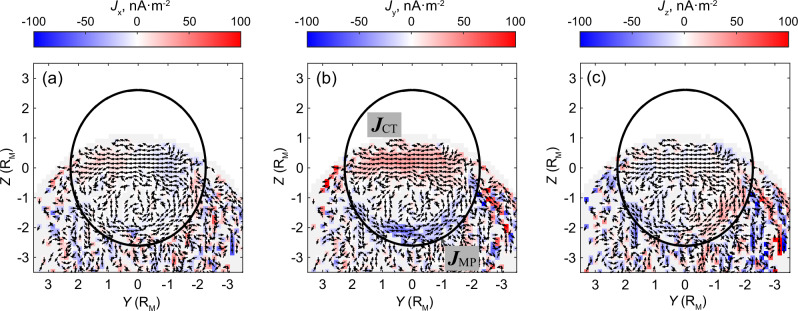
Slices of the Mercury’s current systems in the Y-Z plane within −1.80 *R*_M_ ≤ *x* ≤ −1.50 *R*_M_. **a** Current density component *J*_x_. **b** Current density component *J*_y_. **c** Current density component *J*_z_. *R*_M_ is the Mercury’s Radius, about 2440 km. Each column of panels is colored by the components of the current density in Mercury Solar Orbital (MSO) coordinates, respectively. *J*_MP_ is the magnetopause current and ***J***_CT_ is the cross-tail current. Arrows show the current direction in the projected plane of each panel. The bolded black curves represent the magnetopause. The grid cells in gray represent no valid measurements. Source data are provided as a Source data file.

Figure 2 presents the current density components in MSO coordinates in Y-Z plane through the magnetotail. In the magnetotail, the current flows westward from the dawn to the dusk (*J*_y_ > 0 within |*y*| < 2 *R*_M_ and |*z*| < 1 *R*_M_ in Fig. 2b), which can be also captured in X-Y plane (*J*_*θ*_ < 0 within *x* < 0 and |*y*| < 2 *R*_M_ in Fig. 1b and within *x* < 0 and |*z*| < 0.5 R_M_ in Fig. 1e). This current in the Mercury’s magnetotail is the same as the cross-tail current (*J*_CT_) in the Earth’s magnetotail^20–22^. The cross-tail current closes via the nightside magnetopause current at the flank of the magnetotail (Figs. 1e and 2b). The bolded black curves represent the magnetopause^23^.

In addition to the magnetopause current and the cross-tail current, our reconstruction reveals other current systems in Mercury’s inner magnetosphere.

**Fig. 3:**
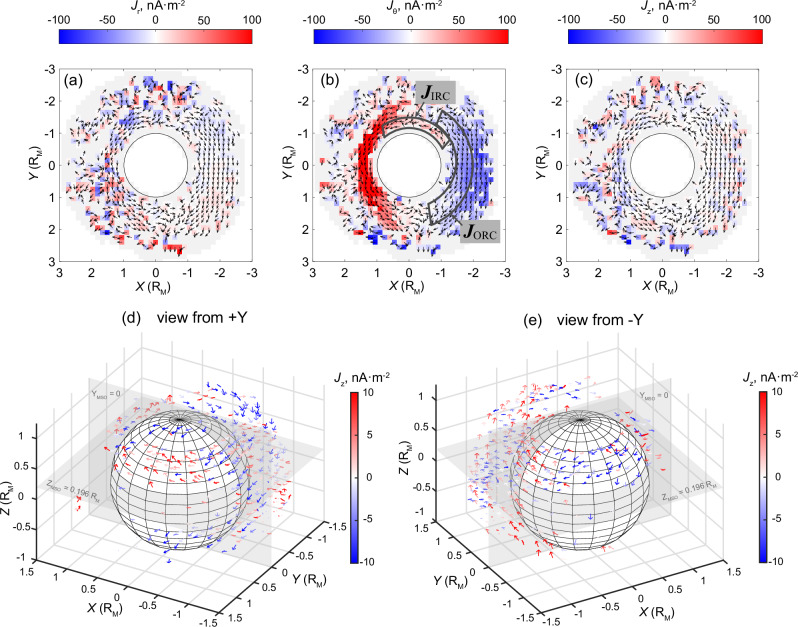
Slices in the X-Y plane and side view of the three-dimensional map of Mercury’s current systems. **a**–**c** show a slice in the X-Y plane within 0.15 *R*_M_ ≤ *z* ≤ 0.30 *R*_M_. In **d**,** e**, the axis range is limited to |*x*| < 1.5 *R*_M_, |*y*| < 1.5 *R*_M_ and |*z*| < 1.25 *R*_M_. *R*_M_ is Mercury’s Radius, about 2440 km. Each column of panels is colored by the components of the current density in cylindrical coordinates, respectively. *J*_IRC_ is the inner ring current and *J*_ORC_ is the outer ring current. Arrows in (**a**–**c**) show the current direction in the projected plane of each panel. The black circles in (**a**–**c**) and the sphere in (**d**, **e**) represent Mercury. The grid cells in gray represent no valid measurements. The regions used to identify *J*_ORC_ and *J*_IRC_ are indicated by the gray enclosed areas in (**b**). The magnetic equatorial plane for reference in (**d**,** e**) is indicated by the translucent surface (*z* = 0.196 *R*_M_). Arrows represent the current density vector. To present the *J*_ORC_ distinctly, arrows with *J*_*θ*_ > 0 are not shown. Color of arrows in (**d**–**f**) represents the z-component of the current density in cylindrical coordinates. Source data are provided as a Source data file.

Figure 3 displays the current density components in the X-Y plane within 0.15 *R*_M_ ≤ *z* ≤ 0.30 *R*_M_ and in the 3D view. As shown in Fig. 3b, there exists a clockwise current in the near-Mercury magnetotail (−1.4 *R*_M_ < *x* < 0). In the 3D map of Mercury’s current systems (Fig. 3d, e), the clockwise current flows at higher altitude as it comes to the dayside. The axial component of current density *J*_z_ is positive within 0 <*y* < 1 *R*_M_ and |*x*| < 1 *R*_M_ whereas *J*_z_ become negative within −1 *R*_M_ < *y* < 0 and |*x*| < 1 *R*_M_ (Fig. 3d, e), which suggests that the current flows upward within 0 <*y* < 1 *R*_M_ and |*x*| < 1 *R*_M_ and flows downward within −1 *R*_M_ < *y* < 0 and |*x*| < 1 *R*_M_.

This flow pattern suggests that the clockwise current does not flow in the equatorial plane but flows upward at dusk-side (*y* > 0), peaks at noon, comes down at dawn-side (*y* < 0) and ends at midnight, completing an oval. This clockwise current is named the outer equatorial ring current (*J*_ORC_), although it does not only flow in the equatorial plane. Current density of *J*_ORC_ is less than 30 nA m^−2^ in the nightside, and approximately 100 nA m^−2^ in the dayside.

A second azimuthal current is also observed. This current flows counter-clockwise and is located mainly on the dawn side (marked by the gray enclosed regions in Fig. 3b). We refer to this current as the inner equatorial ring current, *J*_IRC_. It becomes clear near 03:00 LT, connects with the dayside magnetopause current near 09:00 LT, separates from the magnetopause current near 15:00 LT, and weakens toward 20:00 LT. Its direction is opposite to that of *J*_ORC_.

The closure of *J*_IRC_ near midnight remains uncertain because MESSENGER did not sample sufficiently low altitudes in this region. Near noon, *J*_IRC_ is also difficult to separate from the magnetopause current because both currents flow in the counter-clockwise direction and no clear boundary is visible between them.

We therefore treat *J*_IRC_ as a less well-constrained current system than *J*_MP_, *J*_CT_, and *J*_ORC_. This uncertainty arises partly from the finite-difference method, which cannot estimate current densities at the edges of the sampled grid, and partly from the removal of grid cells with relative errors greater than 0.5 (details provided in the “Methods” section). As a result, only a small number of valid grid cells remain inside ρ < 1.35 *R*_M_, where ρ is defined as $\sqrt{{x}^{2}+{y}^{2}}$, and about 43% of these points show features consistent with *J*_IRC_. Nevertheless, an eastward current in this region has also been reproduced by hybrid simulations^10^. Given the limited statistics and the high variability of Mercury’s magnetosphere, further in situ measurements are needed to confirm this current. Future observations by the magnetometers^24,25^ and plasma instruments^26^ on BepiColombo^27^ will be particularly important for this purpose.

**Fig. 4:**
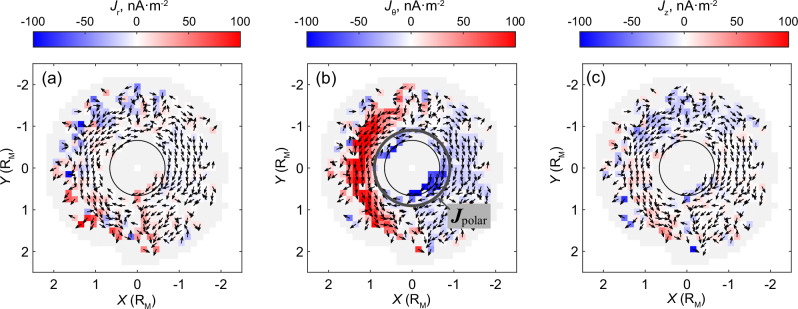
Slices of Mercury’s current systems in the X-Y plane within 0.75 *R*_M_ ≤ *z* ≤ 1.20 *R*_M_. **a** Current density component *J*_r_. **b** Current density component *J*_θ_. **c** Current density component *J*_z_. *R*_M_ is Mercury’s radius, about 2440 km. Each column of panels is colored by the components of the current density in cylindrical coordinates. *J*_polar_ is the polar ring current. Arrows show the current direction in the projected plane of each panel. The black circle in each panel represents Mercury. The grid cells in gray represent no valid measurements. The region identified as *J*_polar_ is indicated by the gray circle in (**b**). Source data are provided as a Source data file.

We also identify a persistent high-latitude azimuthal current encircling clockwise (opposite to *J*_MP_) around the north pole, which is named as polar ring current (*J*_polar_; Fig. 4b). The current density of the *J*_polar_ is approximately 50–100 nA m^−2^.

The current density within 0.45 *R*_M_ ≤ *z* ≤ 0.60 *R*_M_ (Supplementary Fig. 3b) is smaller than those at equator and in polar regions (Supplementary Fig. 3a, c), which indicates that the *J*_ORC_ and the *J*_polar_ are separated. The confinement of the *J*_polar_ to northern high latitudes and its separation from the equatorial current system are further illustrated by slices at different *z* intervals (Supplementary Fig. 4). For the high-latitude current, we use a thicker z-slice than in the equatorial analysis because the polar region contains fewer valid grid cells after quality control. A sensitivity test using a less stringent quality-control criterion yields a more continuous annular morphology of the high-latitude current (Supplementary Fig. 5). Additional comparisons using both 0.15 *R*_M_ and 0.45 *R*_M_ slices under different error-filtering criteria are shown in Supplementary Fig. 6. These tests demonstrate that the continuity of the annular pattern is sensitive to data coverage and filtering choice, but the dominant high-latitude azimuthal current persists across these alternative reconstructions.

This ring current flows primarily in the X-Y plane at high latitudes (Fig. 4b, c). There is a gap of current density between the equator and the high latitude region on the nightside, suggesting that this ring current encircling the north pole is different from the *J*_ORC_.

It should be noted that this region includes open field lines connecting tailward. Considering Ohm’s law, the current is highly associated with the plasma motions if the conductivity is finite. However, the ion and electron motions in Mercury’s magnetosphere are not fully determined so far. Thus, it is not clear how the ion and electron motions affect the current *J*_polar_. Although the moving open field lines may affect the current *J*_polar_, its evaluation is beyond the scope of this work.

The spacecraft visited the same magnetospheric regions under certain solar wind conditions which mainly scale with heliocentric distance between Mercury and Sun. To assess the effects of changing solar-wind forcing and orbital sampling bias, we classified the data into two categories based on heliocentric distance *D* according to *D* ≤ 0.387 au and *D* > 0.387 au, where the minimum heliocentric distance *D*_min_ is about 0.307 au, the maximum heliocentric distance *D*_max_ is about 0.467 au, and the average value of these two distances is about 0.387 au (au is about 1.496 × 10^8 ^km). The current densities are calculated separately by these two datasets (Supplementary Figs. 7–10). When Mercury is closer to the Sun, the magnetopause current and cross-tail current are stronger and the magnetopause is located closer to the planet, consistent with a more compressed magnetosphere. The inner equatorial ring current is more clearly resolved at larger heliocentric distance, whereas the high-latitude azimuthal current associated with *J*_polar_ remains detectable in both subsets. These sensitivity tests support the robustness of the principal large-scale current systems inferred here.

At low-middle latitudes on the night side is the expected site for SCW currents^13,16^. The substorm SCW currents on the night side include the dusk-to-dawn current at equator, the dawn-to-dusk current at low latitude and field-aligned currents (FACs) in between^19^. Although the dawn-to-dusk current at high latitude, i.e. *J*_polar_, is observed, we do not find either the dusk-to-dawn current at equator or the FACs on the night side. The reason may be that the number of substorm events in the data set is not large enough for the SCW magnetic field perturbations to rise above the other current signatures.

Birkeland currents (also named as field-aligned currents, FACs) at Mercury were first reported from Mariner 10 observations^13^. The signatures of steady-state FACs in the polar regions were revealed after removing the average magnetospheric signal from empirical magnetosphere models (12, 15; different from the methodology used in the present study). These FACs of density 10–40 nA m^−2^ flow downward at dawnside and upward at duskside in the polar region. There should exist a closed circuit between FACs. However, Mercury cannot sustain an atmosphere but owns only a thin sodium exo-ionosphere from planetary sodium group ions^26^, in which Pederson currents may be formed as the current closure path^14^. Another possibility is closure via Ohmic currents through the low-conductivity crust and upper mantle^12,28^. Thus, the closure of FACs is still debated.

## Discussion

One should note that the bifurcated neutral current sheet^29^ is a critical component of Mercury’s nightside current systems, closely linked to the cross-tail current identified herein. The westward *J*_CT_ in the nightside magnetotail closes through the nightside magnetopause current, with its main region overlapping spatially with the bifurcated sheet’s distribution. Moreover, the current carriers in the bifurcated sheets are mainly solar wind protons, which also drives the cross-tail current. Thus, the bifurcated neutral current sheet is a primary manifestation of the nightside current system in Mercury’s magnetosphere, contributing significantly to current density and shaping the magnetotail’s electrodynamic structure.

The physical nature of the *J*_MP_ and *J*_CT_ has been well discussed^27^. Both currents are fundamentally diamagnetic currents, but they occur in different regions: the magnetopause current balances external solar-wind pressure against the planetary field, whereas the cross-tail current balances plasma-sheet pressure against the stretched tail magnetic field. Here, we focus on the physical nature of *J*_ORC_, *J*_IRC_ and *J*_polar_.

**Fig. 5:**
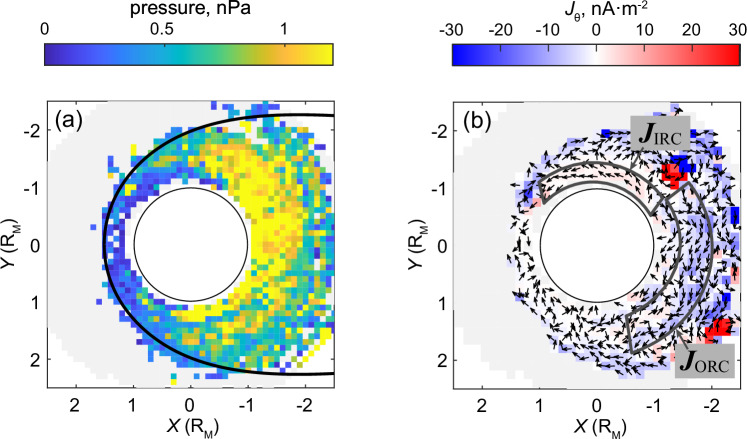
Distribution of plasma pressure of H^+^ ions and the current density in the X-Y plane centered at *z* = 0.20 R_M_. The current density is estimated by $-\frac{\nabla P\times {{{\bf{B}}}}}{{|{{{\bf{B}}}}|}^{2}}$ based on plasma and magnetic field measurements. **a** Plasma pressure of protons. **b** Current density component *J*_θ_. *R*_M_ is Mercury’s radius, about 2440 km. The plasma pressure is derived from the Fast Image Plasma Spectrometer (FIPS) measurements^31^. *J*_IRC_ is the inner ring current and *J*_ORC_ is the outer ring current. The current density is estimated based on magnetic field and plasma measurements by the MESSENGER orbiter. **b** is colored by the azimuthal component in the cylindrical coordinates. Arrows show the current direction in the projected plane. The thin black circles represent Mercury. The grid cells in gray represent no valid measurements. The regions corresponding to *J*_IRC_ and *J*_ORC_ are indicated for comparison with the ring-current morphology identified from magnetic-field-based current reconstruction. Source data are provided as a Source data file.

Ring currents generally result from magnetization of charged particles. Part of the magnetization current cancels the component of the current due to guiding-center drift of charged particles. The remaining magnetization current, which is proportional to the plasma pressure gradient, presents the circular current. For static conditions and isotropic plasma pressure, the current density perpendicular to the magnetic field **J**_⊥_ can be written as

1 $${{{{\bf{J}}}}}_{{{{\boldsymbol{\perp }}}}}=-\frac{\nabla P\times {{{\bf{B}}}}}{{\left|{{{\bf{B}}}}\right|}^{2}}$$

where *P* is plasma pressure, **B** is magnetic field vector. This expression shows that a pressure gradient perpendicular to the magnetic field can generate an azimuthal diamagnetic current. Protons in the regions of *J*_ORC_ and *J*_IRC_ are both stably trapped (pitch angle about 90°; Supplementary Fig. 11a, b). It indicates that protons may be the current carrier. Note that stationary transverse currents in geospace are generated by plasma pressure gradients^30^. Based on the average distribution of pressure of protons (Fig. 5a), there exists a plasma pressure gradient in the Mercury magnetotail. The perpendicular current density estimated by plasma pressure gradient is shown in Fig. 5b. The direction of the *J*_ORC_ and *J*_IRC_ can be identified, whereas the values of current density differ from those estimated by magnetic field measurements. The discrepancy could be attributed to the limited number of effective plasma sampling channels and limited temporal resolution of the Fast Image Plasma Spectrometer (FIPS) onboard MESSENGER, which leads to an underestimation of the plasma pressure. If there is a plasma pressure maximum, the current directions on the two sides of the maximum should be reversed. On the away-from-Mercury side of the plasma pressure peak, the direction of remaining magnetization current consistent with the plasma pressure gradient should be westward, i.e., *J*_ORC_. On the close-to-Mercury side of the plasma pressure peak, the direction of remaining magnetization current consistent with the plasma pressure gradient should be eastward, i.e., *J*_IRC_. At midnight, the inferred pressure maximum is located at a radial distance of approximately 1.15–1.25 *R*_M_, broadly comparable to the inner edge of the current sheet reported by ref. ^19^. However, since MESSENGER did not sample lower altitudes in this region, the true pressure maximum may lie somewhat closer to Mercury. Thus, it is hard to see the evidence of *J*_IRC_ at midnight, but it is much easier for parts of *J*_ORC_ and *J*_IRC_ to be seen at dawn and dusk. Note that features of the *J*_ORC_ and the *J*_IRC_ are consistent with those of the westward ring current proposed by Zhao et al. ^9^ and those of the eastward current proposed by Shi et al. ^10^, respectively. Thus, the two apparently different current directions reported in previous studies may represent different parts of a pressure-gradient-driven ring-current system in Mercury’s magnetotail.

On the dayside, since the dipole magnetic field of Mercury is much weaker than that of Earth, and the solar wind pressure at Mercury is much stronger, the dayside magnetosphere is highly compressed and the position of the magnetopause is much closer to Mercury’s surface^32,33^. The highly compressed magnetosphere results in a local minimum of the magnetic field in between the magnetopause at subsolar and the cusp at dayside, which guides particles to off-equatorial latitudes, i.e., Shabansky orbit^9,34–36^. This displacement causes the *J*_ORC_ on the dayside to split into two distinct branches, i.e., cut ring current^37–40^. However, it should be mentioned that the current *J*_ORC_ could possibly be the combination of two partial ring currents on dayside and nightside. More observations from the upcoming BepiColombo missions are needed before ruling out this possibility.

**Fig. 6:**
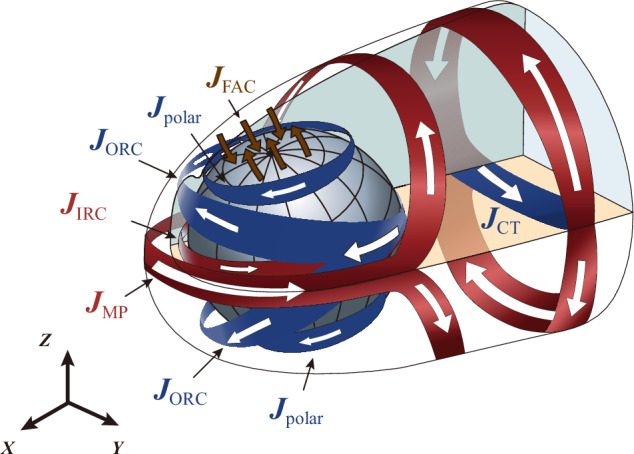
Illustration of the global current systems in Mercury’s magnetosphere. This figure shows different currents in the Mercury’s magnetosphere, i.e., the magnetopause current *J*_MP_, the cross-tail current *J*_CT_, the outer ring current *J*_ORC_, the inner ring current *J*_IRC_, the field-aligned current *J*_FAC_, and the polar ring current *J*_polar_. The red arrows represent currents in counterclockwise overlooked from the north pole. The blue arrows represent currents in clockwise overlooked from the north pole. The brown arrows represent field-aligned currents^12,15^. The light yellow area represents the equatorial plane and the light blue area represents the slice of the magnetosphere.

The four types of currents (i.e., *J*_MP_, *J*_CT_, *J*_ORC_, and *J*_IRC_) are, to some extent, foreseeable. However, the polar ring current *J*_polar_ is unexpected and has not been observed in the Earth’s magnetosphere or other magnetospheres of the solar system with intrinsic dipole magnetic fields. All these currents in Mercury’s magnetosphere are illustrated in Fig. 6.

The formation and physical nature of *J*_polar_ could be the same as other ring currents, i.e., opposite drift of ions and electrons in the region with plasma pressure peak ^40,41^. Protons in the regions of *J*_polar_ are stably trapped (Supplementary Fig. 11c), which indicates that protons are candidate of the current carrier. It should be noted that there exists a plasma pressure gradient from higher latitudes toward the cusp region (Supplementary Fig. 12^42,43^). The magnetic field near the north pole is predominantly z-directed, meaning that the magnetic field vector near the north pole points almost perpendicularly to the planetary surface. This results in a clockwise current around the north pole on the dayside. On the nightside, there exists a pressure gradient pointing from high latitude to low latitude. The local magnetic field vector roughly points towards the Mercury surface. The direction of estimated local current on the nightside is also clockwise viewed from the north pole.

An approximate estimation can be made by setting a characteristic length *L* for which ∇*p* ∼ *p*/*L* satisfies, where *p* is plasma pressure. In the cusp region, the thermal pressure of hydrogen ions is about 1 nPa (Supplementary Fig. 12). Consider *J*_polar_ ≈ 20 nA m^−2^ and |**B**| ranging from 200 to 400 nT, the characteristic length *L* can be approximated by *L* ~ $\frac{p}{\left|{{{{\bf{J}}}}}_{\perp }\right|\left|{{{\bf{B}}}}\right|}$ ~ 125–250 km, i.e., 0.05–0.10 *R*_M_., where $\left|{{{{\bf{J}}}}}_{\perp }\right|$ is the current density perpendicular to the magnetic field. If adopting the density and temperature of H^+^ ions from ref. ^42^ and from Sun et al. ^43^, then the estimated characteristic length *L* is approximately 214–428 km, i.e., 0.09–0.18 *R*_M_, and 280–560 km, i.e., 0.12–0.23 *R*_M_, respectively. The estimated characteristic scale is comparable to that of *J*_polar_ illustrated in Fig. 4. This indicates that protons could act as the carrier of the *J*_polar_ and that the polar ring current is contributed by the diamagnetic current. However, it is difficult to make a more detailed quantitative estimation so far. More specific and accurate quantitative estimation can be made with the orbital data from the Bepicolombo mission in the near future.

The characteristics of *J*_polar_ can also be found in previous simulation results of the 3-D AMRVAC and Yagi MHD models^44^ but missed in other simulation models (e.g.^45,46^). The *J*_y_ component of the current density of AMRVAC model is less than 0 (i.e., negative) in the dayside polar regions and greater than 0 (i.e., positive) in the nightside polar regions. This is consistent with the characteristics of *J*_polar_ presented in this study. The absence of *J*_polar_ in some simulations may be attributed to the following reasons: some simulations primarily focus on characterizing equatorial current systems (such as neutral sheet currents and eastward ring currents) but insufficiently depict plasma dynamics in polar regions (e.g., polar magnetopause compression and proton injection in cusp regions). *J*_polar_ observed in this study is located in the polar region (0.75 *R*_M_ ≤ *z* ≤ 1.20 *R*_M_) and directly correlates with the plasma pressure gradient in the cusp region, which may not be fully incorporated in some simulations.

The current *J*_polar_ may affect the global magnetic field of Mercury. The cusp is characterized by a prominent diamagnetic depression of the magnetic field, a global feature driven by the high plasma pressure in this region. The *J*_polar_ is a clockwise ring current encircling the north pole, and it arises from the diamagnetic drift of protons in the cusp’s plasma pressure gradient. The clockwise *J*_polar_ current locally enhances the magnetic field inside its ring (closer to the pole) and slightly reduces it outside the ring (in the surrounding cusp). This may be one of the reasons for the observed magnetic field perturbation in the cusp region^42,47^. In addition, the direction of the equivalent magnetic moment of the *J*_polar_ is the same as the direction of the Mercury magnetic moment. If there were north-south asymmetry in the current strength of the *J*_polar_, the Mercury magnetic moment should be shifted northward or southward, depending on the relative magnitude of the current density of *J*_polar_.

The current *J*_polar_ may be coupled with other currents through the FACs. The *J*_ORC_ on the dayside is located above the cusp. When the solar wind dynamic pressure is enhanced, protons can be injected through the cusp region^48–50^. The injection from the cusps into the ring current region^48–50^ may provide carriers for *J*_polar_: the injected protons undergo diamagnetic drift under the action of the polar pressure gradient, enhancing the strength of *J*_polar_, implying that *J*_ORC_ on the dayside may be connected to *J*_polar_ through the FACs. When magnetic reconnection occurs in the magnetotail current sheet, *J*_polar_ may be coupled with the current *J*_ORC_ or *J*_CT_ in the magnetotail through the FACs associated with SCW^19^.

Mercury’s plasma environment is highly dynamic^17,51,52^. The Mercury’s magnetosphere varies in a compressed timescale during Mercury’s substorm^53,54^, suggesting that the temporal SCW should form in a very short timescale. Induction current, another temporal current in planetary interiors^7,40,55,56^, varies with solar wind conditions and has different effects on the dipole moment at different heliocentric distance. Our statistical method, however, removes any possible contribution from temporal variations of the magnetic field, which makes it difficult for the results to characterize these possible temporarily currents, such as SCW and induced current. Therefore, the current systems reconstructed here should be interpreted as large-scale quasi-steady current systems, rather than as a description of transient currents evolving on minute or kinetic timescales. Thus, both SCW and induced currents are not illustrated in Fig. 6. The global steady current systems in Mercury’s magnetosphere shown here reflects the global picture of Mercury’s magnetosphere. As such it cannot represent all the currents in Mercury’s magnetosphere, especially those varying on kinetic scales. However, it is helpful to holistically understand energy conversion process inside Mercury’s magnetosphere and that between Mercury’s magnetosphere and the solar wind. The differences of current systems between Mercury and the Earth imply that planetary environments of different scales will produce very different dynamical processes when interacting with the solar (and beyond, stellar) wind.

In summary, we built the global current systems in Mercury’s magnetosphere based on the magnetic field and plasma measurements by the MESSENGER orbiter. Other than the similarities found in parts of the current systems between Mercury and Earth, e.g., *J*_CT_ and *J*_MP_, an unexpected component like *J*_polar_ is revealed in the Hermean environment. The existence of *J*_polar_ is unexpected, which brings new perspectives to consider in investigating the dynamics of Mercury’s magnetosphere and its energy and mass exchanges with the solar wind. The study of the electric currents in the southern hemisphere, not sampled by the MESSENGER orbits, requires new data expected from the BepiColombo spacecraft upon final insertion in the Mercury orbit in November 2026.

## Methods

MESSENGER entered Mercury orbit in 2011 and concluded operations in 2015. The initial orbit was a 12-h, highly elliptical, near-polar trajectory. For the extended mission, the apoapsis altitude was decreased with periapsis unchanged, resulting in an 8-h orbital period. Along its Mercury orbit, the spacecraft velocity typically ranged from about 0.5–4 km/s, corresponding to ∼0.01–0.10 *R*_M_ per minute. The vector fluxgate magnetometer on MESSENGER provided 20 magnetic field vectors per second^57^. By time averaging of 5 years of those data, we obtain a three-dimensional map of the magnetic field in Mercury’s magnetosphere (Supplementary Fig. 1).

In multi-spacecraft studies, current density is often estimated using the curlometer technique, i.e., by computing the spatial derivatives of the magnetic field based on the Maxwell–Ampère law^58^. In this study, we first reconstruct a gridded three-dimensional average magnetic-field model from five years of single-spacecraft MESSENGER observations, and then estimate current density by applying the first-order central finite difference approximation to $\nabla \times {{{\bf{B}}}}$.

As described by the Maxwell–Ampère law, the curl of the local magnetic field, **B**, at any point in space is shaped by the local current density **J**, i.e.,

2 $$\nabla \times {{{\bf{B}}}}={\mu }_{0}{{{\bf{J}}}}+{\varepsilon }_{0}{\mu }_{0}\frac{\partial {{{\bf{E}}}}}{\partial t}$$

where **E** is electric field vector, *ϵ*_0_*μ*_0_∂**E**/∂t is the displacement current, *ϵ*_0_ and *μ*_0_ are the electromagnetic permittivity and permeability in vacuum, respectively. Considering the non-relativistic regime and assuming electric charge quasi-neutrality, well justified on the large scales of interest, the displacement current contribution can be neglected^59^. Under the assumption that long-term averaging suppresses short-timescale perturbations, the resulting 3D magnetic-field map can be regarded as a representation of the large-scale quasi-steady magnetic configuration of Mercury’s magnetosphere. Therefore, the local current density given by $\frac{1}{{\mu }_{0}}\nabla \times {{{\bf{B}}}}$ provides the “zeroth-order” picture of Mercury’s current systems. The curl is calculated using the first-order central finite difference method for every location in the 3D magnetic field map.

We compared reconstructions using grid spacings of 0.10 *R*_M_ (Supplementary Fig. 13), 0.15 *R*_M_ (Supplementary Fig. 2), and 0.20 *R*_M_ (Supplementary Fig. 14). The large-scale current morphology is broadly consistent between 0.15 *R*_M_ and 0.20 *R*_M_. In contrast, at 0.10 *R*_M_, the number of valid grid cells remaining after quality control decreases substantially, and the reconstructed current pattern becomes more fragmented. We therefore adopt 0.15 *R*_M_ as a compromise between spatial resolution and statistical robustness. We also examined the characteristic proton gyroradii in the relevant regions (Supplementary Fig. 15). They are typically smaller than, or at most comparable to, the adopted grid spacing, supporting the use of 0.15 *R*_M_ for a statistical reconstruction of large-scale current morphology.

The uncertainties of the magnetic field vector components can be estimated after obtaining the average magnetic vector (${\bar{B}}_{x}$, ${\bar{B}}_{y}$, ${\bar{B}}_{z}$). The square of the residuals of the magnetic field measurement is integrated to obtain the standard error of the mean value as^60^

3 $${\sigma }_{{B}_{x}}^{2}=\frac{\mathop{\sum }_{i=0}^{{N}_{B}-1}{\left({B}_{x,i}-{\bar{B}}_{x}\right)}^{2}}{{N}_{B}-1}$$

4 $${\sigma }_{{B}_{y}}^{2}=\frac{\mathop{\sum }_{i=0}^{{N}_{B}-1}{\left({B}_{y,i}-{\bar{B}}_{y}\right)}^{2}}{{N}_{B}-1}$$

5 $${\sigma }_{{B}_{z}}^{2}=\frac{\mathop{\sum }_{i=0}^{{N}_{B}-1}{\left({B}_{z,i}-{\bar{B}}_{z}\right)}^{2}}{{N}_{B}-1}$$

where *σ*_Bx_, *σ*_By_, *σ*_Bz_ are the uncertainties of the respective magnetic field components, *N*_B_ is the amount of the data number.

The uncertainty of each Cartesian current component (*σ*_Jx_, *σ*_Jy_, *σ*_Jz_) is dependent on the uncertainties of the respective magnetic field components (*σ*_Bx_, *σ*_By_, *σ*_Bz_), which we will here consider to be mutually independent. The uncertainty of each Cartesian current component can be derived by

6 $${\sigma }_{{J}_{x}}^{2}=\frac{1}{{\mu }_{0}^{2}}\left({\sigma }_{\frac{\Delta {B}_{z}}{\Delta y}}^{2}+{\sigma }_{\frac{\Delta {B}_{y}}{\Delta z}}^{2}\right)$$

7 $${\sigma }_{{J}_{y}}^{2}=\frac{1}{{\mu }_{0}^{2}}\left({\sigma }_{\frac{\Delta {B}_{x}}{\Delta z}}^{2}+{\sigma }_{\frac{\Delta {B}_{z}}{\Delta x}}^{2}\right)$$

8 $${\sigma }_{{J}_{z}}^{2}=\frac{1}{{\mu }_{0}^{2}}\left({\sigma }_{\frac{\Delta {B}_{y}}{\Delta x}}^{2}+{\sigma }_{\frac{\Delta {B}_{x}}{\Delta y}}^{2}\right)$$

where

9 $${\sigma }_{\frac{\Delta {B}_{x}}{\Delta y}}^{2}\left({x}_{i},{y}_{j},{z}_{k}\right)=\frac{{\sigma }_{{B}_{x}}^{2}\left({x}_{i},{y}_{j-1},{z}_{k}\right)+2{\sigma }_{{B}_{x}}^{2}\left({x}_{i},{y}_{j},{z}_{k}\right)+{\sigma }_{{B}_{x}}^{2}\left({x}_{i},{y}_{j+1},{z}_{k}\right)}{2{\left(\Delta y\right)}^{2}}$$

10 $${\sigma }_{\frac{\Delta {B}_{x}}{\Delta z}}^{2}\left({x}_{i},{y}_{j},{z}_{k}\right)=\frac{{\sigma }_{{B}_{x}}^{2}\left({x}_{i},{y}_{j},{z}_{k-1}\right)+2{\sigma }_{{B}_{x}}^{2}\left({x}_{i},{y}_{j},{z}_{k}\right)+{\sigma }_{{B}_{x}}^{2}\left({x}_{i},{y}_{j},{z}_{k+1}\right)}{2{\left(\Delta z\right)}^{2}}$$

11 $${\sigma }_{\frac{\Delta {B}_{y}}{\Delta x}}^{2}\left({x}_{i},{y}_{j},{z}_{k}\right)=\frac{{\sigma }_{{B}_{y}}^{2}\left({x}_{i-1},{y}_{j},{z}_{k}\right)+2{\sigma }_{{B}_{y}}^{2}\left({x}_{i},{y}_{j},{z}_{k}\right)+{\sigma }_{{B}_{y}}^{2}\left({x}_{i+1},{y}_{j},{z}_{k}\right)}{2{\left(\Delta x\right)}^{2}}$$

12 $${\sigma }_{\frac{\Delta {B}_{y}}{\Delta z}}^{2}\left({x}_{i},{y}_{j},{z}_{k}\right)=\frac{{\sigma }_{{B}_{y}}^{2}\left({x}_{i},{y}_{j},{z}_{k-1}\right)+2{\sigma }_{{B}_{y}}^{2}\left({x}_{i},{y}_{j},{z}_{k}\right)+{\sigma }_{{B}_{y}}^{2}\left({x}_{i},{y}_{j},{z}_{k+1}\right)}{2{\left(\Delta z\right)}^{2}}$$

13 $${\sigma }_{\frac{\Delta {B}_{z}}{\Delta x}}^{2}\left({x}_{i},{y}_{j},{z}_{k}\right)=\frac{{\sigma }_{{B}_{z}}^{2}\left({x}_{i-1},{y}_{j},{z}_{k}\right)+2{\sigma }_{{B}_{z}}^{2}\left({x}_{i},{y}_{j},{z}_{k}\right)+{\sigma }_{{B}_{z}}^{2}\left({x}_{i+1},{y}_{j},{z}_{k}\right)}{2{\left(\Delta x\right)}^{2}}$$

14 $${\sigma }_{\frac{\Delta {B}_{z}}{\Delta y}}^{2}\left({x}_{i},{y}_{j},{z}_{k}\right)=\frac{{\sigma }_{{B}_{z}}^{2}\left({x}_{i},{y}_{j-1},{z}_{k}\right)+2{\sigma }_{{B}_{z}}^{2}\left({x}_{i},{y}_{j},{z}_{k}\right)+{\sigma }_{{B}_{z}}^{2}\left({x}_{i},{y}_{j+1},{z}_{k}\right)}{2{\left(\Delta y\right)}^{2}}$$

where i, j and k are indices of locations in the discretized spatial grids, separated by the respective step sizes Δ*x*, Δ*y* and Δ*z*.

The magnetic field of a dipole component can be written as

15 $${{{\bf{B}}}}\left({{{\boldsymbol{r}}}}\right)=\frac{{\mu }_{0}}{4\pi }\frac{3\left({{{\bf{m}}}}\cdot {{{\bf{r}}}}\right)\hat{{{{\bf{r}}}}}-{{{\bf{m}}}}}{{\left|{{{\bf{r}}}}\right|}^{3}}$$

where **m** is the magnetic moment, **r** is the vector that points from the center of magnetic moment. Mercury’s magnetic moment given by models ranges from 190 to 223 nT *R*_M_^3^ (*R*_M_ is the Mercury’s radius, about 2440 km; *1*, *2*, *3*).

The median relative uncertainty for the current components, defined as the median value of uncertainty of current density divided by the averaged current density, is 5.74, and 82% of the estimated values are determined at 3σ confidence or higher. To estimate the error in the current calculation by the curlometer method, one often refers to the quantity |**∇**·**B** |/|**∇** × **B**|, which should be zero theoretically. However, the estimated ratio can differ significantly from this in practice. In this study, 50% is used as the upper limit of error and all calculations with an error greater than 50% are excluded. Detailed descriptions and the results of the uncertainty calculations are explained below and shown in Supplementary Fig. 16 & Supplementary Fig. 17. It should be noted that the pure offset dipole field (${g}_{1}^{0}$ = −195 nT with a norward offset of 0.196 *R*_M_; *60*), are subtracted before conducting the first-order central finite difference method. One should note that this study used 195 nT R_M_^3^ to maintain consistency with early MESSENGER analysis^61–63^, and different values do not significantly affect the results. In addition, the nominal aberration of the solar wind arises from the combination of Mercury’s orbital velocity and the solar wind speed, resulting in a deflection of approximately 5°–8° in the incident direction of the solar wind relative to Mercury’s radial direction. This fixed-direction deflection is not eliminated by the statistical averaging of multi-year data; instead, it is incorporated as a stable magnetospheric asymmetry into the 5-year average magnetic field model of this study.

Because the Mercury solar magnetic (MSM) coordinates differ from the MSO coordinates only by a constant translation of the origin along the z direction (with parallel basis vectors), this translation does not affect the computed spatial gradients or the resulting current density. We therefore use MSO coordinates for consistency with the native coordinate system of the MESSENGER magnetic-field data.

## Supplementary information

Supplementary Information

Transparent Peer Review file

## Source data

Source Data

## Data Availability

The MESSENGER data used in this study are publicly available from the Planetary Data System (PDS): https://pds-ppi.igpp.ucla.edu/data/mess-mag-calibrated/data/mso/ and https://pds-ppi.igpp.ucla.edu/data/mess-epps-fips-calibrated/data/. Source data are provided with this paper. The data generated in this study are the same as Source data. Source data are provided with this paper.

## Code availability

MESSENGER data are loaded, analyzed, and plotted using MATLAB code package, which can be are publicly available from Zenodo^64^.

## References

[1] Anderson, B. J. et al. Low-degree structure in Mercury’s planetary magnetic field. *J. Geophys. Res.* **117**, E00L12 (2012).

[2] Wardinski, I., Amit, H., Langlais, B. & Thébault, E. The internal structure of Mercury’s core inferred from magnetic observations. *J. Geophys. Res. Planets* **126**, 0–31 (2021).

[3] Genova, A. et al. Geodesy, geophysics and fundamental physics investigations of the BepiColombo mission. *Space Sci. Rev.* **217**, 31 (2021).

[4] Ness, N., Behannon, K., Lepping, R., Whang, Y. C. & Schatten, K. Magnetic field observations near Mercury: preliminary results from Mariner 10. *Science* **185**, 151 (1974). 17810508 10.1126/science.185.4146.151

[5] Solomon, S. C., McNutt, R. L., Gold, R. E. & Domingue, D. L. MESSENGER mission overview. *Space Sci. Rev.* **131**, 3–39 (2007).

[6] Dunlop, M. W., Southwood, D. J., Glassmeier, K. H. & Neubauer, F. M. Analysis of multipoint magnetometer data. *Adv. Space Phys.* **8**, 9–10 (1988).

[7] Glassmeier, K.-H. Currents in Mercury’s magnetosphere. In *Magnetospheric Current Systems* (eds Ohtani, S.I., Fujii, R., Hesse, M. & Lysak, R. L.) (AGU Publications, 2000).

[8] Toepfer, S. et al. The Mie representation for Mercury’s magnetospheric currents. *Earth Planets Space* **73**, 204 (2021).

[9] Zhao, J. T. et al. Observational evidence of ring current in the magnetosphere of Mercury. *Nat. Commun.* **13**, 924 (2022). 35177615 10.1038/s41467-022-28521-3 PMC8854437

[10] Shi, Z. et al. An eastward current encircling Mercury. *Geophys. Res. Lett.* **49**, e2022GL098415 (2022).

[11] Rong, Z. J. et al. The magnetic field structure of Mercury’s magnetotail. *J. Geophys. Res. Space Phys.* **123**, 548–566 (2018).

[12] Anderson, B. J. et al. Steady-state field-aligned currents at Mercury. *Geophys. Res. Lett.* **41**, 7444–7452 (2014).

[13] Slavin, J. A., Owen, J. C. J., Connerney, J. E. P. & Christon, S. P. Mariner 10 observations of field-aligned currents at Mercury. *Planet. Space Sci.* **45**, 133–141 (1997).

[14] Exner, W., Simon, S., Heyner, D. & Motschmann, U. Influence of Mercury’s exosphere on the structure of the magnetosphere. *J. Geophys. Res.: Space Phys.* **125**, e2019JA027691 (2020).

[15] Pump, K., Heyner, D., Schmid, D., Exner, W. & Plaschke, F. Revised magnetospheric model reveals signatures of field-aligned current systems at Mercury. *J. Geophys. Res. Space Phys.* **129**, e2023JA031529 (2024).

[16] Kepko, L., Glassmeier, K.--H., Slavin, J. A. & Sundberg, T. Substorm current wedge at Earth and Mercury. In *Magnetotails in the Solar System* (eds A. Keiling, C. M. Jackman and P. A. Delamere) (2015).

[17] Slavin, J. A. et al. MESSENGER observations of extreme loading and unloading of Mercury’s magnetic tail. *Science* **329**, 665 (2010). 20647422 10.1126/science.1188067

[18] Imber, S. M. & Slavin, J. A. MESSENGER observations of magnetotail loading and unloading: Implications for substorms at Mercury. *J. Geophys. Res. Space Phys.* **122**, 412 (2017). 11, 402–11.

[19] Poh, G. et al. Coupling between Mercury and its nightside magnetosphere: cross-tail current sheet asymmetry and substorm current wedge formation. *J. Geophys. Res. Space Phys.* **122**, 8419–8433 (2017).

[20] Bame, S. J., Asbridge, J. R., Felthauser, H. E., Hones, E. W. & Strong, I. B. Characteristics of the plasma sheet in the Earth’s magnetotail. *J. Geophys. Res.* **72**, 113–129 (1967).

[21] Owen, C. J., Slavin, J. A., Richardson, I. G., Murphy, N. & Hynds, R. J. Average motion, structure and orientation of the distant magnetotail determined from remote sensing of the edge of the plasma sheet boundary layer with E > 35 keV ions. *J. Geophys. Res.* **100**, 185–204 (1995).

[22] Tsyganenko, N. A. et al. Global configuration of the magnetotail current sheet as derived from Geotail, Wind, IMP 8 and ISEE 1/2 data. *J. Geophys. Res.* **103**, 6827–6841 (1998).

[23] Zhong, J. et al. Mercury’s three-dimensional asymmetric magnetopause. *J. Geophys. Res. Space Phys.* **120**, 7658–7671 (2015).

[24] Baumjohann, W. et al. The BepiColombo–Mio magnetometer en Route to Mercury. *Space Sci. Rev.* **216**, 125 (2020).

[25] Heyner, D. et al. The BepiColombo planetary magnetometer MPO-MAG: what can we learn from the hermean magnetic field? *Space Sci. Rev.* **217**, 52 (2021).

[26] Milillo, A. et al. Investigating Mercury’s environment with the two-spacecraft BepiColombo mission. *Space Sci. Rev.* **216**, 93 (2020).

[27] Benkhoff, J. BepiColombo. In: *Encyclopedia of Astrobiology* (eds Gargaud, M. et al.) (Springer, 2023).

[28] Janhunen, P. & Kallio, E. Surface conductivity of Mercury provides current closure and may affect magnetospheric symmetry. *Ann. Geophys.* **22**, 1829–1837 (2004).

[29] Al-Asad, M. M., Johnson, C. L. & Philpott, L. C. Bifurcated current sheets in Mercury’s magnetotail: observations and implications. *J. Geophys. Res. Space Phys.* **126**, e2021JA029417 (2021).

[30] Ganushkina, N. Y. et al. Defining and resolving current systems in geospace. *Ann. Geophys.* **33**, 1369–1402 (2015).

[31] Andrews, G. B. et al. The energetic particle and plasma spectrometer instrument on the MESSENGER spacecraft. *Space Sci. Rev.* **131**, 523–556 (2007b).

[32] Glassmeier, K. et al. Electromagnetic induction effects and dynamo action in the hermean system. *Space Sci. Rev.* **132**, 511–527 (2007).

[33] Slavin, J. A. et al. MESSENGER observations of Mercury’s dayside magnetosphere under extreme solar wind conditions. *J. Geophys. Res. Space Phys.* **119**, 8087–8116 (2014).

[34] Shabansky, V. P. Some processes in the magnetosphere. *Space Sci. Rev.* **12**, 299 (1971).

[35] Walsh, B. M., Ryou, A. S., Sibeck, D. G. & Alexeev, I. I. Energetic particle dynamics in Mercury’s magnetosphere. *J. Geophys. Res.: Space Phys.* **118**, 1992 (2013).

[36] Dewey et al. Energetic electron acceleration and injection during dipolarization events in Mercury’s magnetotail. *J. Geophys. Res. Space Phys.* **122**, 12,170–12,188 (2017).

[37] Antonova, E. E. & Ganushkina, N. Y. U. Inner magnetospheric currents and their role in the magnetosphere dynamics. *Phys. Chem. Earth* **25**, 23–26 (2000).

[38] Antonova, E. E. Investigation of the hot plasma pressure gradients and the configuration of magnetospheric currents from INTER BALL. *Adv. Space Res.* **31**, 1157–1166 (2003).

[39] Antonova, E. E. Magnetostatic equilibrium and current systems in the Earth’s magnetosphere. *Adv. Space Res.* **33**, 752–760 (2004).

[40] Ganushkina, N. Y. U., Liemohn, M. W. & Dubyagin, S. Current systems in the Earth’s Magnetosphere. *Rev. Geophys.* **56**, 309–332 (2018).

[41] Parker, E. N. Newtonian development of the dynamical properties of ionized gases of low density. *Phys. Rev.* **107**, 924–933 (1957).

[42] Raines, J. M. et al. Structure and dynamics of Mercury’s magnetospheric cusp: MESSENGER measurements of protons and planetary ions. *J. Geophys. Res. Space Phys.* **119**, 6587–6602 (2014).

[43] Sun, W. et al. MESSENGER observations of planetary ion enhancements at Mercury’s northern magnetospheric cusp during flux transfer event showers. *J. Geophys. Res. Space Phys.* **127**, e2022JA030280 (2022). 35866073 10.1029/2022JA030280 PMC9286385

[44] Aizawa, S. et al. Cross-comparison of global simulation models applied to Mercury’s dayside magnetosphere. *Planet. Space Sci.* **198**, 105176 (2021).

[45] Dong, C. et al. Global ten-moment multifluid simulations of the solar wind interaction with Mercury: from the planetary conducting core to the dynamic magnetosphere. *Geophys. Res. Lett.* **46**, 11584–11596 (2019).

[46] Lavorenti, F. et al. Electron dynamics in small magnetospheres-Insights from global, fully kinetic plasma simulations of the planet Mercury. *Astron. Astrophys.* **664**, A133 (2022).

[47] Winslow, R. M. et al. Observations of Mercury’s northern cusp region with MESSENGER’s Magnetometer. *Geophys. Res. Lett.* **39**, L08112 (2012).

[48] Delcourt, D. C. et al. Energetic particle injections into the outer cusp during compression events. *Earth Planets Space* **57**, 125 (2005).

[49] Zhao, J. et al. Dynamics of sputtered neutral sodium atoms in the near-Mercury space. *J. Geophys. Res. pace Phys.* **129**, e2023JA032139 (2024).

[50] Jang, E. et al. Energetic ion dynamics near the Cusp region of Mercury. * Astrophys. J.* **892**, 10 (2020).

[51] Slavin, J. A. et al. MESSENGER: exploring Mercury’s magnetosphere. *Space Sci. Rev.* **131**, 133–160 (2007).

[52] DiBraccio, G. A. et al. MESSENGER observations of magnetopause structure and dynamics at Mercury. *J. Geophys. Res. Space Phys.* **118**, 997–1008 (2013).

[53] Sun, W.-J. et al. MESSENGER observations of magnetospheric substorm activity in Mercury’s near magnetotail. *Geophys. Res. Lett.* **42**, 3692–3699 (2015).

[54] Dewey, R. M. et al. MESSENGER observations of flow braking and flux pileup of dipolarizations in Mercury’s magnetotail: evidence for current wedge formation. *J. Geophys. Res. Space Phys.* **125**, e2020JA028112 (2020).

[55] Johnson, C. L. et al. MESSENGER observations of induced magnetic fields in Mercury’s core. *Geophys. Res. Lett.* **43**, 2436–2444 (2016).

[56] Sun, W.-J. et al. Review of Mercury’s dynamic magnetosphere: post-MESSENGER era and comparative magnetospheres. *Sci. China Earth Sci.* **65**, 25–74 (2022).

[57] Anderson, B. J. et al. The magnetometer instrument on MESSENGER. In *The**MESSENGER mission to Mercury* pp. 417– 450 (Springer, 2007a).

[58] Dunlop, M. W., Southwood, D. J., Glassmeier, K. H. & Neubauer, F. M. Analysis of 360 multipoint magnetometer data. *Adv. Space Phys.* **8**, 9–10 (1988).

[59] Sahraoui, F., Belmont, G. & Rezeau, L. Hamiltonian canonical formulation of Hall-magnetohydrodynamics: toward an application to weak turbulence theory. *Phys. Plasmas* **10**, 1325–1337 (2003).

[60] Ramstad, R. et al. The global current systems of the Martian induced magnetosphere. *Nat. Astron.* **4**, 979–985 (2020).

[61] Anderson, B. J. et al. The global magnetic field of Mercury from MESSENGER orbital observations. *Science* **333**, 1859 (2011). 21960627 10.1126/science.1211001

[62] Alexeev, I. I. et al. Mercury’s magnetospheric magnetic field after the first two MESSENGER flybys. *Icarus* **209**, 23 (2010).

[63] Anderson, B. J. et al. The magnetic field of mercury. *Space Sci. Rev.* **152**, 307 (2010).

[64] Lin, R. Global current systems in the magnetosphere of Mercury, marc-lin97/NC_global-current-system-of-Mercury, 10.5281/zenodo.20637391 (2026).

