# Supplementary material for: Global current systems in the magnetosphere of Mercury
Source: Nat Commun. 2026 Jun 23;17:8176. doi: 10.1038/s41467-026-74847-7 (PMC13463068; doi:10.1038/s41467-026-74847-7)
Supplement: Supplementary file 1 [file 41467_2026_74847_MOESM1_ESM.pdf]

# Global current systems in the magnetosphere of Mercury

Rentong Lin[1,6,7], Shiyong Huang[1]✉, Zhigang Yuan[1], James A. Slavin[2], Fouad Sahraoui[3], Jim M. Raines[2], Kui Jiang[1], Lihui Chai[4], Honghong Wu[1], Yue Dong[1], Brian J. Anderson[5], Yunyun Wei[1], Sibo Xu[1], Qiyang Xiong[1], Jian Zhang[1], Zhao Wang[1], Lin Yu[1]

[1]School of Earth and Space Science and Technology, Wuhan University, Wuhan, 430072, China

[2]Department of Climate and Space Sciences and Engineering, University of Michigan, Ann Arbor, MI, USA

[3]Laboratoire de Physique des Plasmas, CNRS-Ecole Polytechnique-Sorbonne Université-Paris-Saclay-Observatoire de Paris-Meudon, Palaiseau, F-91128, France

[4]Key Laboratory of Earth and Planetary Physics, Institute of Geology and Geophysics, Chinese Academy of Sciences, Beijing, 100029, China

[5]The Johns Hopkins University Applied Physics Laboratory, Laurel, Maryland, USA

[6]National Key Laboratory of Deep Space Exploration/School of Earth and Space Sciences, University of Science and Technology of China, Hefei, 230026, China

[7]Center for Excellence in Comparative Planetology/CAS Key Laboratory of Geospace Environment/Mengcheng National Geophysical Observatory, University of Science and Technology of China, Hefei, 230026, China

✉Corresponding author's Email: shiyonghuang@whu.edu.cn

## Supplementary Figures

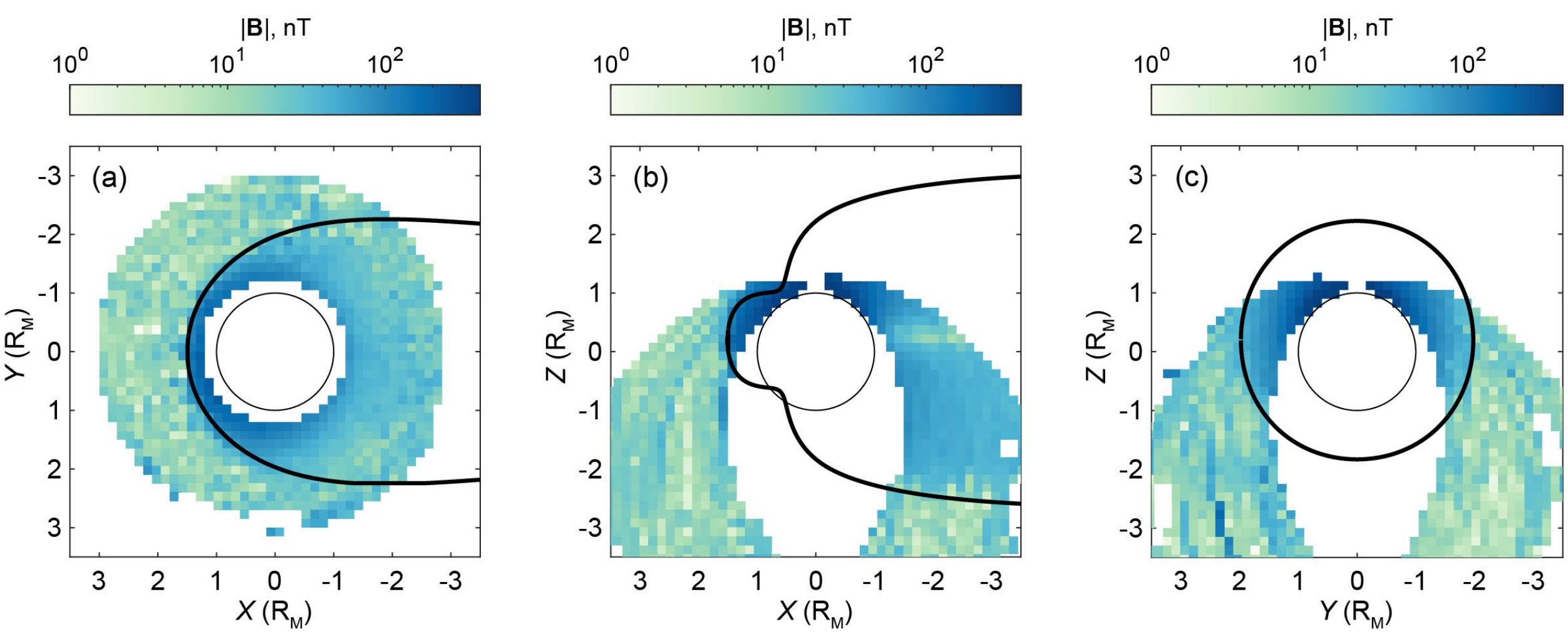

Supplementary Fig. 1. **Slices through the three-dimensional magnetic field of the average Mercury's magnetosphere configuration at the spatial resolution of 0.15 $R_M$. a** A slice in the equatorial plane where z = 0. **b** A slice in the X-Z plane where y = 0. **c** A slice in the Y-Z plane where x = 0. |**B**| is the magnitude of the magnetic field. All slices are 0.15 $R_M$ thick. Each column of panels is colored by the amplitude of the magnetic field. The bolded black lines represent the magnetopause model by Zhong et al. (*63*). The thin black circle in each panel represents the Mercury. The blank areas reflect regions not covered by the MESSENGER orbits (i.e., for z < 0, (x, y) <1 $R_M$, as well as in the z > 1.2 $R_M$). $R_M$ is the Mercury's Radius, about 2440 km.

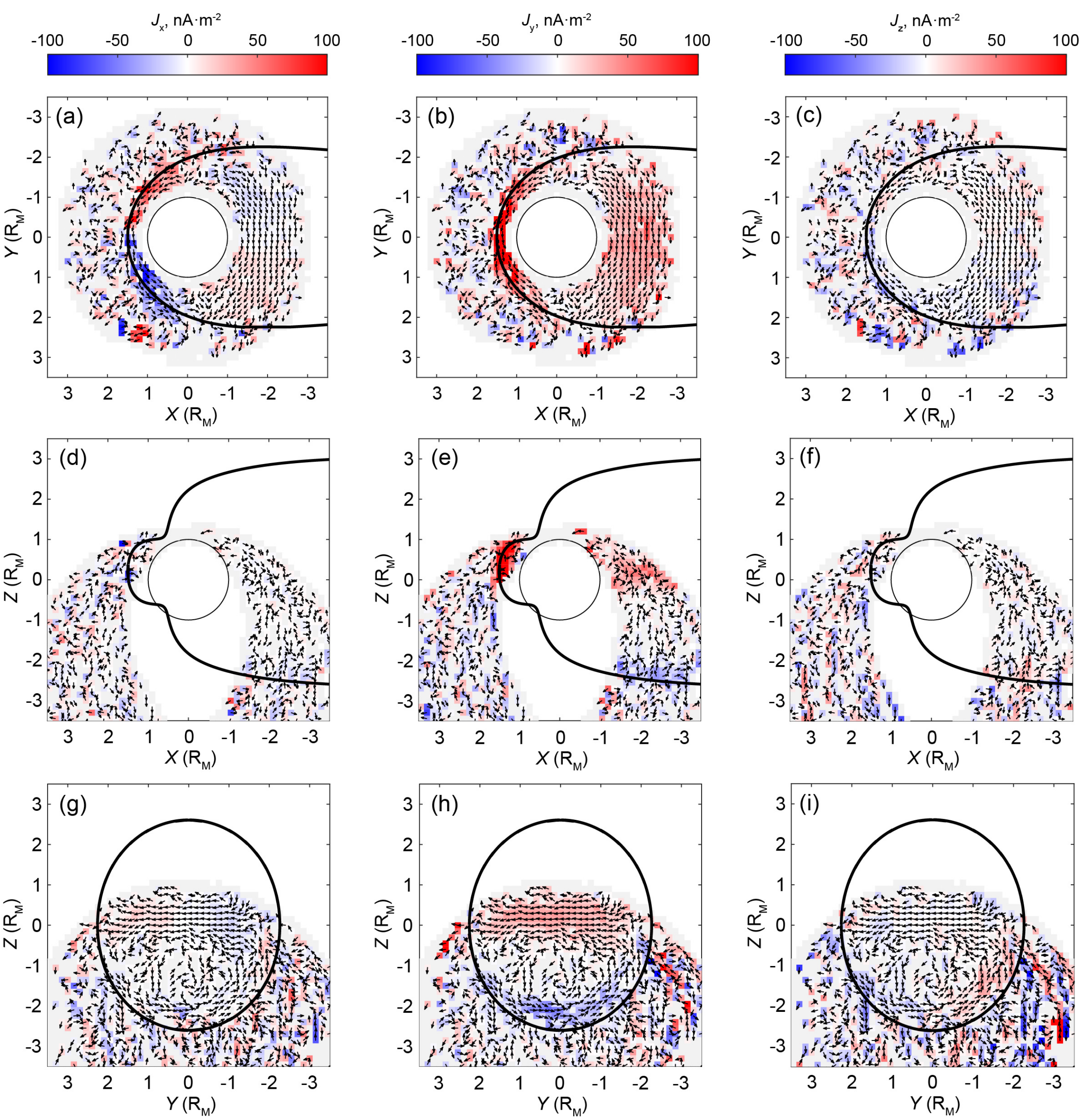

Supplementary Fig. 2. **Slices through the three-dimensional map of the Mercury's current systems at the spatial resolution of 0.15 $R_M$. a–c** The electric current density in equatorial plane within $|z| \leq 0.15$ $R_M$. **d–e** The current in the day–night plane within $|y| \leq 0.15$ $R_M$. **f–i** Slice through the tail at $x = -1.65$ $R_M$ within $-1.80$ $R_M \leq x \leq -1.50$ $R_M$. $J_x$, $J_y$, and $J_z$ are three components of the current density in MSO coordinates. Each column of panels is colored by the components of the current density in MSO coordinates, respectively. Arrows show the current direction in the projected plane of each panel. The thin black circle in each panel represents the Mercury. The bolded black lines in panel a-f and the bolded black circles in panel g-i represent the magnetopause. The grid cells in grey represent no valid measurements. $R_M$ is the Mercury's Radius, about 2440 km.

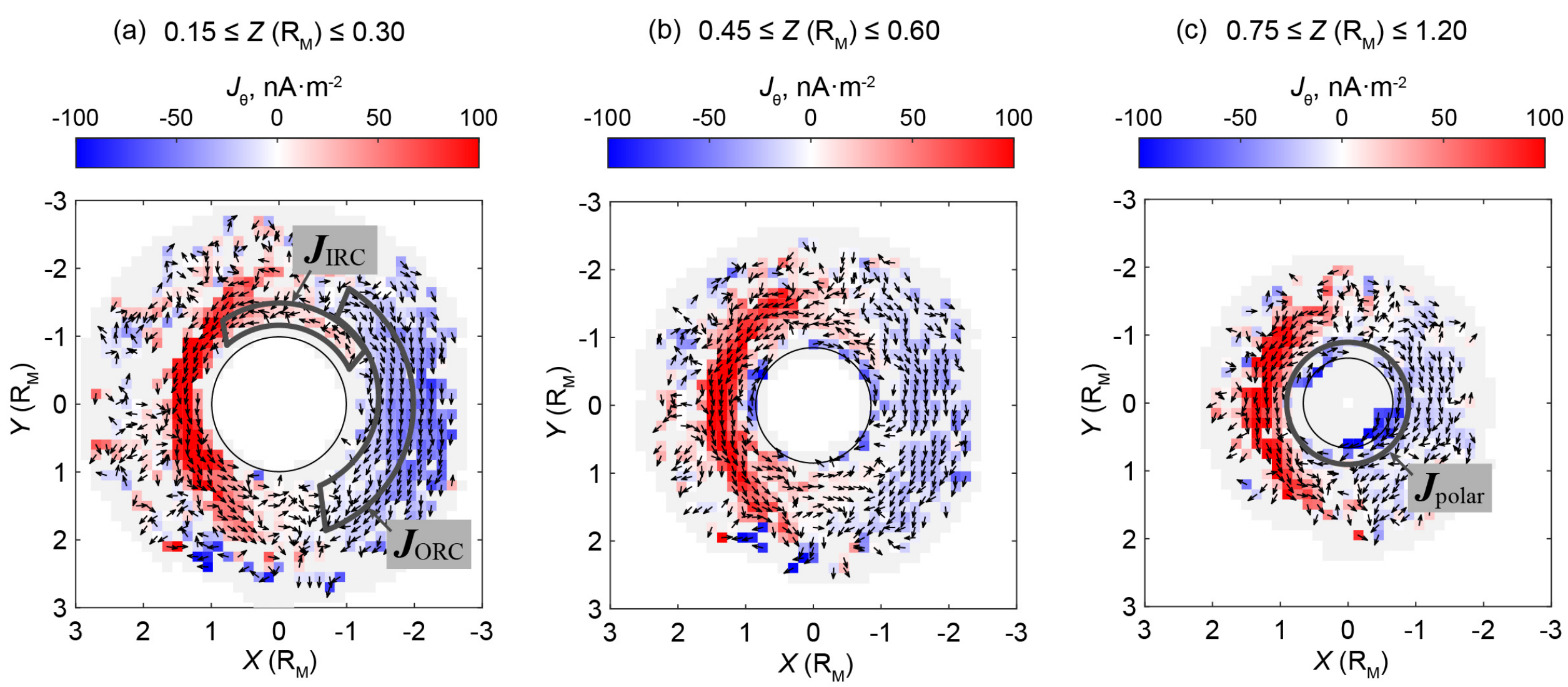


Supplementary Fig. 3. **Current density in X-Y plane within different range of z intervals in cylindrical coordinates. (a)** 0.15 $R_M \leq Z < 0.30$ $R_M$. **(b)** 0.45 $R_M \leq Z < 0.60$ $R_M$. **(c)** 0.75 $R_M \leq Z < 1.20$ $R_M$. $J_\theta$ is the azimuthal component of the current density which is directed tangentially around the z-axis in the direction of increasing azimuthal angle in cylindrical coordinates. $R_M$ is the Mercury's Radius, about 2440 km.

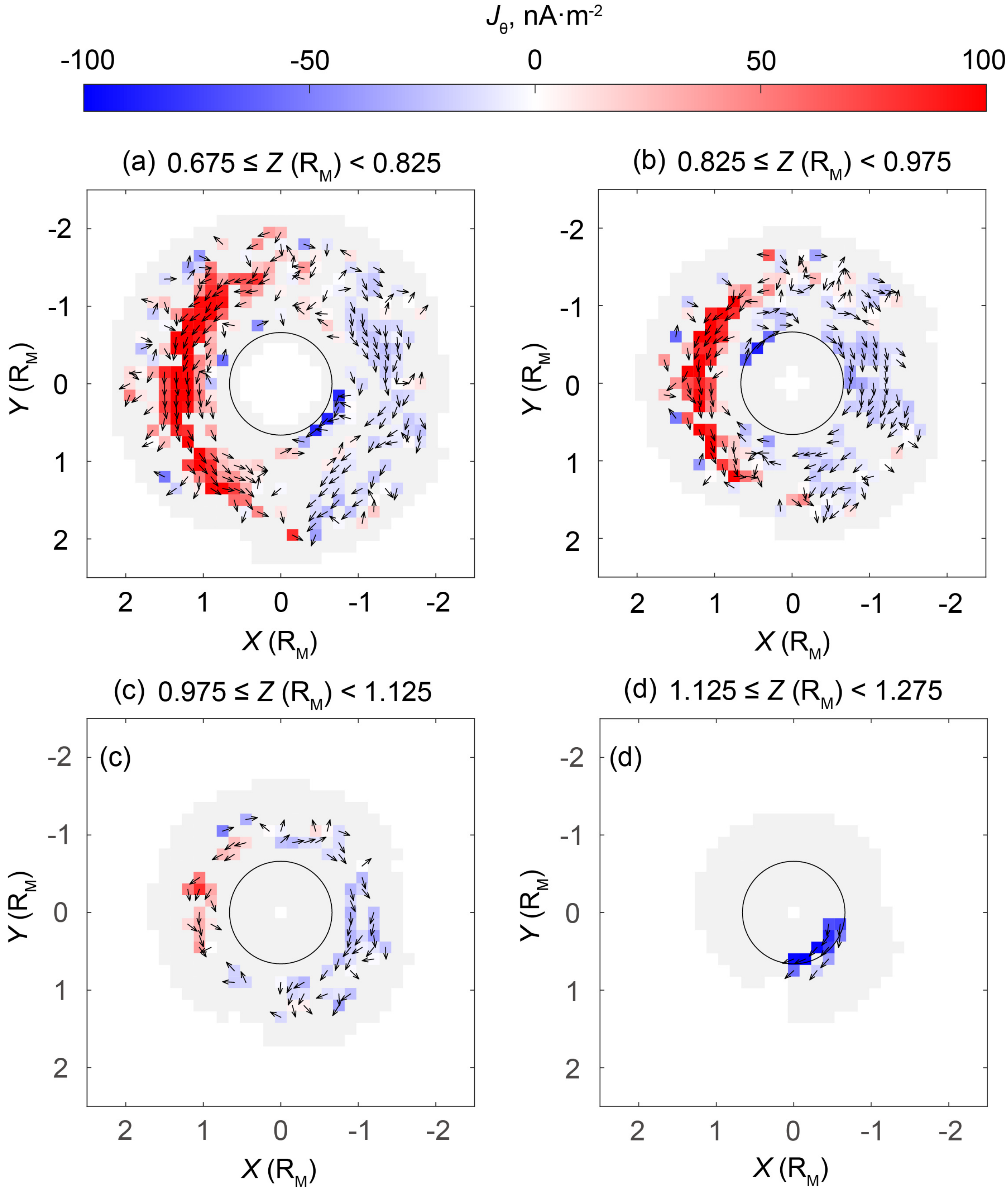


Supplementary Fig. 4. **Current density in size of 0.15 $R_M$ within different range of Z in MSO coordinates.** **(a)** 0.675 $R_M \leq Z < 0.825$ $R_M$. **(b)** 0.825 $R_M \leq Z < 0.975$ $R_M$. **(c)** 0.975 $R_M \leq Z < 1.125$ $R_M$. **(d)** 1.125 $R_M \leq Z < 1.275$ $R_M$. $J_\theta$ is the azimuthal component of the current density which is

directed tangentially around the z-axis in the direction of increasing azimuthal angle in cylindrical coordinates. $R_M$ is the Mercury's Radius, about 2440 km.

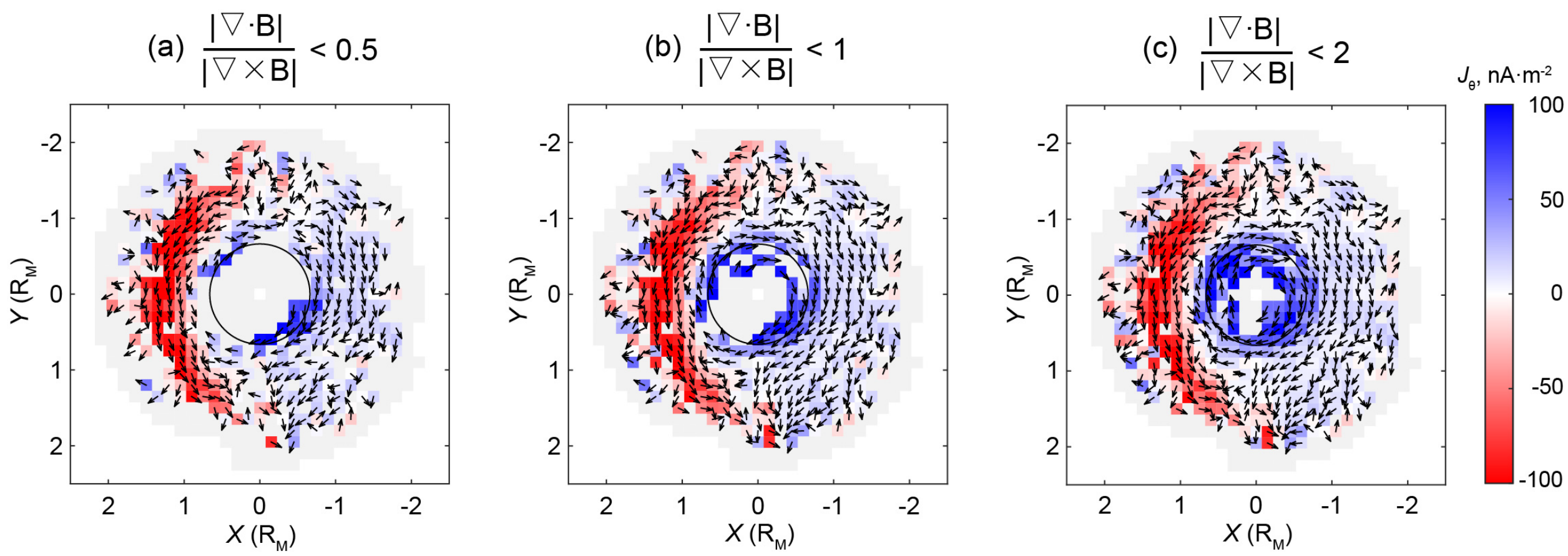


Supplementary Fig. 5. **Current density of the $J_{polar}$ in different error filtering criterion. (a)** error value lower than 0.5. **(b)** error value lower than 1. **(c)** error value lower than 2. $J_\theta$ is the azimuthal component of the current density which is directed tangentially around the z-axis in the direction of increasing azimuthal angle in cylindrical coordinates. $J_\theta$ is the azimuthal component of the current density which is directed tangentially around the z-axis in the direction of increasing azimuthal angle in cylindrical coordinates. $R_M$ is the Mercury's Radius, about 2440 km.

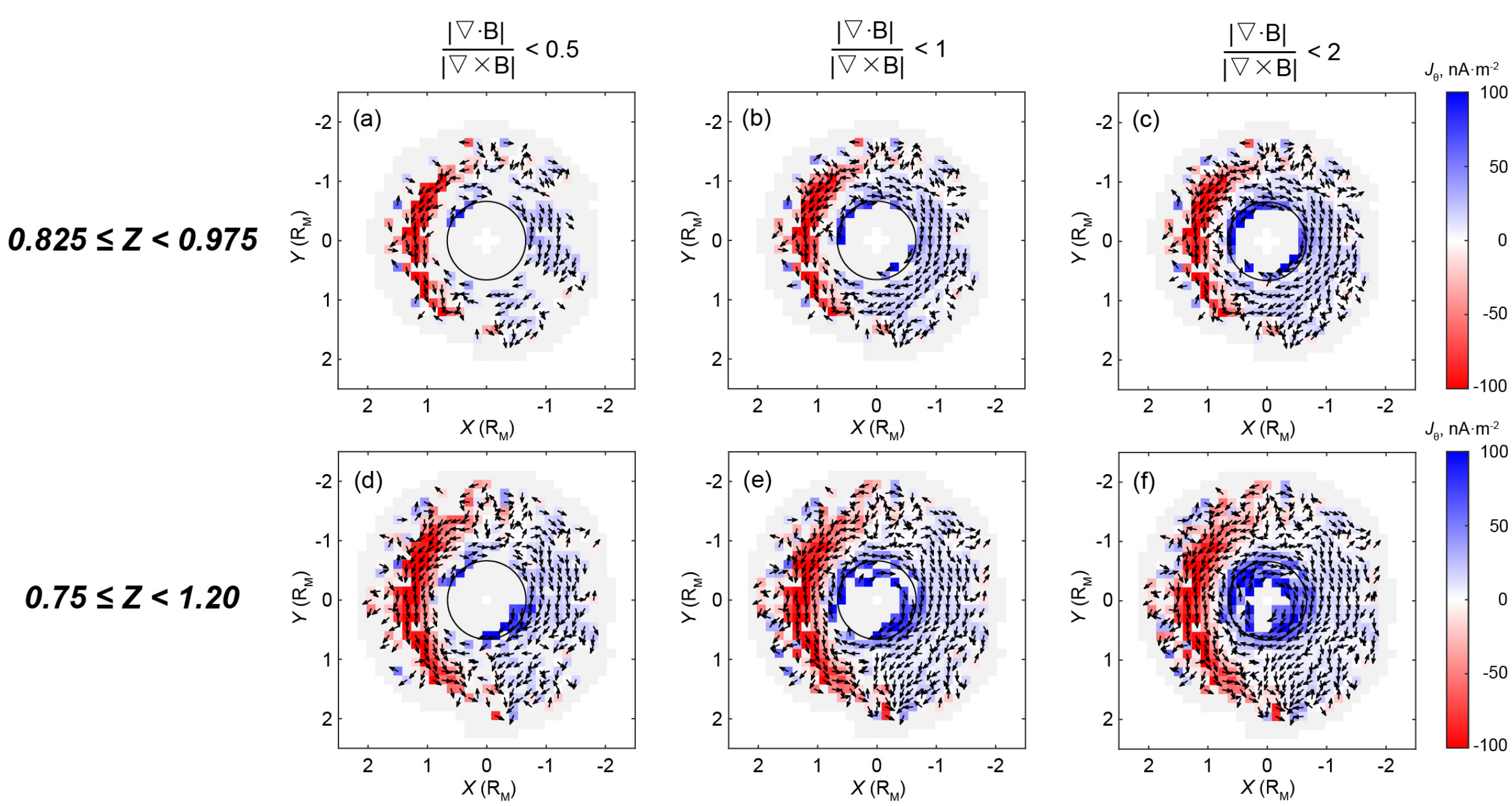


Supplementary Fig. 6. **Current density in size of 0.15 $R_M$ and 0.45 $R_M$ in different error filtering criterion. (a-c)** $0.825\ R_M \leq Z < 0.975\ R_M$. **(d-f)** $0.75\ R_M \leq Z < 1.20\ R_M$. $J_\theta$ is the azimuthal component of the current density which is directed tangentially around the z-axis in the direction

of increasing azimuthal angle in cylindrical coordinates. $R_M$ is the Mercury's Radius, about 2440 km.

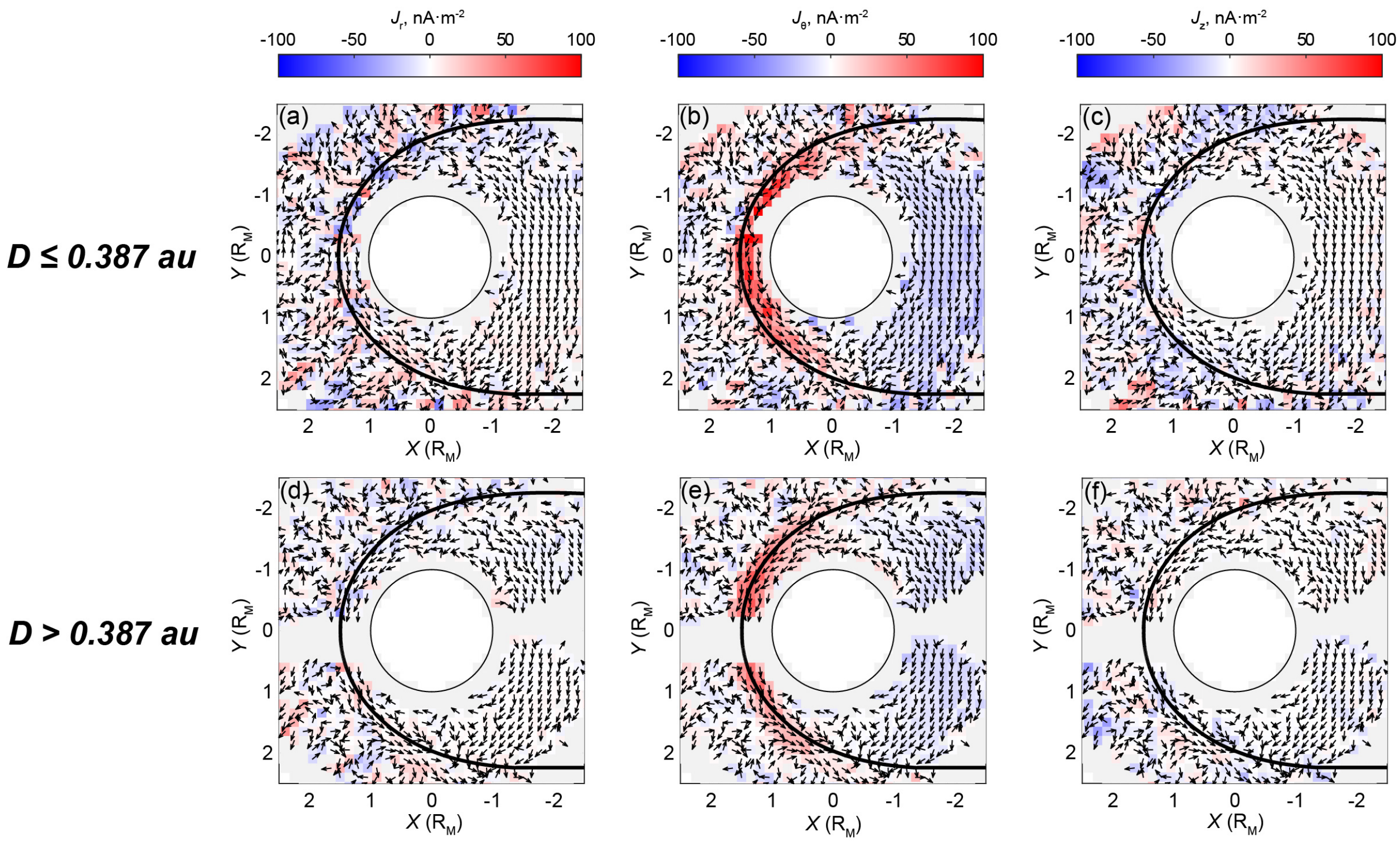


Supplementary Fig. 7. **Magnetopause current at different heliocentric distances. (a-c)** $D \leq 0.387$ au. **(d-f)** $D > 0.387$ au. $D$ is the heliocentric distance. The three mutually orthogonal current density components in the cylindrical coordinate system are the radial component $J_r$ which is directed outward perpendicular to the central longitudinal z-axis, the azimuthal component $J_\theta$ which is directed tangentially around the z-axis in the direction of increasing azimuthal angle, and the axial component $J_z$, which is directed parallel to the central z-axis. $R_M$ is the Mercury's Radius, about 2440 km.

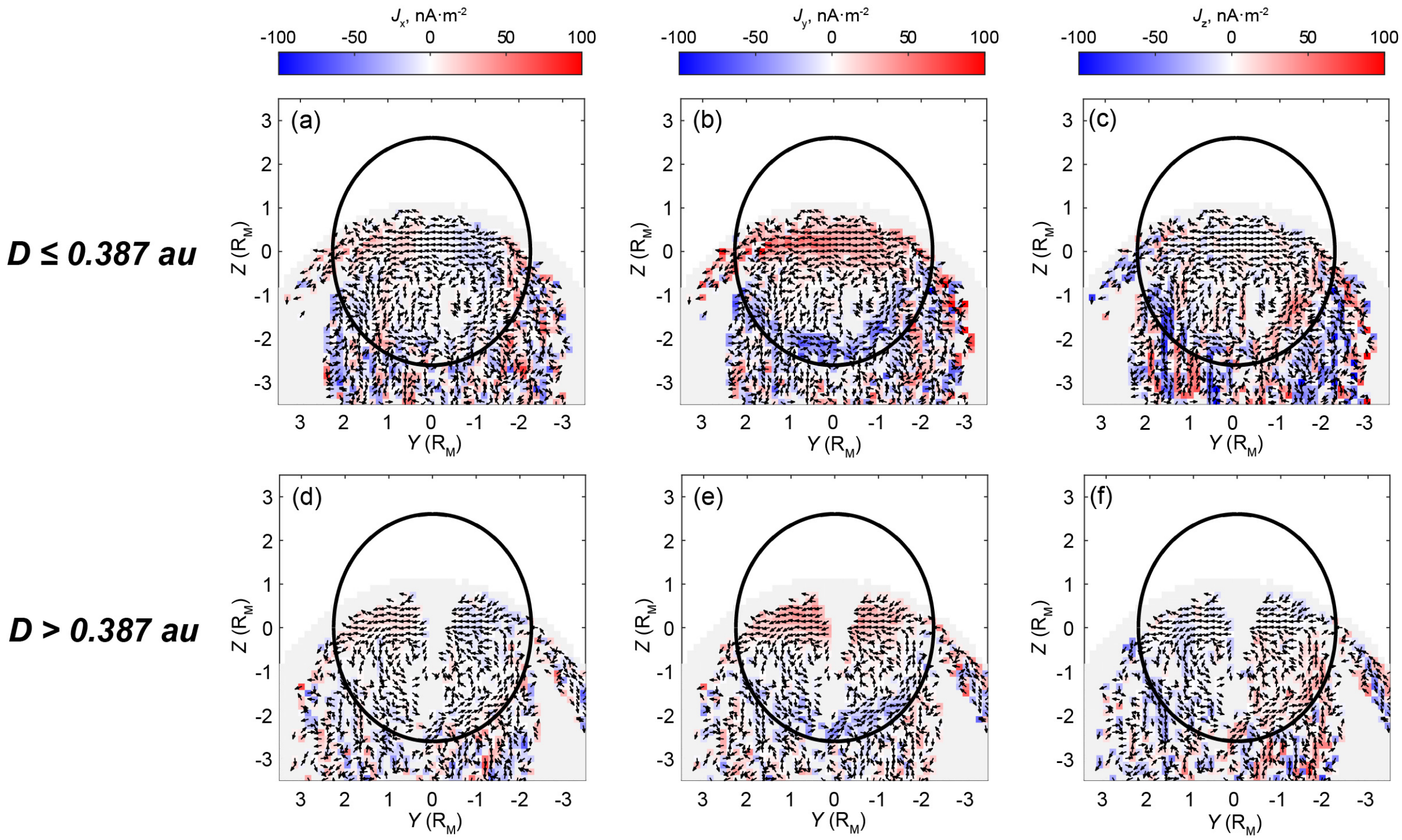


Supplementary Fig. 8. **Cross-tail current at different heliocentric distances. (a-c)** $D \leq 0.387$ au. **(d-f)** $D > 0.387$ au. $D$ is the heliocentric distance. The three mutually orthogonal current density components in the cylindrical coordinate system are the radial component $J_r$ which is directed outward perpendicular to the central longitudinal z-axis, the azimuthal component $J_\theta$ which is directed tangentially around the z-axis in the direction of increasing azimuthal angle, and the axial component $J_z$, which is directed parallel to the central z-axis. $R_M$ is the Mercury's Radius, about 2440 km.

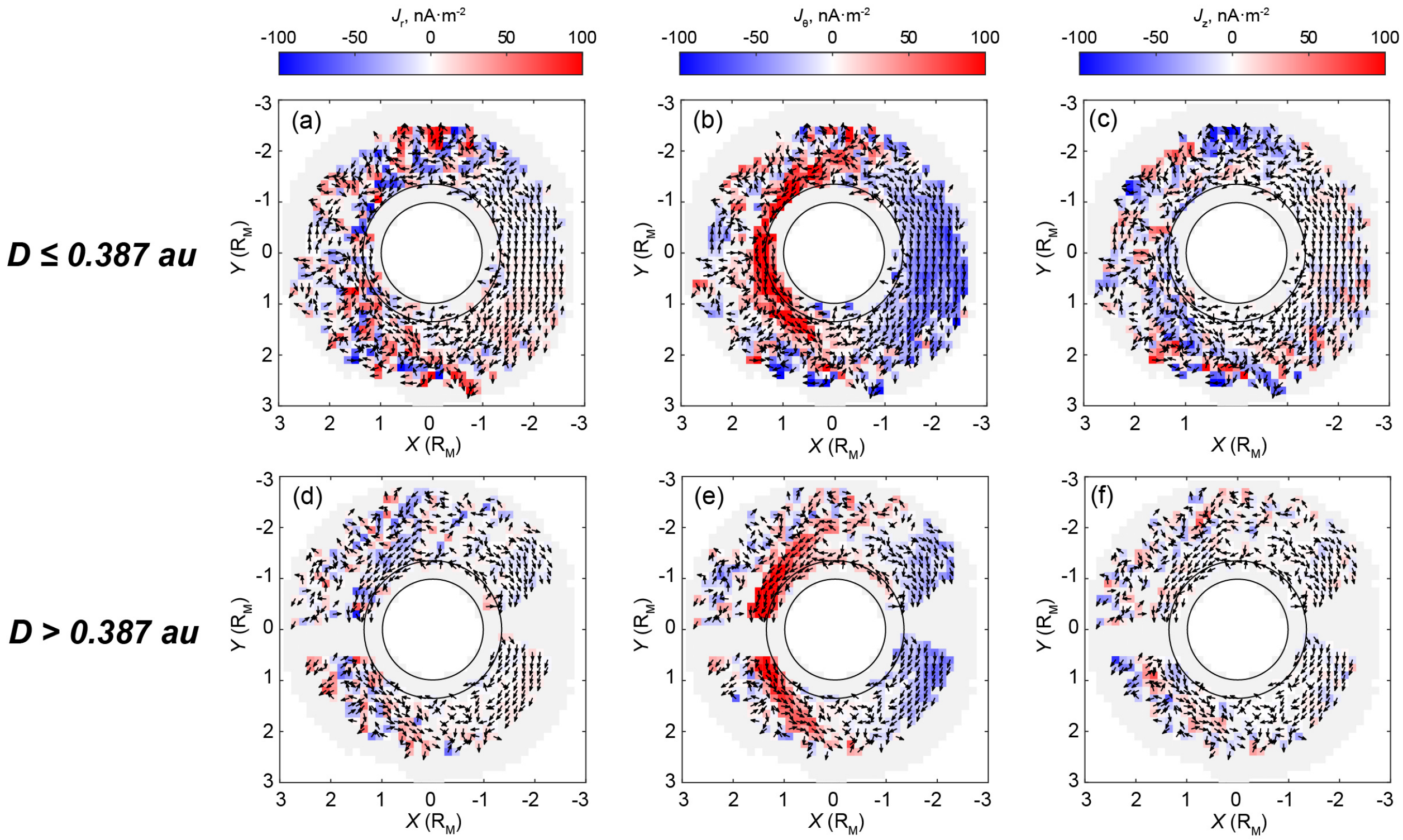


Supplementary Fig. 9. **Equatorial ring current at different heliocentric distances. (a-c)** $D \leq 0.387$ au. **(d-f)** $D > 0.387$ au. $D$ is the heliocentric distance. The three mutually orthogonal current density components in the cylindrical coordinate system are the radial component $J_r$ which is directed outward perpendicular to the central longitudinal z-axis, the azimuthal component $J_\theta$ which is directed tangentially around the z-axis in the direction of increasing azimuthal angle, and the axial component $J_z$, which is directed parallel to the central z-axis. $R_M$ is the Mercury's Radius, about 2440 km.

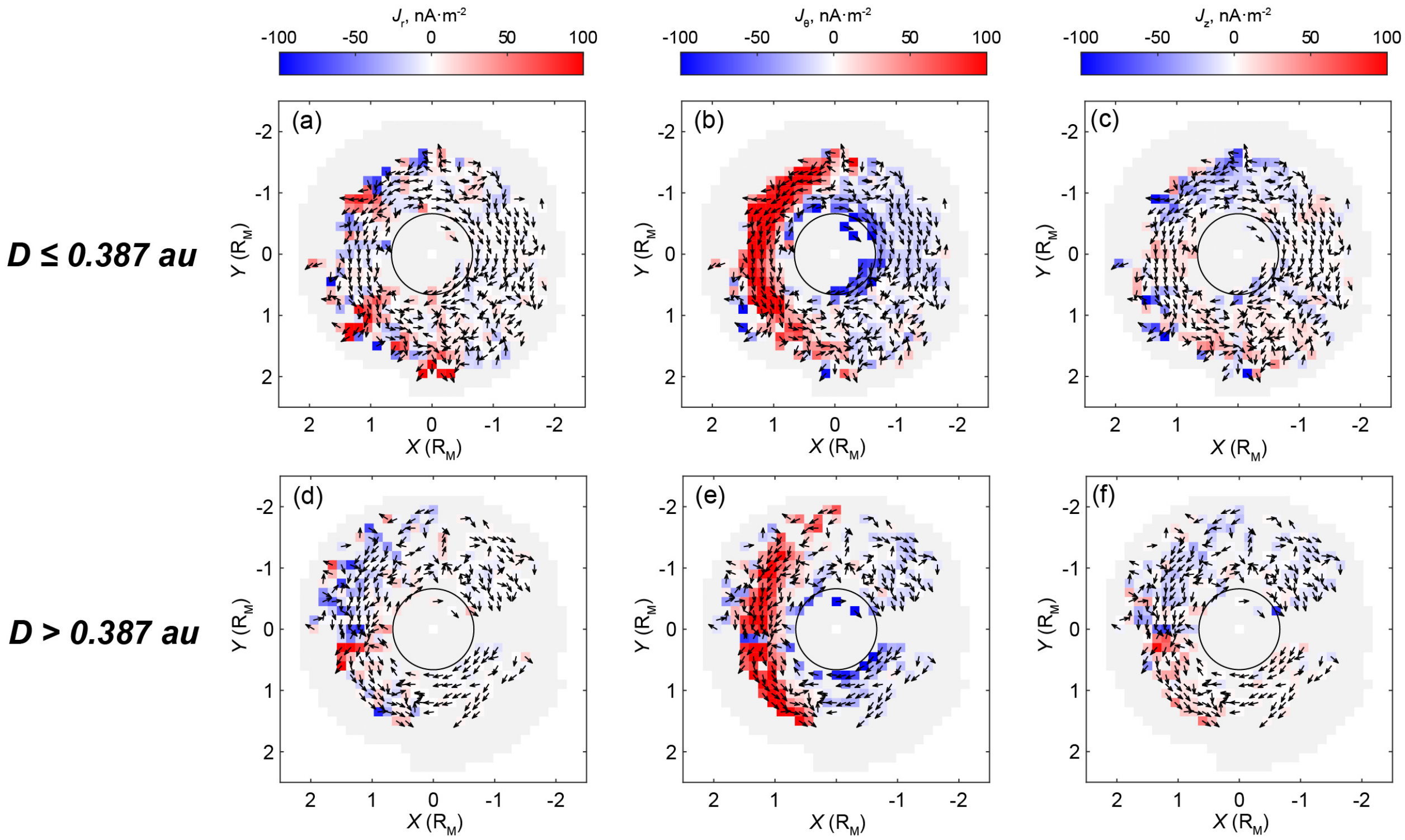


Supplementary Fig. 10. **Polar ring current at different heliocentric distances. (a-c)** $D \leq 0.387$ au. **(d-f)** $D > 0.387$ au. $D$ is the heliocentric distance. The three mutually orthogonal current density components in the cylindrical coordinate system are the radial component $J_r$ which is directed outward perpendicular to the central longitudinal z-axis, the azimuthal component $J_\theta$ which is directed tangentially around the z-axis in the direction of increasing azimuthal angle, and the axial component $J_z$, which is directed parallel to the central z-axis. $R_M$ is the Mercury's Radius, about 2440 km.

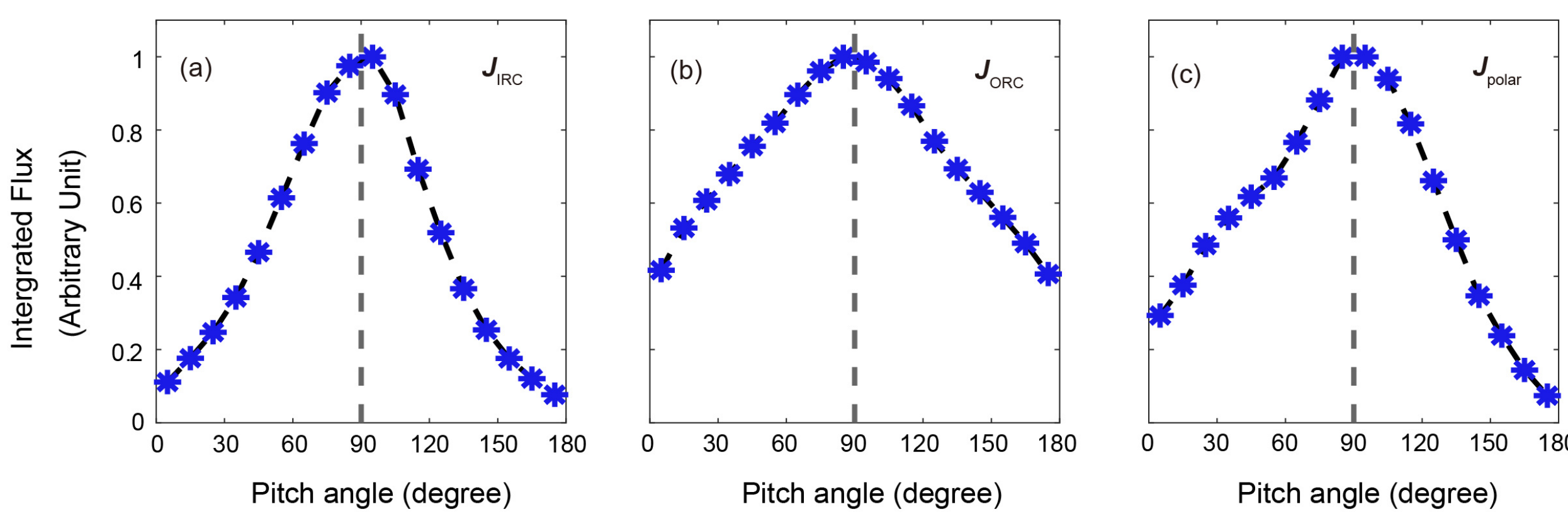


Supplementary Fig. 11. **Pitch angle distributions of protons.** Pitch angle distributions of protons derived from FIPS measurements within regions consistent with **(a)** the inner ring current $\boldsymbol{J}_{IRC}$, **(b)**

the outer ring current $\boldsymbol{J}_{\mathrm{ORC}}$ and **(c)** the polar ring current $\boldsymbol{J}_{\mathrm{polar}}$, respectively. The dashed lines represent the pitch angle at 90 degrees.

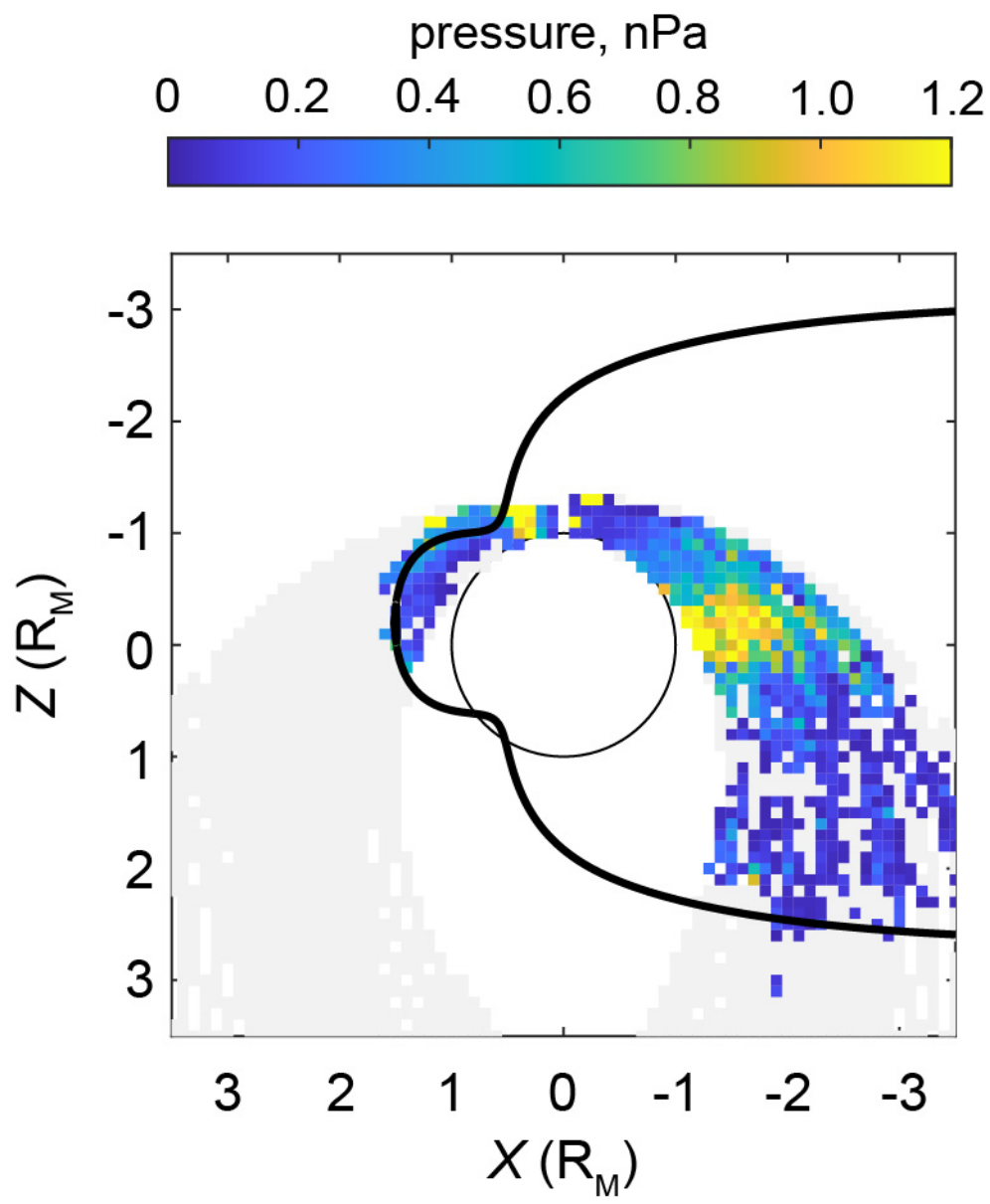


Supplementary Fig. 12. **Distribution of plasma pressure of $H^+$ ions in X-Z plane centered at Y=0.** Distribution of plasma pressure derived from the Fast Image Plasma Spectrometer (FIPS) measurements (*30*). The grid cells in grey represent no valid measurements. $R_M$ is the Mercury's Radius, about 2440 km.

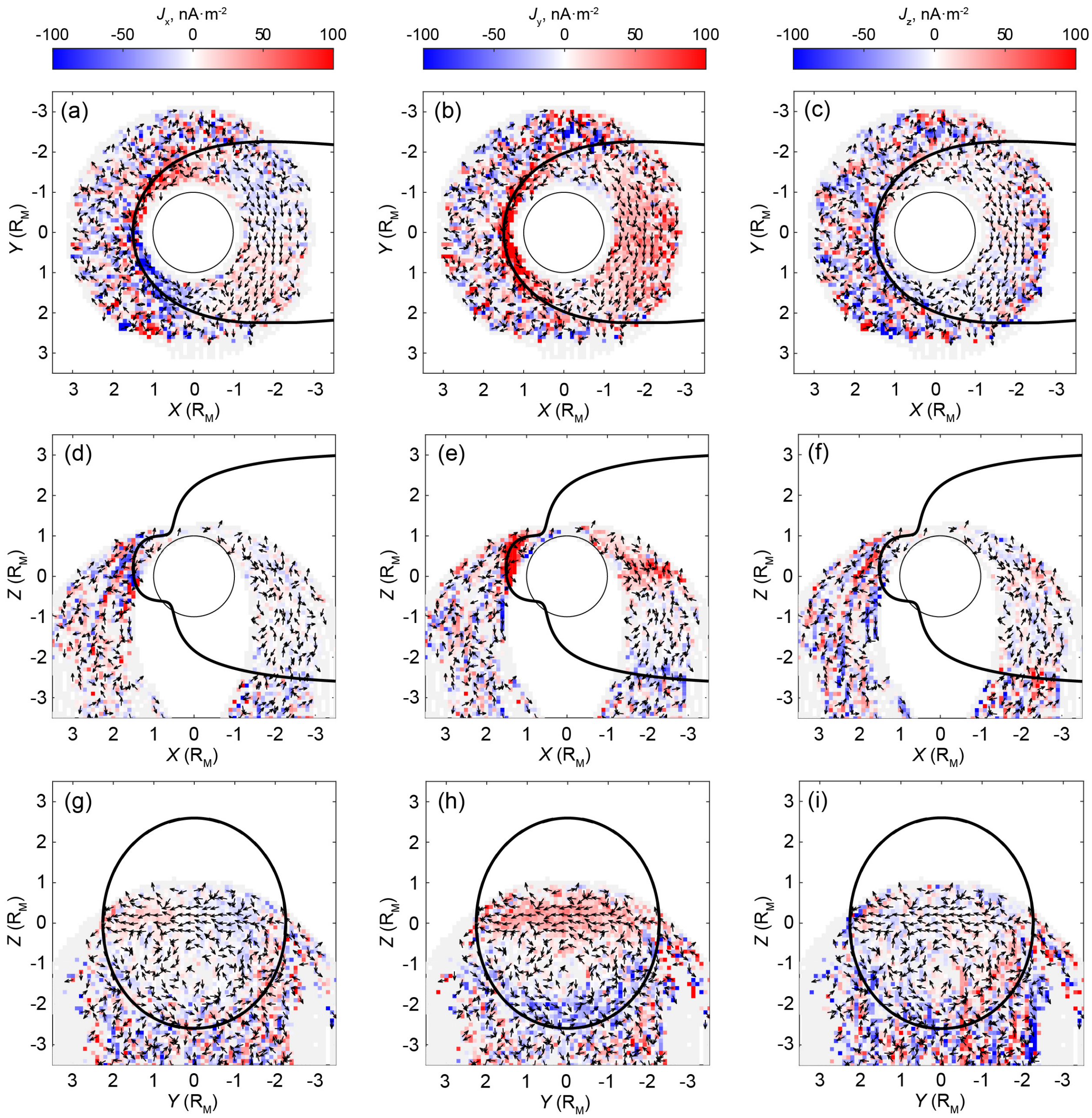


Supplementary Fig. 13. **Slices through the three-dimensional map of the Mercury's current systems at the spatial resolution of 0.10 R$_M$.** Panels **a-c** show the electric current density in equatorial plane. Panels **d-f** show the current in the day–night plane, and panels **g-i** show a slice through the tail at x = -1.60 R$_M$. Each column of panels is colored by the components of the current density in MSO coordinates, respectively. Arrows show the current direction in the projection plane of each panel. The thin black circle in each panel represents the Mercury. The bolded black lines in panel a-f and the bolded black circles in panel g-i represent the magnetopause. R$_M$ is the Mercury's Radius, about 2440 km.

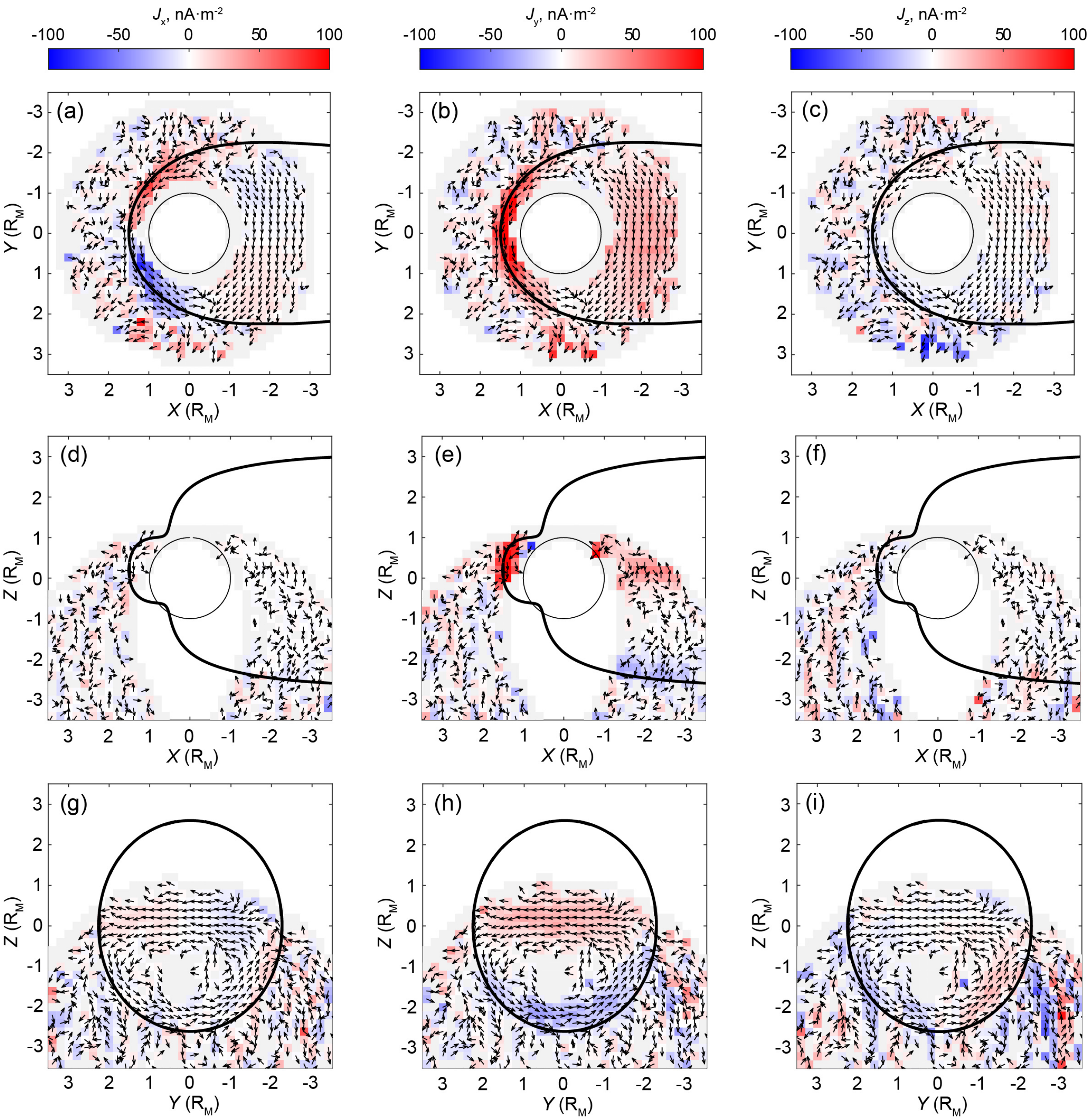


Supplementary Fig. 14. **Slices through the three-dimensional map of the Mercury's current systems at the spatial resolution of 0.20 R$_M$.** Panels **a-c** show the electric current density in equatorial plane. Panels **d-f** show the current in the day–night plane, and panels **g-i** show a slice through the tail at x = -1.60 R$_M$. $J_x$, $J_y$, and $J_z$ are three components of the current density in MSO coordinates. Each column of panels is colored by the components of the current density in MSO coordinates, respectively. Arrows show the current direction in the projection plane of each panel. The thin black circle in each panel represents the Mercury. The bolded black lines in panel a-f and

the bolded black circles in panel g-i represent the magnetopause. $R_M$ is the Mercury's Radius, about 2440 km.

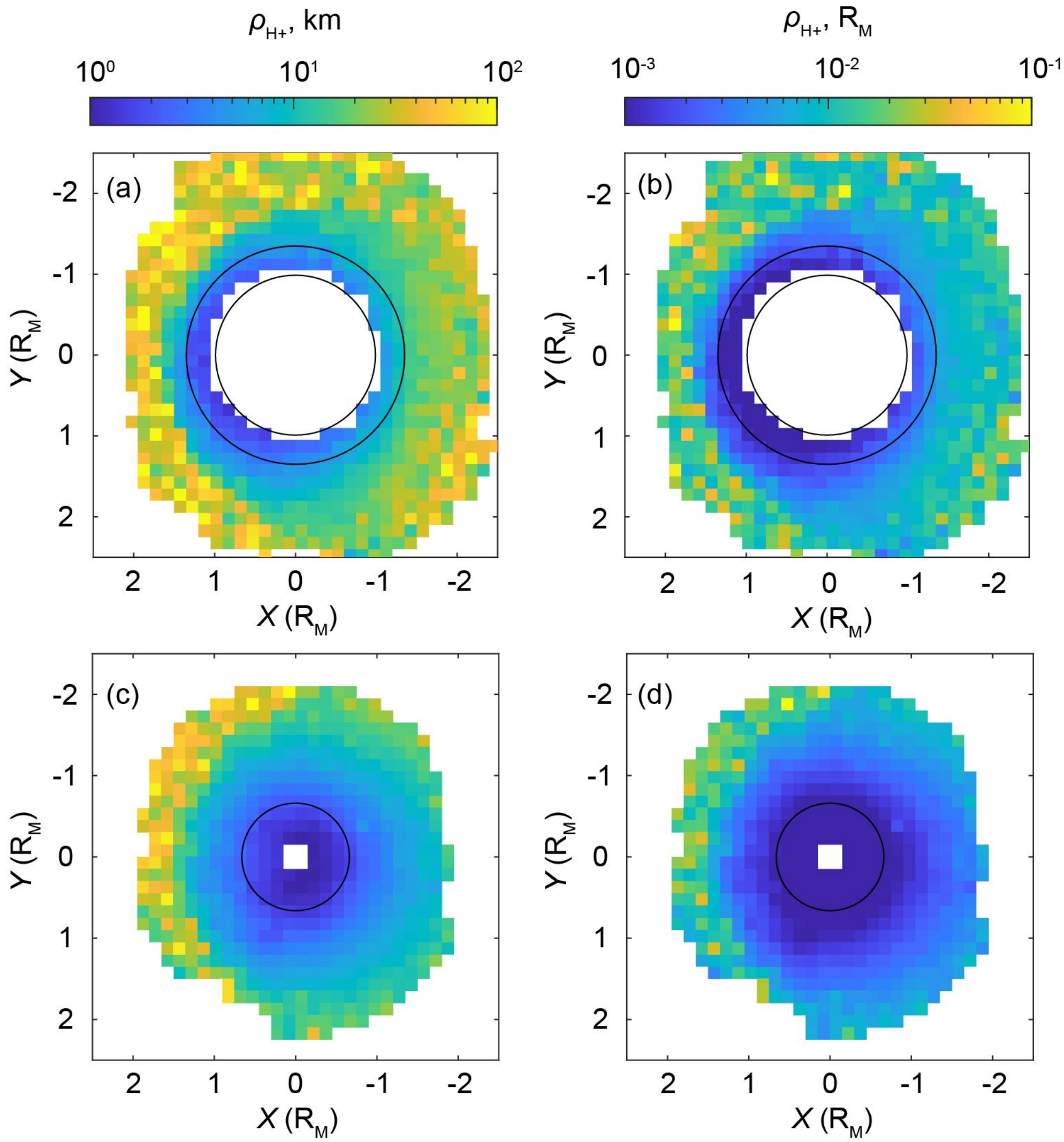


Supplementary Fig. 15. **Gyroradii of proton in X-Y plane. (a-b)** Within regions of $\boldsymbol{J}_{ORC}$. **(c-d)** within regions of $\boldsymbol{J}_{polar}$. $\rho_{H+}$ is the gyroradii of proton. $R_M$ is the Mercury's Radius, about 2440 km.

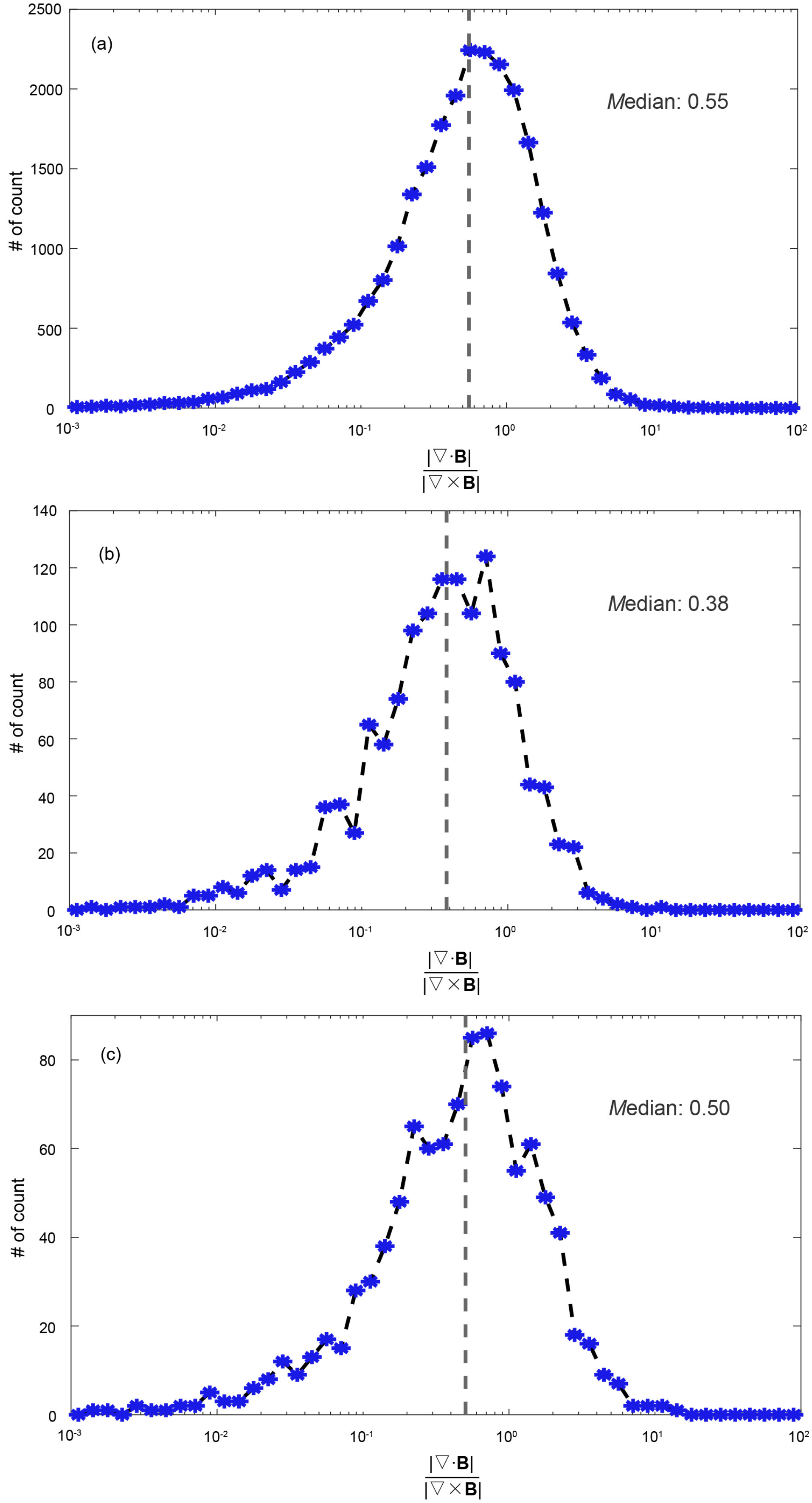
(a)
Median: 0.55
# of count
2500
2000
1500
1000
500
0
$10^{-3}$
$10^{-2}$
$10^{-1}$
$10^{0}$
$10^{1}$
$10^{2}$
$\frac{|\nabla \cdot \mathbf{B}|}{|\nabla \times \mathbf{B}|}$
(b)
Median: 0.38
# of count
140
120
100
80
60
40
20
0
$10^{-3}$
$10^{-2}$
$10^{-1}$
$10^{0}$
$10^{1}$
$10^{2}$
$\frac{|\nabla \cdot \mathbf{B}|}{|\nabla \times \mathbf{B}|}$
(c)
Median: 0.50
# of count
80
60
40
20
0
$10^{-3}$
$10^{-2}$
$10^{-1}$
$10^{0}$
$10^{1}$
$10^{2}$
$\frac{|\nabla \cdot \mathbf{B}|}{|\nabla \times \mathbf{B}|}$

Supplementary Fig. 16. **Distributions of relative deviation from Gauss' law for the magnetic field divergence.** The relative divergence **(a)** including all grid cells, **(b)** within 0.15 $R_M \leq z \leq 0.30$ $R_M$, and **(c)** within 0.75 $R_M \leq z \leq 1.20$ $R_M$. **B** is magnetic field vector. The dashed lines represent the median.

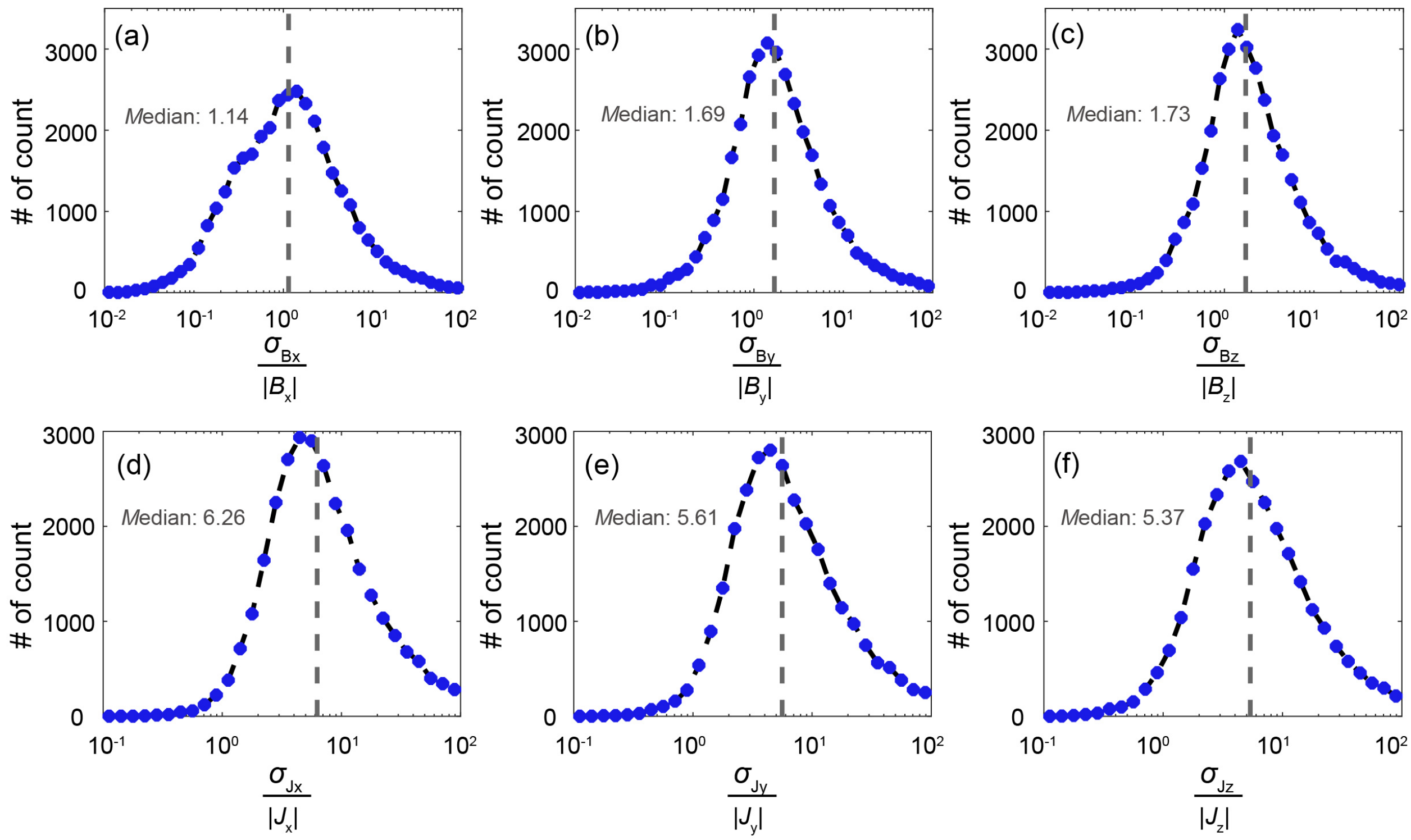


Supplementary Fig. 17. **Distribution of relative errors.** Distribution of relative errors of each magnetic field components **(a-c)** and current density components **(d-f)**. Dashed lines represent the median value.
