# Supplementary material for: Global current systems in the magnetosphere of Mercury
Source: Nat Commun. 2026 Jun 23;17:8176. doi: 10.1038/s41467-026-74847-7 (PMC13463068; doi:10.1038/s41467-026-74847-7)
Supplement: Supplementary file 2 [file 41467_2026_74847_MOESM2_ESM.pdf]

nature portfolio

Peer Review File

Global current systems in the magnetosphere of Mercury

Corresponding Author: Professor Shiyong Huang

This file contains all reviewer reports in order by version, followed by all author rebuttals in order by version.

Version 0:

Reviewer comments:

Reviewer #1

(Remarks to the Author)
This paper uses magnetic field measurements obtained by MESSENGER spacecraft to describe the Hermean magnetospheric current systems. Authors used the curlometer technique to find current densities along MESSENGER orbits. This technique is applied to MESSENGER mission data for the first time, thus the obtained results are rather novel, for example the polar ring current existence and two different equatorial ring currents existence have been shown. Nevertheless there are issues to be clarified. The curlometer technique was initially developed to analyze the multi-spacecraft measurements (Dunlop et al., 2021, 10.1029/2021JA029538). Using the single-spacecraft data should be carried with even more than usual caution, because there are many factors which can lead to a change of magnetic field, which can be interpreted as an electric current using this method.

The following are the comments and questions about the manuscript:

1. Why magnetospheric current systems are analysed in MSO (Mercury Solar Orbiter) coordinate system, but not in MSM (Mercury Solar-Magnetospheric) coordinate system?

2. A grid spacing of 0.15 RM is used in the study, which has been chosen from consiredation of 0.1 RM and 0.2 RM. But, the Hermean magnetosphere is very dynamic (Slavin et al., 2010). Thus on a timescale of minutes magnetospheric state can change significantly. What was the distance covered by MESSENGER on a timescale of minutes? If it is less than 0.15 RM, then the change of magnetic field could come from the reconfiguration of the magnetosphere. Also, what is the size of the gyro-radius for the described spatial regions comparing to a grid spacing? Slavin et al. (2008, 10.1126/science.1159040) mentioned that for example double magnetopause signature has been observed at Mercury and "for Na+, the depth of penetration into the magnetosphere would be ~1 gyro-radius or ~1000 km", which can influence the observations. Thus, the choise of 0.1, 0.15 and 0.2 RM grid spacing values should be justified better.

3. As it has been mentioned, Hermean magnetosphere is highly dynamic. The subsolar magnetopause distance can change significantly (see for example Zhong et al., 2015, 10.1002/2015GL067063). Sometimes it can almost disappear (Slavin et al., 2019, 10.1029/2019JA026892). Thus at the same point at MSO coordinate system for different spacecraft orbits there may be very different conditions. This should be mentioned in the text. But at this manuscript all measurements are studied at the same time and are compared with a fixed magnetopause surface. Does it influence the obtained results?

4. The main findings are made from the figures with current densities maps, but the detected currents are not indicated in any way in the figures. For example, for J_orc: "As shown in Figure 3a & 3b, there exists a clockwise current in the near-Mercury magnetotail (-1.4 RM < x < 0)". But on the figure the curved tail currents are obviously seen, like on figure 3 at Ganushkina et al. (2018, 10.1002/2017RG000590). Please, highlight the areas which you consider for J_orc. The same could be done for J_irc, although it is more visible. Also, at Figure 4, the shown arrows in the nighside are located much above the equatorial plane. Please, show the magnetic equatorial plane at figure 4 to make it more clear, that J orc is located close to magnetic equator in the nightside.

5. Slices for figures 2a-f and 3a-c have a reasonable size of 0.15 RM, which is a grid step. The slice for J_polar has a size of 0.45 RM (0.75 RM ≤ z ≤ 1.20 RM). It is three times larger than the grid step. Please, explain the choice of this range. J_polar should be highlighted as well and maybe it is reasonable to show it in a narrower slice to describe its actual location.

6. Figures 5 and extended 5 are very important in describing the nature of J_irc and J_orc. Please highlight the IRC and

ORC at the figure 5.

7. J_irc looks similar to eastward current found by Shi et al. (2022, 10.1029/2022GL098415). Please, specify it in 'Physical nature of the current systems' section.

8. Poh et al. (2017, 10.1002/2016GL071612) have shown that the inner current sheet edge location at Mercury is located at x = −1.22 RM. The location of plasma pressure peak in the manuscript should be compared with this value. If they are comparable, does it mean that J_orc, which is located further from the planet from this point and J_ct are located inside each other in the nightside?

Reviewer #2

(Remarks to the Author)
The authors analyzed five years of MESSENGER spacecraft data to derive a three-dimensional pattern of Mercury's magnetic fields. The global current system was then calculated by the first-order central finite difference method in the polar region as well as in the equatorial region. Various currents were identified: magnetopause current, cross-tail current, inner and outer equatorial ring current, and polar ring current. The polar ring current was discovered by the authors. If these authors' claim would be robust, their finding would advance understanding dynamics of the Mercury's magnetosphere.

However, there are unclear parts in the authors' analysis. The first one is identification of the polar ring current. In Figures 3d and 3e, the polar ring current appears at prenoon and premidnight local time near the Mercury's surface depicted with thin black circles. The current does not constitute a circular shape which is usually the case for the ring current. The authors divided the data set referring to the Mercury's heliocentric distance D and performed the same statistics (Extended Data Figure 3). There is some difference between the distribution of the current in Figures 3d and 3e and those in Extended Data Figure 3, particularly at prenoon. Therefore, the statistical significance of the polar ring current would not be large enough.

The second part is interpretation of the polar ring current in terms of pressure gradient in the cusp region. The authors related these two quantities. However, the derived location of the polar ring current at prenoon and premidnight in Figures 3d and 3e or the anticipated location of the current at the whole local time does not match with that of the cusp, which is prenoon as well as postnoon (Sun et al., 2022; Winslow et al., 2012; both referred in the manuscript). Therefore, the mechanism suggested by the authors would not work, depending on local time.

The third part is the relation between the outer equatorial ring current and the polar ring current (Figure 6). Even though both flow off the equator in the dayside, they were treated as different current. I wonder whether both would be the same, if these currents would really exist. The authors identified the outer equatorial ring current from Figure 4. However, it is hard to identify current from this figure because the plot is a three-dimensional map. Moreover, the upward current in the duskside and the downward current in the dawnside, part of the outer equatorial ring current, cannot be identified in Figure 3.

In summary, the uncertainty in the derived current pattern would be large so that the polar ring current could not be identified. This current may not be explained by the pressure gradient near the cusp because the local time distribution of this current is different from that of the cusp. Perhaps, BepiColombo data, which would be available in the near future, would be needed for further analysis. Nevertheless, trial to derive the current perpendicular to the background magnetic field would be new in the polar region. I feel the manuscript would be of interest to certain community if it is appropriately revised. Because of this, I have added general and specific comments as listed below.

As a general comment, the authors derived currents in Cartesian coordinates (e.g., Figures 2 and 3). If this would be presented in cylindrical coordinates, identification of ring current or partial ring current would be easier.

The second comment is the methodology the authors used to derive the current. Although the authors mentioned it as the curlometer technique, this is usually applied to four spacecraft measurements (Dunlop et al., 1988). Instead, the authors applied the first-order central finite difference method to the Cartesian grid data as performed in Shi et al. (2022). Therefore, the technique the authors employed could be noted as the latter.

The third comment is regarding field-aligned currents (FACs). Previous reports such as Pump et al. (2024) identified FACs from MESSENGER magnetic field observations in the polar region. Is it possible that the currents identified in the polar region by the authors are FACs instead of ring currents?

Specific comments

Line 54. when in -> during

Lines 82-84. Figure 2 could be mentioned here in addition to Extended Data Figures 6 and 7.

Lines 84-85. The definition of the median relative uncertainty could be mentioned here.

Line 87. close zero -> zero

Line 90. are shown in -> are explained in the Methods section and shown in

Line 120. Jx<0 within y<0, Jx>0 within y>0 -> Jx>0 within y<0, Jx<0 within y>0

Lines 147-148. Slices in ... measurements -> Slices of the Mercury's current systems in the X-Y plane at different Z values

Lines 153-155. I cannot find bolded black circles in Figures 3a and 3b.

Line 159. to -> at

Line 172. 21 -> 20

Lines 173-175. It could be difficult to separate J_IRC and J_MP since both could overlap without a gap.

Line 184. polar data -> data

Line 189. azimuth angle -> local time

Line 190. J_Phi>0 -> J_Phi<0

Lines 201-202. the magnetic induction equation in a steady-state case -> the Ohm's law

Line 219. middle latitude -> low altitude

Line 250. anisotropic -> isotropic

Lines 253-254. The phrase, if protons .. regions, could be deleted.

Lines 255-256. Why Figure 5 looks different from Figures 3a-c?

Lines 257-259. This may not be the case because ions would perform gradient B/curvature drift in the westward direction due to the Mercury's background magnetic field and this direction is opposite for electrons. However, the current J_IRC would be eastward after the magnetization current is added.

Lines 291-292. Magnetic field vectors are not shown in Figures 1b and 1c.

Lines 298-300. Which values of density and temperature were adopted?

Line 307. How characteristics of J_polar appear in simulation results?

Line 357. electron -> kinetic

Line 368. consider in investigating the dynamic -> the dynamics

Line 510. pectrometer -> spectrometer

Reviewer #3

(Remarks to the Author)
In their manuscript, "Global Current Systems in Mercury's Magnetosphere," Rentong Lin et al. analyze five years of magnetic field and plasma measurements obtained by the MESSENGER satellite. The authors propose a reconstruction of the global current system, comprising a magnetopause current, a cross-tail current, inner and outer equatorial ring currents, and a newly identified polar ring current. The authors argue that both the equatorial and polar ring currents are consistent with predictions of the plasma drifting model in regions where a pressure gradient takes place. These currents could reshape Mercury's magnetosphere, revealing new dynamics and energy transfer processes.

The experimental results presented here provide novel insights about the global current systems in Mercury's magnetosphere and warrant publication in Nature Communications. Nevertheless, the manuscript requires substantial revision.

Major points:

1)The introduction should be rewritten to ensure it is accessible to readers unfamiliar with Mercury's magnetosphere. It should provide a clear overview of what is currently known about Mercury's current system while highlighting the key questions that remain unanswered.

2)Consider integrating parts of the "Obtaining current using magnetometer data" section into the next section and moving the technical details to the Methods section.

3)Results for the global current system in Mercury's magnetosphere were obtained using Mercury Solar Orbital (MSO) coordinates as shown in Figures 2 and 3. However, this causes Jx color code to jump whenever the Y-coordinate flips sign.

A similar issue affects the Jy component, where sign changes in the X-coordinate obscure the actual direction of the current. This makes it challenging to determine whether the analyzed currents are flowing clockwise or counterclockwise. At the same time, Figure 4 displays the azimuthal component of the current vector, providing a much clearer and more intuitive visualization. I suggest the following improvements to the final stage of the analysis:

- Separate the analysis of the currents surrounding Mercury from the tail current.
- For the currents surrounding the Mercury, transform the Cartesian components (Jx, Jy, Jz) into spherical coordinate system. Cylindrical system might also work. You can still keep the MSO coordinates for the positions of current vectors, but replace the (Jx, Jy, Jz) color coding with values corresponding to spherical or cylindrical components. This will allow for a clear distingtion between clockwise and counterclockwise currents. This also makes the outer equatorial ring current easy to identify, as it is marked by a sign change in either the polar angle (latitude or colatitude) or the Z-component, depending on the chosen coordinate system.
- Combine the results shown in Figures 2a-c with those in Figure 3. Figures 2d-f can also be incorporated into this figure.
- For the tail current, the Cartesian components (Jx, Jy, Jz) can still be used (Figures 2 g-i), but they should be relocated to a new figure.

4)While Figure 1, showing the spatial distribution of the magnetic field's absolute value (|B|), provides useful information, it is entirely disconnected from the subsequent analysis. It would be more effective to remove Figure 1 and add the corresponding |B| slices as an additional column to the new figures described above. Comparing the magnetic field and currents might yield new insights.

5)The outer equatorial ring current proposed by the authors (lines 157-167) finds a direct analogue in the Earth's magnetosphere: the cut ring current, initially described by Antonova and Ganishkina (2000) and Antonova (2003, 2004). Comprehensive details regarding this current system can be found in Subsection 3.4 and Figure 5 of Ganushkina et al. (2017, Reviews of Geophysics, doi: 10.1002/2017RG000590), a paper cited by the authors as [35]. The explanation for this current proposes that it flows along a surface where the magnetic field strength reaches its minimum along a given field line. While the Earth's magnetic field is predominantly dipolar near the planet, solar wind compression on the dayside displaces these minimum-B locations away from the magnetic equator. This displacement causes the current to split into two distinct branches. This interpretation, grounded in fundamental concepts of plasma physics, offers a more robust and physically consistent explanation than the Shabansky orbit concept cited by the authors (lines 273-281). Please discuss the similarities between the outer equatorial ring current and the cut ring current and incorporate the corresponding references.

6)The authors state that “Ring currents generally result from opposite drift of ions and electrons in a region with pressure gradient” (lines 249-262). This attribution of the ring current to particle drift is incorrect, despite its prevalence in many space physics textbooks. In reality, the component of the current due to guiding-center drift is entirely canceled by a portion of the magnetization current. The remaining magnetization current, which is proportional to the plasma pressure gradient, is what generates the ring current. As clearly demonstrated in another review by Ganushkina et al. (“Defining and resolving current systems in geospace,” Ann. Geophys., doi:10.5194/angeo-33-1369-2015, Subsection 2.4, “Drifts and currents”), stationary transverse currents in geospace are generated by plasma pressure gradients. Consequently, changes in the ring current direction can be readily explained by the presence of a plasma pressure maximum, which leads to a reversal in the sign of the slope of the plasma pressure radial profile. This also will reinforce the explanation of the existence of the polar ring current.

7)The authors examined the influence of the solar wind on polar currents (Extended Data Fig. 3) by dividing the dataset according to heliospheric distance, finding no substantial differences. In my view, it is crucial to extend this analysis to other current systems. For instance, given that solar wind dynamic pressure compresses the dayside magnetosphere, one might expect an observable impact on the outer equatorial ring current.

Minor points:

lines 32-33
“.... its magnetic moment ranges from 190 to 223 nT·RM^3... “ Please, refrase to state clear that the model gives the values within the given range. Otherwise it gives a sensation that the magnetic moment could vary within the indicated range. Details about the Mercury’s magnetic moment can be moved to data analysis part.

lines 64-66
This paragraph is more suitable for the introduction

line 67
A few words about the Messenger mission are needed before the description of the magnetometer, including the orbit.

line 187
Please, check capture in Figure 4. Looking from the Northern pole, the predominant direction of the current is clockwise, that correspond to -JΦ, i.e. the azimuthal component is directed contrary to the azimuthal basic vector. This agrees with the color scale on the right side of the graph, but contracts to the definition “JΦ > 0 represents clockwise and JΦ < 0 represents anti-clockwise”.

line 590
Extended Data Fig. 5(a) The vertical axis should be Y. This figure is important, and I recommend to move it to the core part of

the paper.

With best wishes

Marina Stepanova

Version 1:

Reviewer comments:

Reviewer #1

(Remarks to the Author)
The authors greatly improved the study and made it clearer and physically justified. They have took into account all comments and suggestions, changed the text and figures and added additional figures for clarification of results. There is a minor comment: at lines 87, 160 – "…provided in the Method section …". But the section is called Methods. After this minor correction the paper can be accepted to the journal.

Reviewer #2

(Remarks to the Author)
Each type of ring current is easier to be identified after the data are plotted in the cylindrical coordinates. The explanation in the manuscript has been clearer after the revision. Nevertheless, I have one comment. The current is related to the pressure gradient through the relation: JxB=grad P. The dayside portion of the polar ring current is related to the pressure gradient in the cusp as can
be seen in the Extended Data Figure 11. However, there is not significant pressure variation near the location of the nightside polar ring current. The authors could explain that reason.

Specific comments

Figure 3. (d) view from -Y -> (d) view from +Y. A similar correction may be made for panel e.

Line 157. direction of counter-clockwise -> the counter-clockwise direction

Line 161. Is rho defined previously?

Line 175. The confinement of the polar ring current to high latitudes or the separation between the polar ring current and the outer ring current would be clearer if the current distribution in the X-Y plane at Z=0.30-0.75 RM or those with finer Z resolution are shown.

Line 188. The polar ring current seems to be stronger at the nightside when referring to blue colors near the grey circle in Figure 4b.

Line 213. latitude -> altitude

Line 258. reversal -> reversed

Line 274. Each column of panels is colored by the components ... coordinates, respectively. -> Panel b is colored by the azimuthal component ... coordinates.

Reviewer #3

(Remarks to the Author)
The authors have successfully addressed the reviewers' suggestions, significantly strengthening the manuscript. The results obtained clearly warrant publication in Nature Communications. However, the manuscript is still challenging to follow in places. I therefore recommend a full editorial revision focused on enhancing the overall style, clarity, and fluency. While I might not typically raise this issue for publication in a specialized journal, Nature Communications is aimed at a broader audience. Therefore, the manuscript needs to be exceptionally clear to ensure a strong scientific impact on the wider scientific community. Next, I will present two paragraphs solely to illustrate my point.

lines 34-39
Mercury has an intrinsic dipole magnetic field with a northward offset (1, 2, 3). Mercury's intrinsic magnetic field interacts with the solar wind and forms a magnetosphere, which was discovered by Mariner-10 in the 1970s (4) and has been the target of the NASA space mission MESSENGER (5) and of the upcoming ESA-JAXA BepiColombo mission (6). The Mercury's magnetosphere is highly compressed on the dayside by the conducting solar wind due to Mercury's proximity to the Sun. ---->

Mercury has an intrinsic dipole magnetic field characterized by a northward offset (1, 2, 3). Its interaction with the solar wind

forms a magnetosphere, first discovered by Mariner 10 (4) and subsequently studied by NASA's MESSENGER (5) and the ESA-JAXA BepiColombo missions (6).
Due to the planet's proximity to the Sun, Mercury's magnetosphere is highly compressed on the dayside by the dense solar wind.

lines 58-62
The magnetospheric model of Mercury's magnetosphere by Pump et al. (15) further verified the existence of the eastward ring current. The westward ring current is validated by test-particle simulation (9) and the eastward current is well reproduced by hybrid simulations (10) and the magnetospheric model (15). ---->

While the westward ring current has been validated by test-particle simulations (9), the eastward current is well supported by hybrid simulations (10) and the magnetospheric model of Pump et al. (15).

Version 2:

Reviewer comments:

Reviewer #2

(Remarks to the Author)
The authors considered my previous comments. I do not have further comments.

Open Access This Peer Review File is licensed under a Creative Commons Attribution 4.0 International License, which permits use, sharing, adaptation, distribution and reproduction in any medium or format, as long as you give appropriate credit to the original author(s) and the source, provide a link to the Creative Commons license, and indicate if changes were made.
In cases where reviewers are anonymous, credit should be given to 'Anonymous Referee' and the source.
The images or other third party material in this Peer Review File are included in the article's Creative Commons license, unless indicated otherwise in a credit line to the material. If material is not included in the article's Creative Commons license and your intended use is not permitted by statutory regulation or exceeds the permitted use, you will need to obtain permission directly from the copyright holder.
To view a copy of this license, visit https://creativecommons.org/licenses/by/4.0/

Reply to the Reviewers

We greatly thank the reviewers for the valuable comments/suggestions that we considered in the resubmitted manuscript. All revised parts are marked in red in the text. Detailed answers to the comments are given below:

--

Reply to Reviewer #1:

This paper uses magnetic field measurements obtained by MESSENGER spacecraft to describe the Hermean magnetospheric current systems. Authors used the curlometer technique to find current densities along MESSENGER orbits. This technique is applied to MESSENGER mission data for the first time, thus the obtained results are rather novel, for example the polar ring current existence and two different equatorial ring currents existence have been shown. Nevertheless there are issues to be clarified. The curlometer technique was initially developed to analyze the multi-spacecraft measurements (Dunlop et al., 2021, 10.1029/2021JA029538). Using the single-spacecraft data should be carried with even more than usual caution, because there are many factors which can lead to a change of magnetic field, which can be interpreted as an electric current using this method.

The following are the comments and questions about the manuscript:

1. Why magnetospheric current systems are analyzed in MSO (Mercury Solar Orbiter) coordinate system, but not in MSM (Mercury Solar-Magnetospheric) coordinate system?
Thanks for the comment. The current density is derived from spatial derivatives of the magnetic field. Because the MSM coordinates differs from the MSO coordinates only by a constant translation of the origin along the z direction (with parallel basis vectors), this translation does not affect the computed spatial gradients or the resulting current density. We therefore use MSO for consistency with the native coordinate system of the MESSENGER magnetic-field data.

2. A grid spacing of 0.15 RM is used in the study, which has been chosen from consideration of 0.1 RM and 0.2 RM. But, the Hermean magnetosphere is very dynamic (Slavin et al., 2010). Thus on a timescale of minutes magnetospheric state can change significantly. What was the distance covered by MESSENGER on a timescale of minutes? If it is less than 0.15 RM, then the change of magnetic field could come from the reconfiguration of the magnetosphere. Also, what is the size of the gyro-radius for the described spatial regions comparing to a grid spacing? Slavin et al. (2008, 10.1126/science.1159040) mentioned that for example double magnetopause signature has been observed at Mercury and "for Na+, the depth of penetration into the magnetosphere would be ~1 gyro-radius or ~1000 km", which can influence the observations. Thus, the choice of 0.1, 0.15 and 0.2 RM grid spacing values should be justified better.
Thanks for this important comment. We agree that the choice of grid spacing requires careful justification, especially in Mercury's highly dynamic magnetosphere.

Firstly, the MESSENGER orbital velocity ranges from approximately 0.5 to 4 km/s, corresponding to a travel distance of about 30–240 km (roughly 0.01–0.10 R_M) in one minute. We note, however,

that our method does not infer current density from temporal variations measured along a single pass. Instead, we first construct a multi-orbit, time-averaged three-dimensional magnetic-field map and then apply the first-order central finite difference method to that averaged magnetic field. Therefore, the key issue is not the spacecraft travel distance itself, but whether the multi-orbit averaging recovers a sufficiently robust large-scale quasi-steady magnetic configuration.

Secondly, we examined the characteristic ion kinetic scales in the relevant regions. As shown in the following Figure A1, the proton gyroradius is of the order of ~100 km in the magnetopause region, generally less than 100 km in the $\boldsymbol{J}_{\mathrm{IRC}}$ region, and typically less than 10 km in the $\boldsymbol{J}_{\mathrm{polar}}$ region. These scales are smaller than, or at most comparable to, the adopted grid spacing of 0.15 R_M, indicating that the main current structures discussed here are resolved at a scale appropriate for a statistical study of large-scale current morphology. We acknowledge that heavy ions such as Na^+ may have larger gyroradii and can affect local structures, but our reconstruction aims at the global, quasi-steady current systems rather than kinetic-scale or transient features.

Thirdly, we compared reconstructions using grid spacings of 0.10, 0.15, and 0.20 R_M. We find that the large-scale current morphology is broadly consistent between 0.15 and 0.20 R_M, whereas at 0.10 R_M the number of valid grid cells after quality control decreases substantially and the reconstructed current pattern becomes noticeably more fragmented. We therefore selected 0.15 R_M as a compromise between spatial resolution and statistical robustness. We have clarified this point in the revised manuscript and retained the 0.10 and 0.20 R_M results in the Extended Data for comparison.

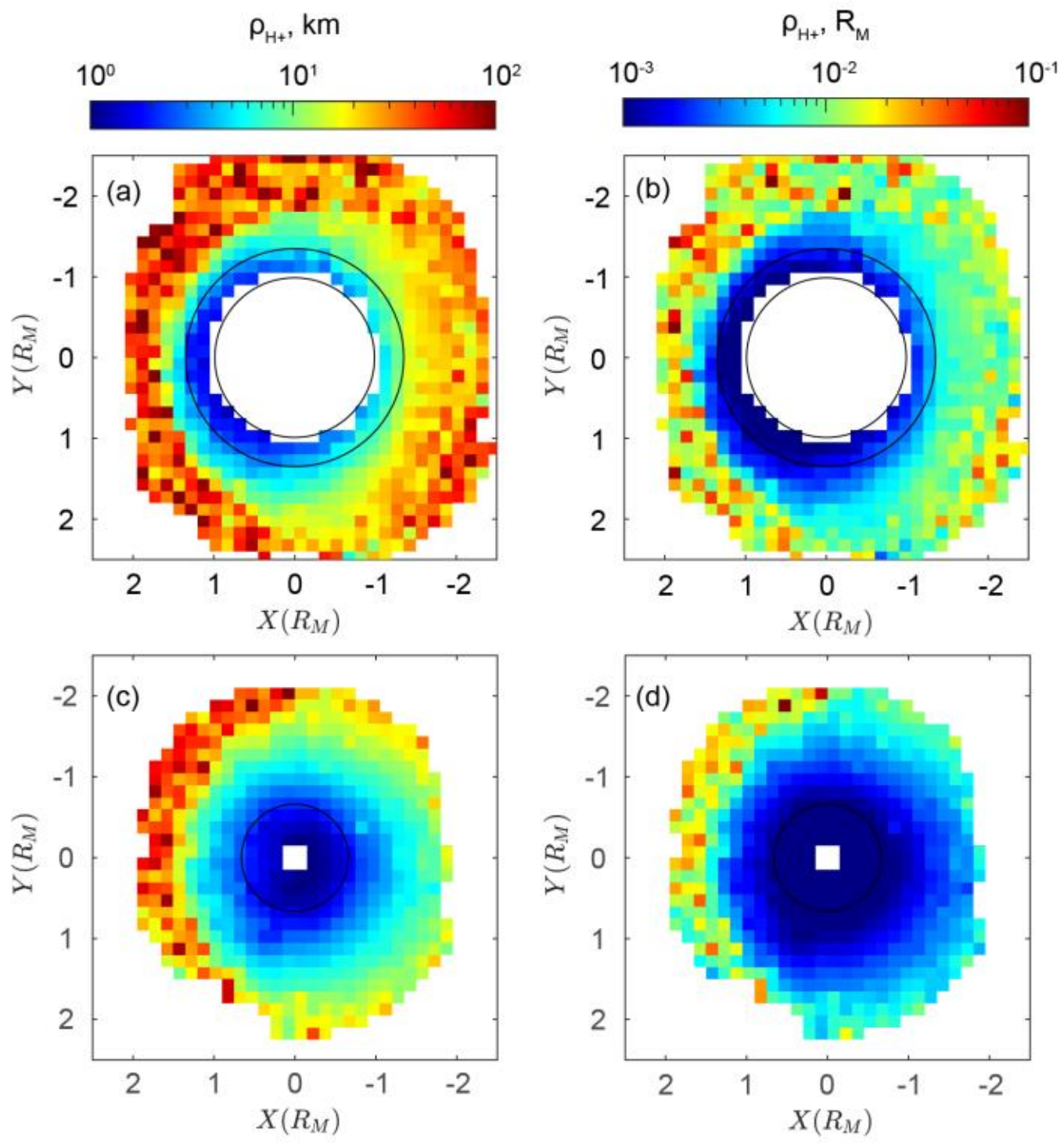

Figure A1. Gyroradius of protons in X-Y plane within regions of J_{ORC} (a-b) and J_{polar} (c-d).

In the revised manuscript, we also clarify that the present study is intended to recover the large-scale quasi-steady current systems of Mercury's magnetosphere, rather than short-timescale transient

current structures.

3. As it has been mentioned, Hermean magnetosphere is highly dynamic. The subsolar magnetopause distance can change significantly (see for example Zhong et al., 2015, 10.1002/2015GL067063). Sometimes it can almost disappear (Slavin et al., 2019, 10.1029/2019JA026892). Thus at the same point at MSO coordinate system for different spacecraft orbits there may be very different conditions. This should be mentioned in the text. But at this manuscript all measurements are studied at the same time and are compared with a fixed magnetopause surface. Does it influence the obtained results?

Thank you for raising this important point. We agree that Mercury's magnetosphere is highly dynamic, and that the spacecraft can indeed sample very different plasma and magnetic-field conditions at the same MSO location during different orbital passes. We have now stated this more explicitly in the revised manuscript.

Our approach is statistical: measurements from many orbital passes are binned into spatial grids to reconstruct the average magnetic-field configuration and its associated large-scale current systems. In this framework, short-term variability in the magnetopause position and in the magnetospheric configuration contributes to the spread of measurements within each grid cell and increases the uncertainty of the reconstructed current density. This variability is therefore a source of uncertainty, but it does not invalidate the objective of the study, which is to recover the quasi-steady large-scale current morphology rather than instantaneous current structures.

To assess whether the multi-orbit averaging still yields a meaningful large-scale field model, we examined both the spread of the magnetic-field samples within grid cells and the numerical self-consistency of the reconstructed magnetic field. The corresponding distributions are shown in Figures A2 and A3 (Extended Data Figures 15 and 16 in the revised manuscript). Figure A2 shows that the dispersion/uncertainty of the gridded magnetic-field and current-density estimates remains moderate for most valid grid cells, and Figure A3 shows that the ratio $|\nabla\cdot B|/|\nabla\times B|$ is generally small in the regions of interest. These diagnostics indicate that the averaged magnetic-field model is sufficiently self-consistent for resolving the large-scale current morphology discussed here, although local uncertainties remain significant in sparsely sampled regions.

In our study, only results with $|\nabla\cdot B|/|\nabla\times B| < 0.5$ were retained. Moreover, both the magnetopause and magnetotail are highly dynamic. The magnetopause current $\boldsymbol{J}_{MP}$ and the cross-tail current $\boldsymbol{J}_{CT}$ can be clearly identified from our results, which further supports the reliability of our calculation method and the obtained results.

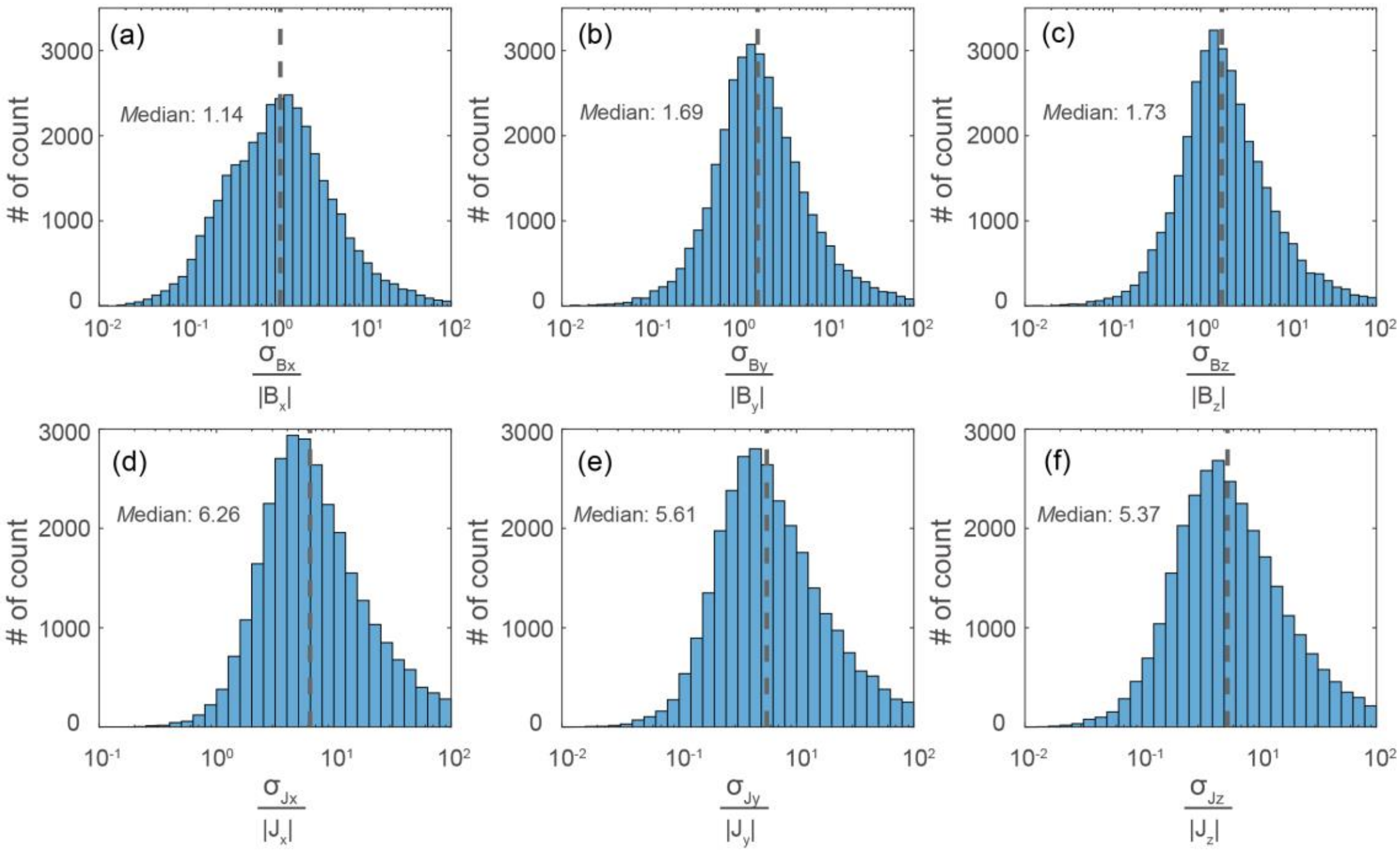

Figure A2. Distribution of relative errors. Distribution of relative errors of each magnetic field components (a-c) and current density components (d-f). Dashed lines represent the median value.

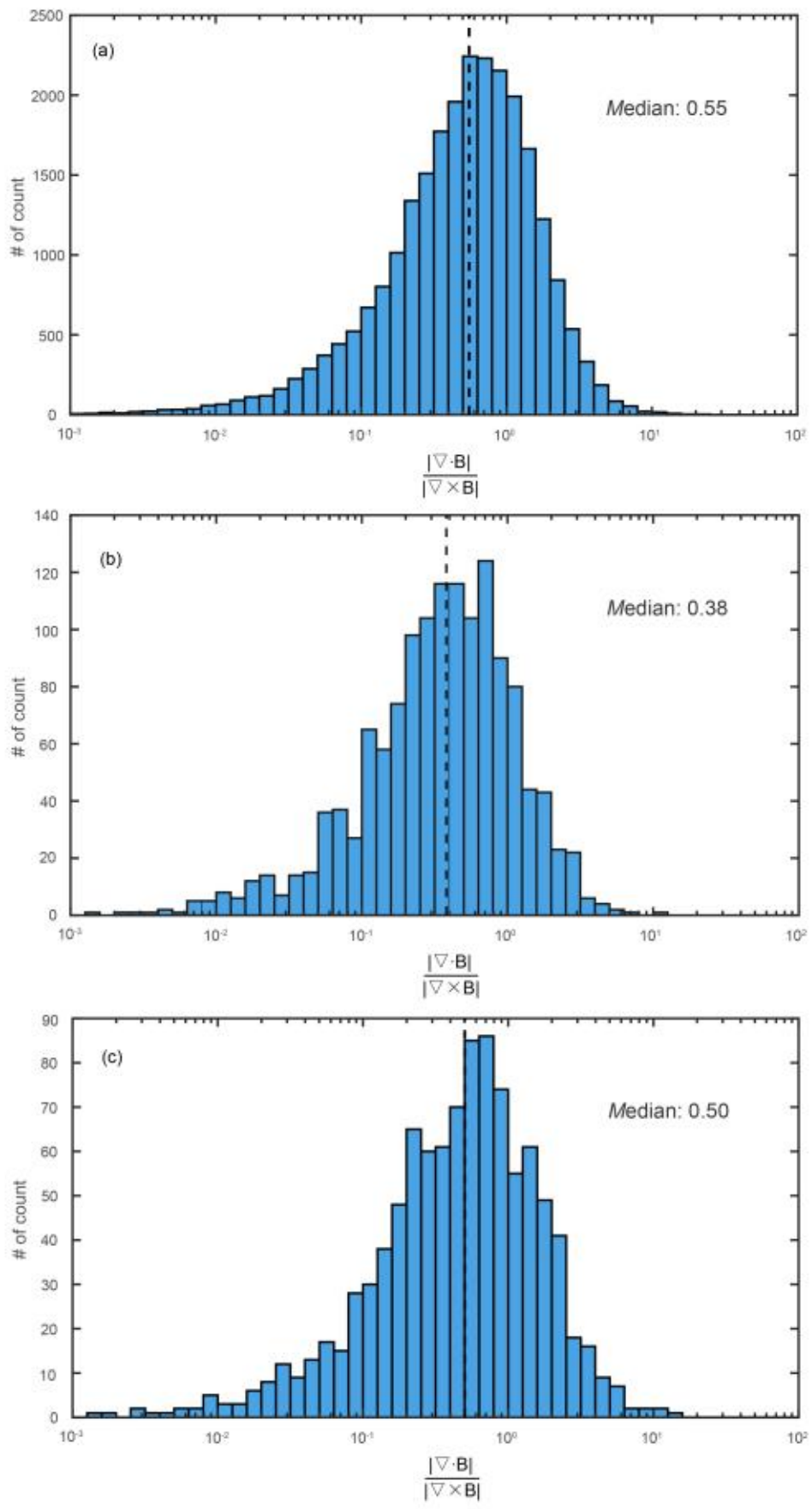

Figure A3. Distributions of relative deviation from Gauss' law for the magnetic field divergence.

The relative divergence (a) including all grid cells, (b) within 0.15 $R_M \leq z \leq 0.30\ R_M$, and (c) within 0.75 $R_M \leq z \leq 1.20\ R_M$. The dashed lines represent the median.

To further assess the influence of changing solar-wind forcing and orbital sampling bias, we divided the dataset into two subsets according to heliocentric distance and repeated the current reconstruction separately. The new results are shown in Figures A4 – A7 (Extended Data Figures 6-9 in the revised manuscript). We find that the magnetopause current and cross-tail current become stronger, and that the magnetopause is located closer to Mercury, when Mercury is nearer the Sun. These trends are physically consistent with a more compressed magnetosphere under stronger solar-wind dynamic pressure. We also find that the $\boldsymbol{J}_{\mathrm{IRC}}$ is much more clearly resolved at larger heliocentric distance, while the high-latitude azimuthal current associated with $\boldsymbol{J}_{\mathrm{polar}}$ remains detectable in both subsets. These sensitivity tests support the robustness of the main large-scale current patterns reported in this study, while also illustrating how magnetospheric compression affects their visibility.

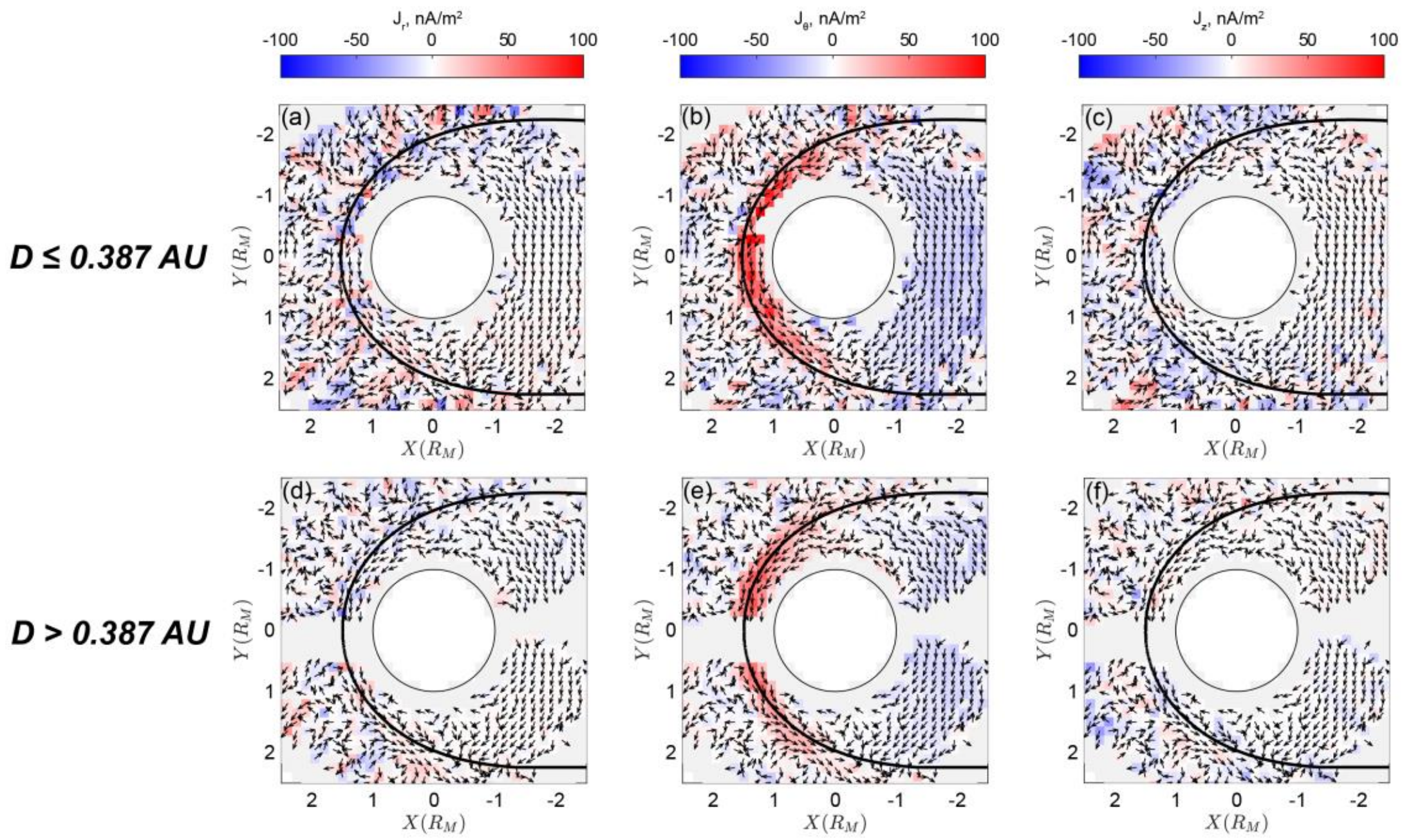

Figure A4. Magnetopause current at different heliocentric distances.

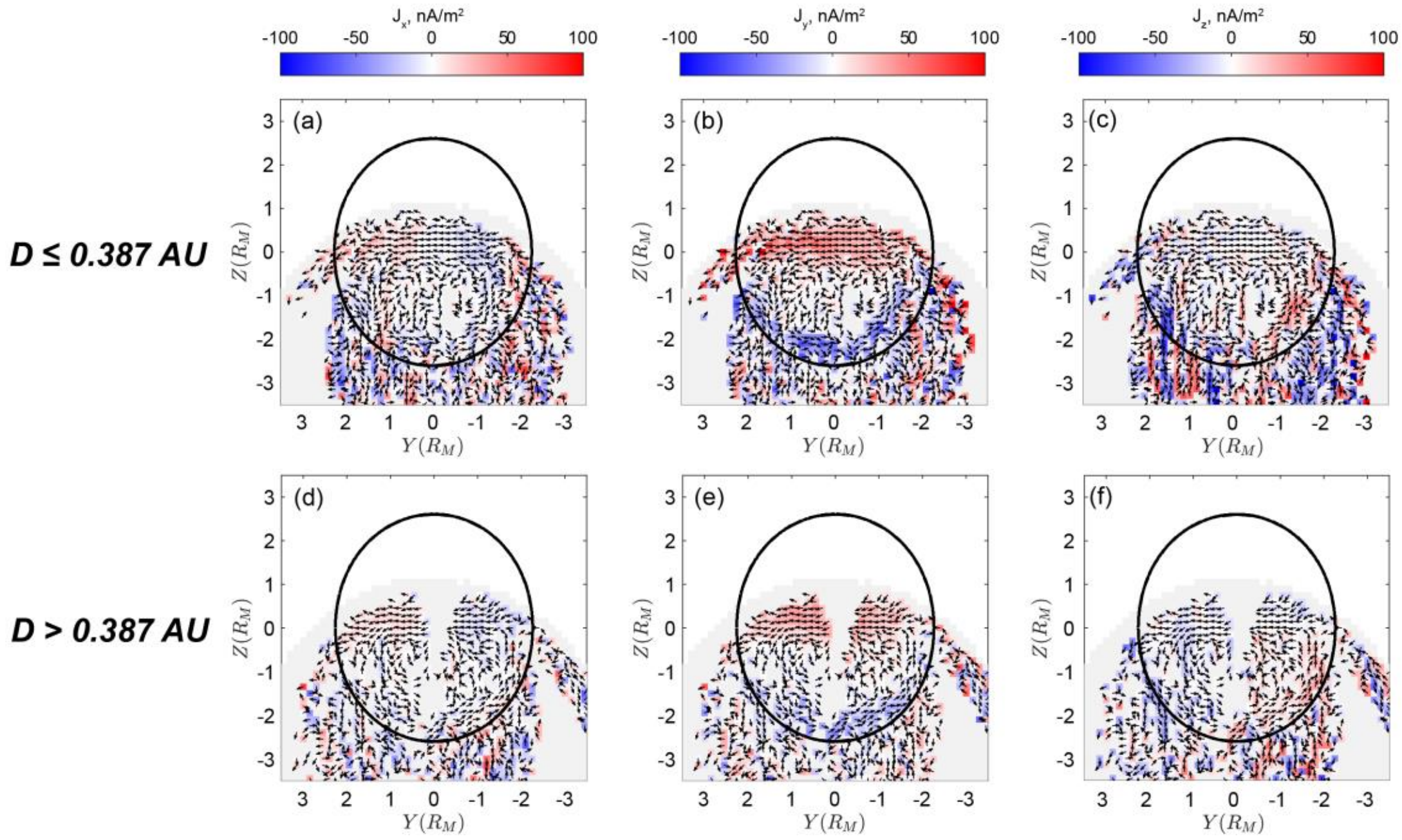

Figure A5. Cross-tail current at different heliocentric distances.

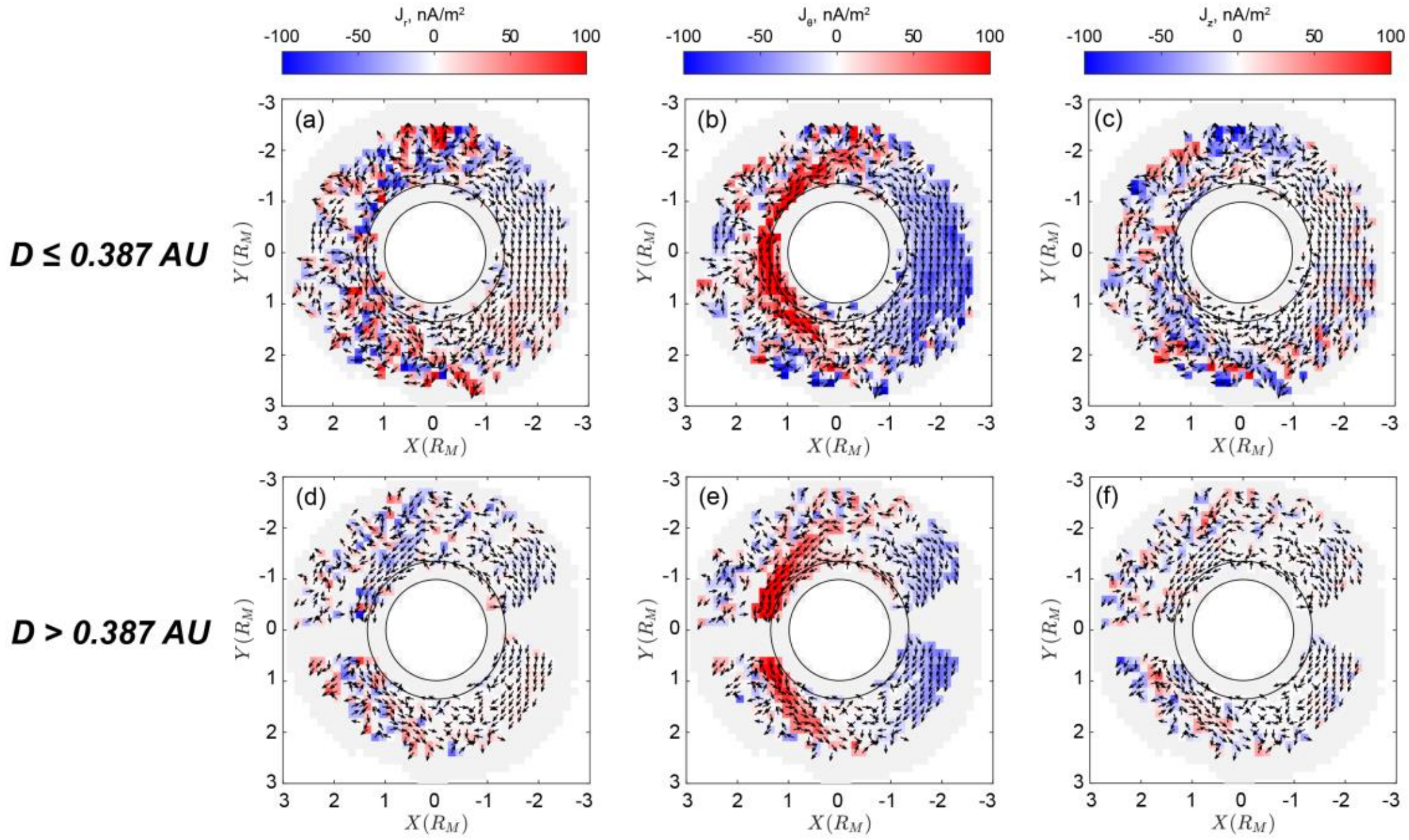

Figure A6. Equatorial ring current at different heliocentric distances.

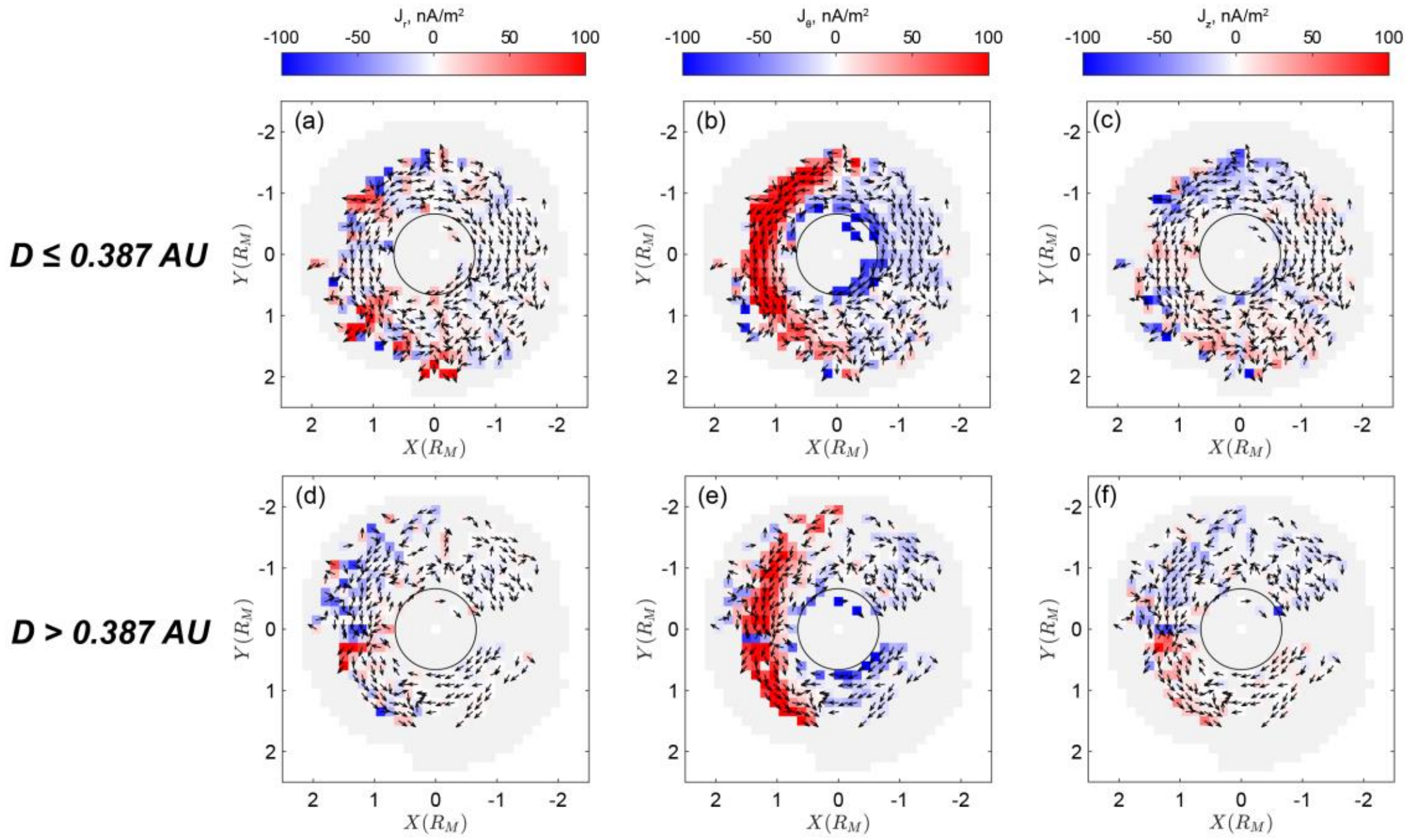

Figure A7. Polar ring current at different heliocentric distances.

We have added a brief discussion of these points to the main text and included the corresponding figures in the Extended Data. We also clarify in the revised manuscript that the empirical magnetopause shown in the figures is used as a reference boundary for interpretation, not as an assumption that the magnetopause is fixed in time.

4. The main findings are made from the figures with current densities maps, but the detected currents are not indicated in any way in the figures. For example, for J_orc: "As shown in Figure 3a & 3b, there exists a clockwise current in the near-Mercury magnetotail (-1.4 RM < x < 0)". But on the figure the curved tail currents are obviously seen, like on figure 3 at Ganushkina et al. (2018, 10.1002/2017RG000590). Please, highlight the areas which you consider for J_orc. The same could be done for J_irc, although it is more visible. Also, at Figure 4, the shown arrows in the nightside are located much above the equatorial plane. Please, show the magnetic equatorial plane at figure 4 to make it more clear, that J orc is located close to magnetic equator in the nightside.

Thanks for this valuable suggestion. We agree that the identified current systems should be indicated explicitly in the figures. In the revised figure set, we highlighted the regions used to identify the $\boldsymbol{J}_{\mathrm{ORC}}$ and the $\boldsymbol{J}_{\mathrm{IRC}}$ in the relevant 2-D slices, and we also marked the magnetic equatorial plane in the 3-D view. These changes make it clearer that the nightside portion of $\boldsymbol{J}_{\mathrm{ORC}}$ lies close to the magnetic equator, while its dayside branch extends to higher latitude. We revised the figure captions and the corresponding text accordingly. In particular, the revised equatorial-current figure now combines the planar view and the 3-D view to show both the ring-current morphology in the equatorial plane and the off-equatorial continuation on the dayside (Figure A8 / Fig. 3 in the revised manuscript).

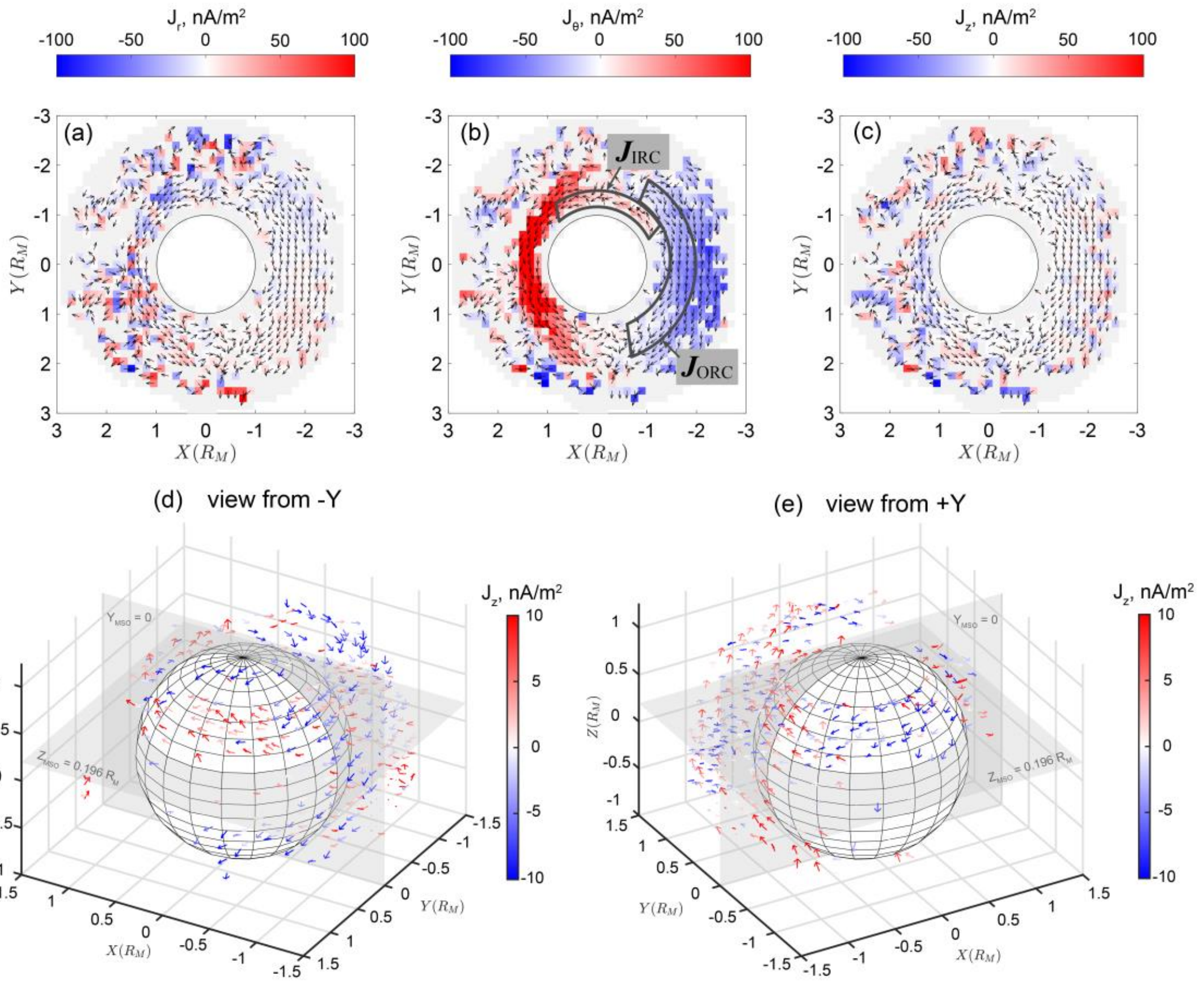

Figure A8. Slices in the X-Y plane and side view of the three-dimensional map of the Mercury's current systems. Panels a-c show a slice in the X-Y plane within $0.15\ R_M \le z \le 0.30\ R_M$. In panel d-e, the axis range is limited to $|x| < 1.5\ R_M$, $|y| < 1.5\ R_M$ and $|z| < 1.25\ R_M$. Each column of panels is colored by the components of the current density in cylindrical coordinates, respectively. Arrows in panels a-c show the current direction in the projected plane of each panel. The black circles in panel a-c and the sphere in panel d-e represents the Mercury. The grid cells in grey represent no valid measurements. The regions used to identify $\boldsymbol{J}_{ORC}$ and $\boldsymbol{J}_{IRC}$ are indicated by the grey enclosed areas in panel b. The magnetic equatorial plane for reference in panels d-e is indicated by the translucent surface ($Z = 0.196\ R_M$). Arrows represent the current density vector. To present the $\boldsymbol{J}_{ORC}$ distinctly, arrows with $J_\theta > 0$ are not shown. Color of arrows in panels d-f represents the z-component of the current density in cylindrical coordinates.

5. Slices for figures 2a-f and 3a-c have a reasonable size of 0.15 RM, which is a grid step. The slice for J_polar has a size of 0.45 RM (0.75 RM ⩽ z ⩽ 1.20 RM). It is three times larger than the grid step. Please, explain the choice of this range. J_polar should be highlighted as well and maybe it is reasonable to show it in a narrower slice to describe its actual location.

Thank you for this comment. We agree that the choice of the high-latitude slice should be explained more clearly. The current density is still calculated on the 0.15 R_M grid. For visualization of the polar current, however, we combine several adjacent z-layers into a thicker slice (0.75 – 1.20 R_M) because the polar region contains fewer valid grid cells after quality control than the equatorial region. This improves the visibility of the large-scale azimuthal current pattern without changing the underlying grid used in the current calculation.

In the revised figures, we also explicitly highlight the region identified as $\boldsymbol{J}_{polar}$. In addition, we provide narrower-slice (Figure A9) and sensitivity-test results (Figure A10) to show that the same high-latitude azimuthal current signature is present, although it becomes less continuous and statistically less significant when fewer valid grid cells are available.

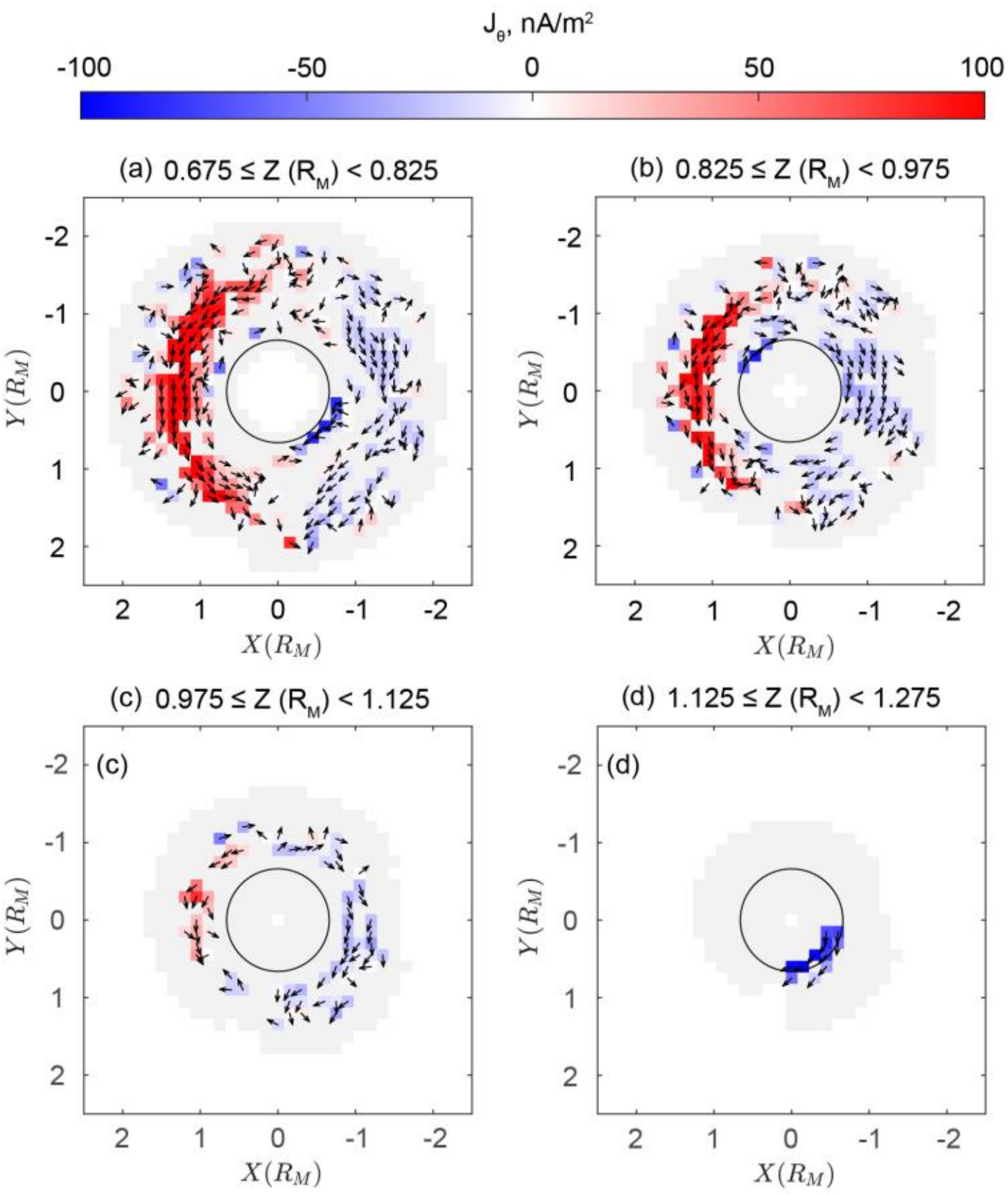

Figure A9. Current density in size of 0.15 R_M within different range of Z in MSO coordinates.

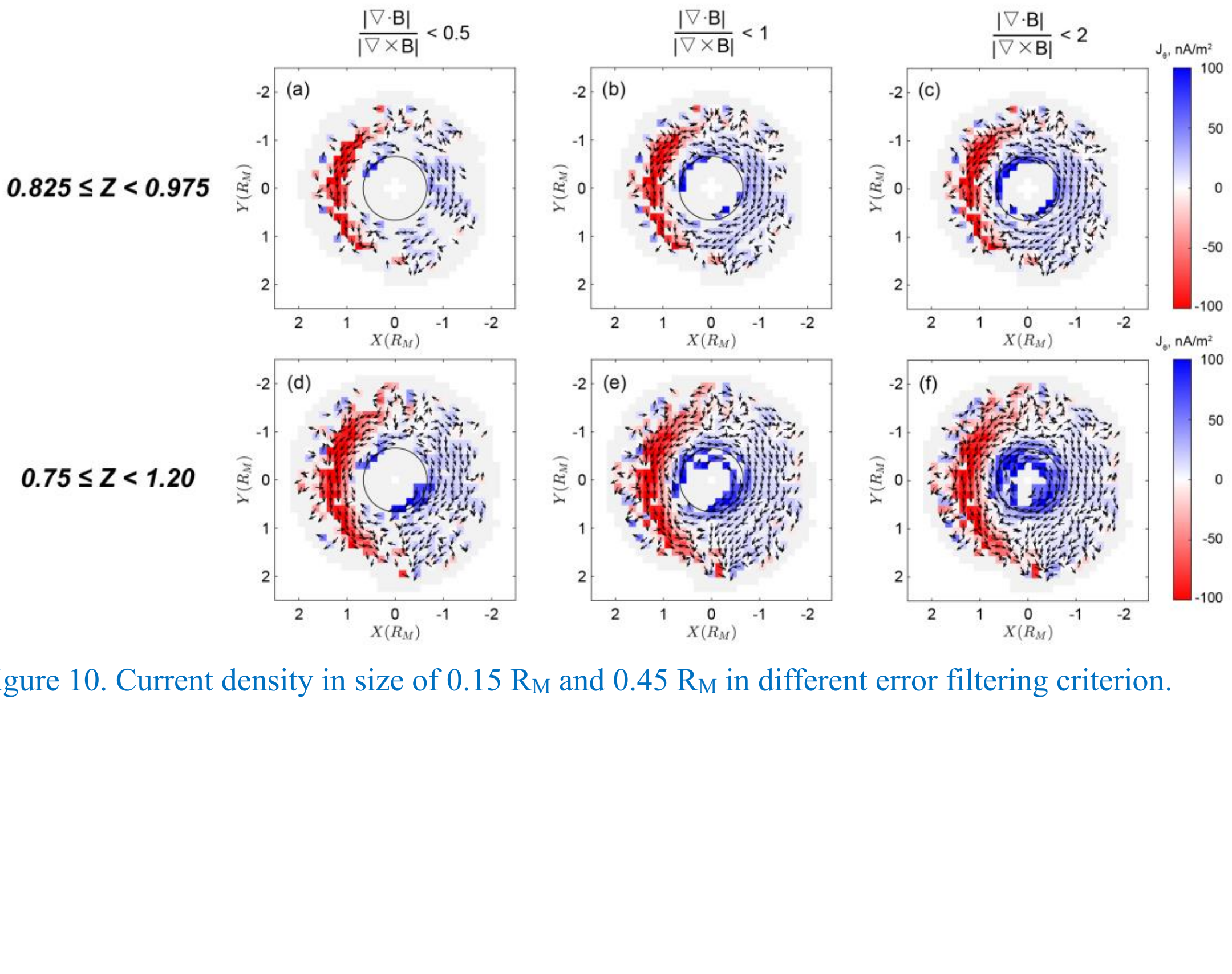

Figure 10. Current density in size of 0.15 R_M and 0.45 R_M in different error filtering criterion.

6. Figures 5 and extended 5 are very important in describing the nature of J_irc and J_orc. Please highlight the IRC and ORC at the figure 5.

Thanks for this suggestion. In the revised figure, we explicitly mark the regions corresponding to $\boldsymbol{J}_{\mathrm{IRC}}$ and $\boldsymbol{J}_{\mathrm{ORC}}$ so that the plasma-pressure-gradient-based current estimate can be compared more directly with the current systems reconstructed from the magnetic field. Because the plasma-pressure distribution is central to the physical interpretation of the ring-current-like structures, we also moved the former Extended Data Figure 5 into the main text.

7. J_irc looks similar to eastward current found by Shi et al. (2022, 10.1029/2022GL098415). Please, specify it in 'Physical nature of the current systems' section.

Thanks for this comment. We expanded the discussion in the "Physical nature of the current systems" section to clarify the relationship between $\boldsymbol{J}_{\mathrm{IRC}}$ and the eastward current reported by Shi et al. (2022). In particular, we now note that both currents share a similar eastward direction and comparable location on the planetward side of the near-tail pressure peak.

8. Poh et al. (2017, 10.1002/2016GL071612) have shown that the inner current sheet edge location at Mercury is located at x = −1.22 RM. The location of plasma pressure peak in the manuscript should be compared with this value. If they are comparable, does it mean that J_orc, which is located further from the planet from this point and J_ct are located inside each other in the nightside?

Thanks for this insightful comment. In our midnight-sector pressure distribution, the proton pressure maximum is located at a radial distance of about 1.15–1.25 R_M from Mercury's center, which is broadly comparable to the inner current-sheet-edge location reported by Poh et al. (2017). We note, however, that the true pressure peak may lie somewhat closer to the planet because MESSENGER did not sample lower altitudes in this region.

This comparison suggests that the nightside $\boldsymbol{J}_{\mathrm{ORC}}$ may indeed lie planetward of, and partially adjacent to, the main cross-tail current region, so that the two current systems can appear spatially nested in an average sense. We nevertheless interpret them as physically distinct: $\boldsymbol{J}_{\mathrm{CT}}$ is the principal tail current-sheet current, whereas $\boldsymbol{J}_{\mathrm{ORC}}$ is more naturally explained as a pressure-gradient-driven ring/current-loop structure closer to Mercury. We clarified this point in the revised manuscript and avoided overinterpreting their dynamical relationship.

We sincerely appreciate your insightful comments and constructive suggestions. They have greatly improved the rigor, clarity, and physical interpretation of our study.

Reply to Reviewer #2:

The authors analyzed five years of MESSENGER spacecraft data to derive a three-dimensional pattern of Mercury's magnetic fields. The global current system was then calculated by the first-order central finite difference method in the polar region as well as in the equatorial region. Various currents were identified: magnetopause current, cross-tail current, inner and outer equatorial ring

current, and polar ring current. The polar ring current was discovered by the authors. If these authors' claim would be robust, their finding would advance understanding dynamics of the Mercury's magnetosphere.

However, there are unclear parts in the authors' analysis. The first one is identification of the polar ring current. In Figures 3d and 3e, the polar ring current appears at prenoon and premidnight local time near the Mercury's surface depicted with thin black circles. The current does not constitute a circular shape which is usually the case for the ring current. The authors divided the data set referring to the Mercury's heliocentric distance D and performed the same statistics (Extended Data Figure 3). There is some difference between the distribution of the current in Figures 3d and 3e and those in Extended Data Figure 3, particularly at prenoon. Therefore, the statistical significance of the polar ring current would not be large enough.
Thanks for this important comment. We agree that the incomplete annular appearance in the original Cartesian plots makes the identification of $\boldsymbol{J}_{\mathrm{polar}}$ less straightforward. In the revised manuscript, we therefore rely primarily on the cylindrical-coordinate representation, in which the dominant azimuthal component of the high-latitude current is much clearer even under our original strict quality-control criterion.

We further note that the stricter filtering preferentially removes some high-latitude grid cells because of limited sampling coverage, which makes the current pattern appear discontinuous in the original plots. When the filtering criterion is relaxed, the annular morphology becomes more continuous (Figure B1), but we use this result only as a sensitivity test rather than as the primary evidence for $\boldsymbol{J}_{\mathrm{polar}}$. In addition, the high-latitude azimuthal current remains detectable in the heliocentric-distance subsets, which further supports the robustness of this feature.

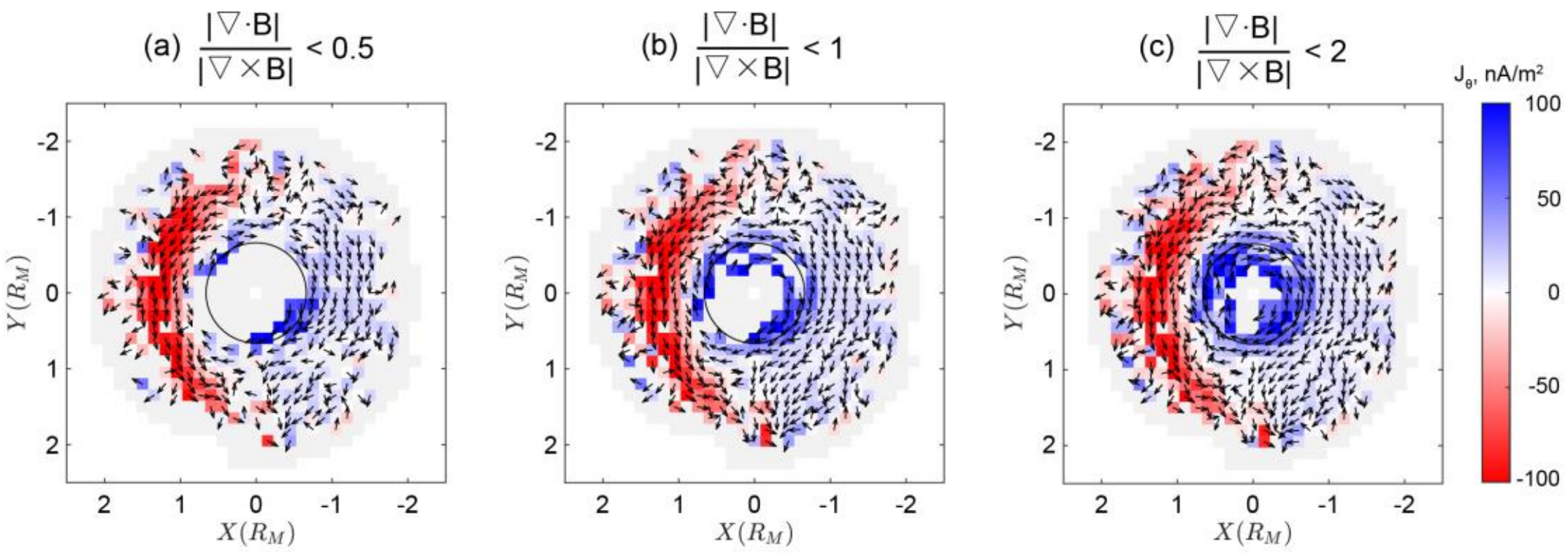

Figure B1. Current density of the $\boldsymbol{J}_{\mathrm{polar}}$ in different error filtering criterion.

The second part is interpretation of the polar ring current in terms of pressure gradient in the cusp region. The authors related these two quantities. However, the derived location of the polar ring current at prenoon and premidnight in Figures 3d and 3e or the anticipated location of the current at the whole local time does not match with that of the cusp, which is prenoon as well as postnoon (Sun et al., 2022; Winslow et al., 2012; both referred in the manuscript). Therefore, the mechanism suggested by the authors would not work, depending on local time.
Thank you for your comment. As noted in the previous response, in the cylindrical coordinates the

dominant azimuthal component of the high-latitude current is much clearer even under our original strict quality-control criterion. And when the filtering criterion is relaxed, the annular morphology becomes more continuous (Figure B1).

The equatorial ring current is primarily driven by sunward-moving high-speed plasmas following magnetotail magnetic reconnection, which is consistent with the pressure gradient in the magnetotail. We suggest that the polar ring current is formed by a similar mechanism, except that it is driven by solar wind protons entering from the dayside cusp, which is consistent with the pressure gradient at the dayside cusp (Figure B2 / Extended data Fig. 11 in the revised manuscript).

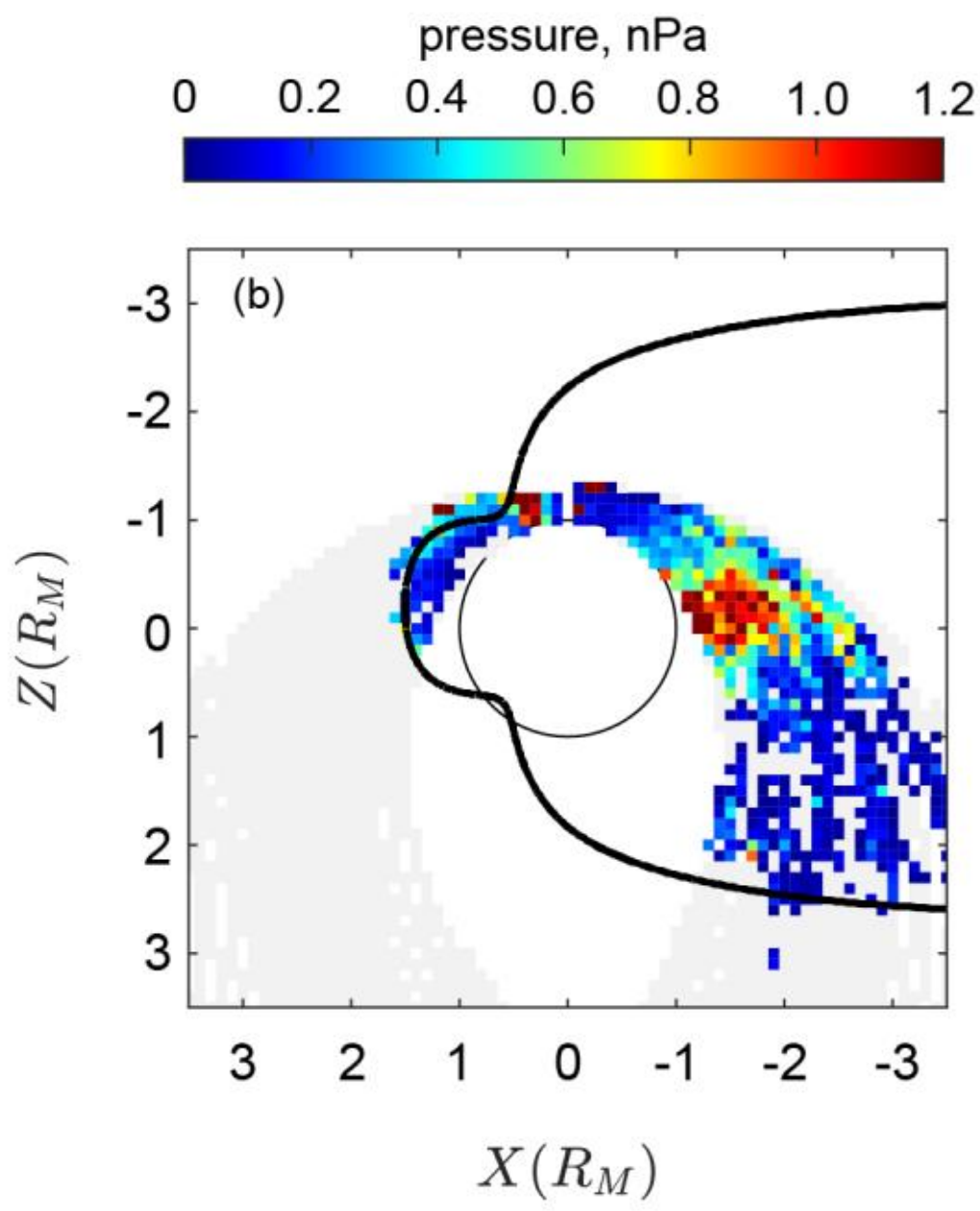

Figure B2. Distribution of averaged pressure of H^+ ions in X-Z plane at Y=0. Distribution of plasma pressure derived from the Fast Image Plasma Spectrometer (FIPS) measurements. The grid cells in grey represent no valid measurements.

The third part is the relation between the outer equatorial ring current and the polar ring current (Figure 6). Even though both flow off the equator in the dayside, they were treated as different current. I wonder whether both would be the same, if these currents would really exist. The authors identified the outer equatorial ring current from Figure 4. However, it is hard to identify current from this figure because the plot is a three-dimensional map. Moreover, the upward current in the duskside and the downward current in the dawnside, part of the outer equatorial ring current, cannot be identified in Figure 3.

Thanks for this comment. We agree that the distinction between $\boldsymbol{J}_{\mathrm{ORC}}$ and $\boldsymbol{J}_{\mathrm{polar}}$ should be demonstrated more clearly in the figures. In the revised manuscript, we combined the planar and 3-D views of the equatorial current system into a new figure (Figure B3), which makes the duskward upward branch and dawnward downward branch of $\boldsymbol{J}_{\mathrm{ORC}}$ much easier to identify.

Our interpretation that $\boldsymbol{J}_{\mathrm{ORC}}$ and Jpolar are distinct is based primarily on their different spatial locations and morphologies: $\boldsymbol{J}_{\mathrm{ORC}}$ is the off-equatorial continuation of the equatorial ring-current-

like system, whereas $\boldsymbol{J}_{\mathrm{polar}}$ is a separate high-latitude azimuthal current concentrated around the northern cusp/polar region. The two systems may share the same general pressure-gradient-driven ring-current physics, but the present reconstruction suggests that they occupy different regions and should be treated as distinct current systems in the large-scale average morphology.

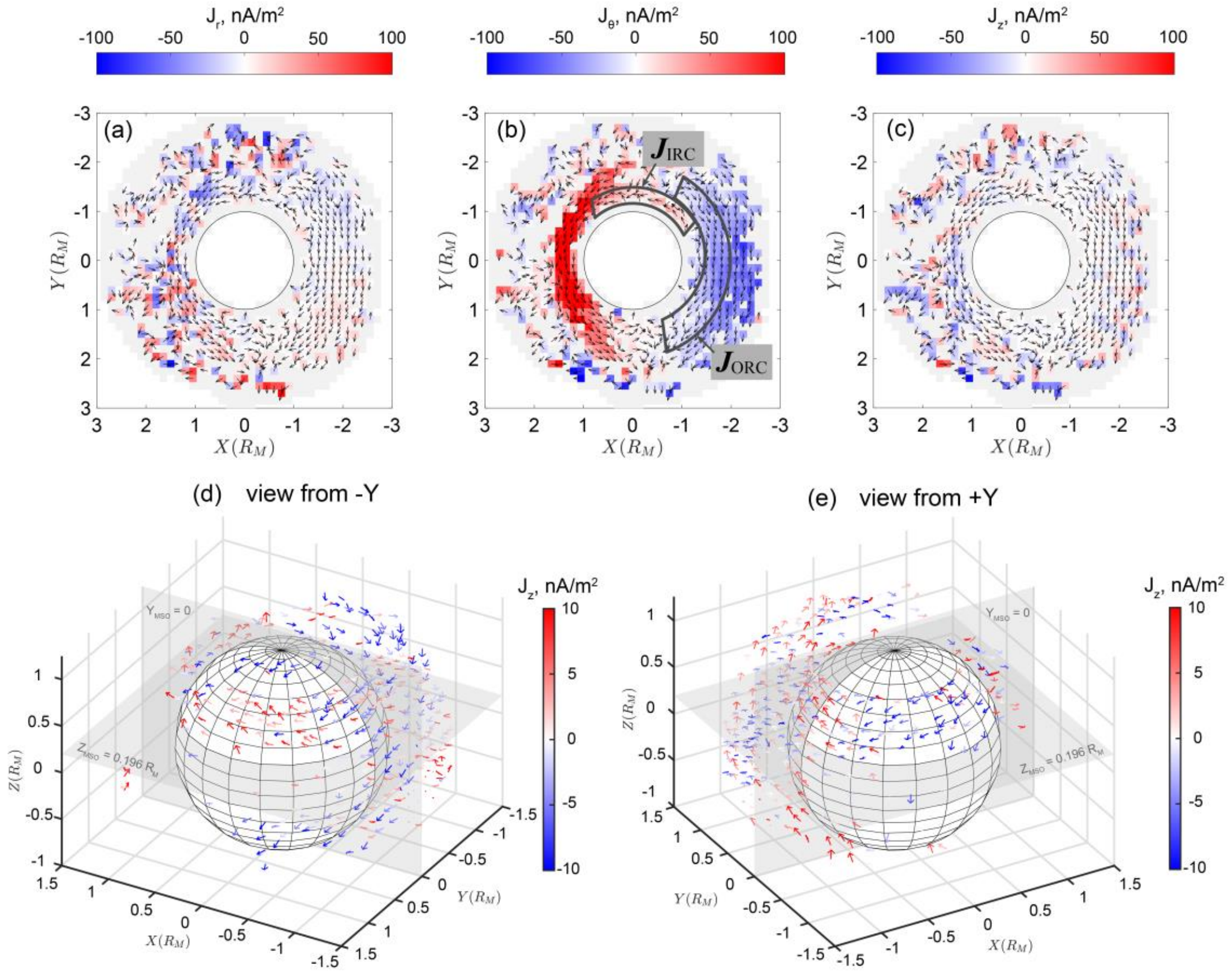

Figure B3. Slices in the X-Y plane and side view of the three-dimensional map of the Mercury's current systems. Panels a-c show a slice in the X-Y plane within $0.15\ R_M \le z \le 0.30\ R_M$. In panel d-e, the axis range is limited to $|x| < 1.5\ R_M$, $|y| < 1.5\ R_M$ and $|z| < 1.25\ R_M$. Each column of panels is colored by the components of the current density in cylindrical coordinates, respectively. Arrows in panels a-c show the current direction in the projected plane of each panel. The black circles in panel a-c and the sphere in panel d-e represents the Mercury. The grid cells in grey represent no valid measurements. The regions used to identify $\boldsymbol{J}_{\mathrm{ORC}}$ and $\boldsymbol{J}_{\mathrm{IRC}}$ are indicated by the grey enclosed areas in panel b. The magnetic equatorial plane for reference in panels d-e is indicated by the translucent surface ($Z = 0.196\ R_M$). Arrows represent the current density vector. To present the $\boldsymbol{J}_{\mathrm{ORC}}$ distinctly, arrows with $J_\theta > 0$ are not shown. Color of arrows in panels d-f represents the z-component of the current density in cylindrical coordinates.

In summary, the uncertainty in the derived current pattern would be large so that the polar ring current could not be identified. This current may not be explained by the pressure gradient near the cusp because the local time distribution of this current is different from that of the cusp. Perhaps, BepiColombo data, which would be available in the near future, would be needed for further analysis. Nevertheless, trial to derive the current perpendicular to the background magnetic field

would be new in the polar region. I feel the manuscript would be of interest to certain community if it is appropriately revised. Because of this, I have added general and specific comments as listed below.

As a general comment, the authors derived currents in Cartesian coordinates (e.g., Figures 2 and 3). If this would be presented in cylindrical coordinates, identification of ring current or partial ring current would be easier.

Thank you for your comment and suggestion. Following your advice, we converted most of the currents to the cylindrical coordinate system, as shown in Figure B3-B4 (Fig. 3-4 in the revised manuscript). The results in cylindrical coordinates indeed make ring current structures much easier to identify.

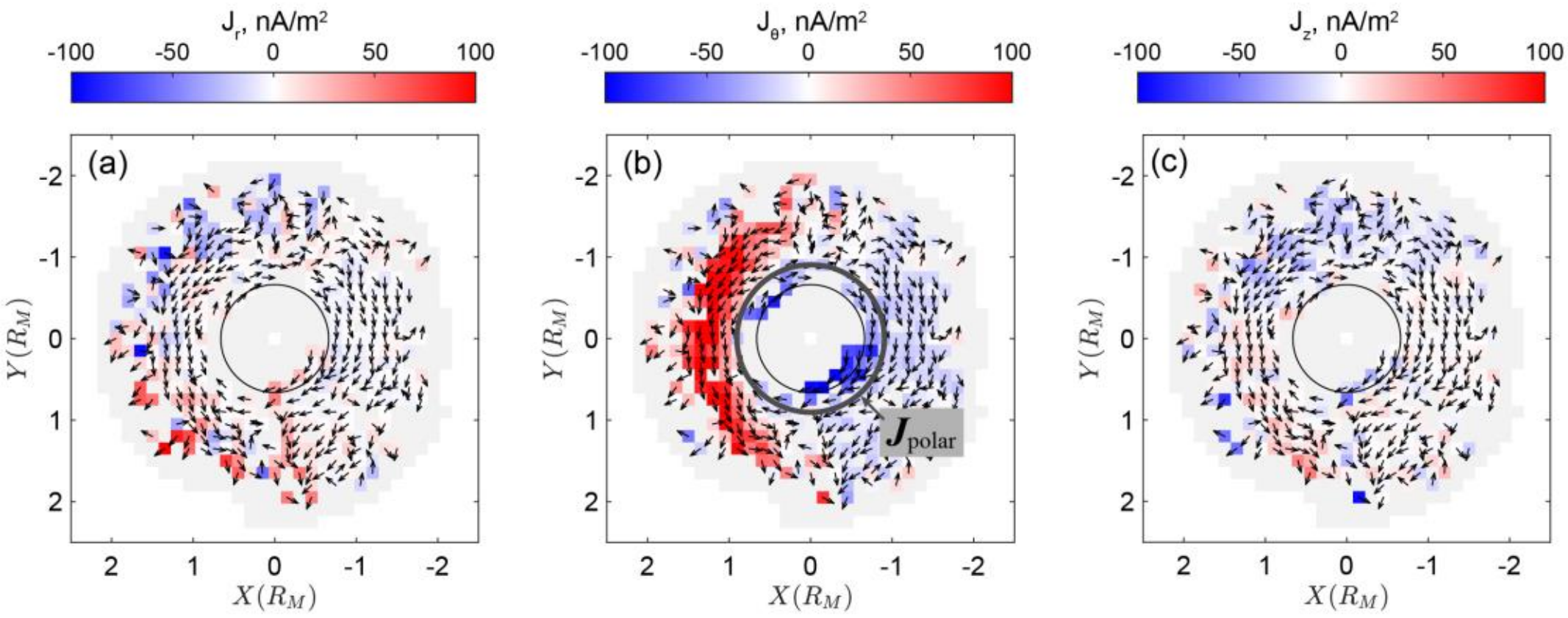

Figure B4. Slices of the Mercury's current systems in the X-Y plane within 0.75 $R_M \leq z \leq 1.20$ R_M. Each column of panels is colored by the components of the current density in cylindrical coordinates. Arrows show the current direction in the projected plane of each panel. The black circle in each panel represents the Mercury. The grid cells in grey represent no valid measurements. The region identified as $\boldsymbol{J}_{polar}$ is indicated by the grey circle in panel b.

The second comment is the methodology the authors used to derive the current. Although the authors mentioned it as the curlometer technique, this is usually applied to four spacecraft measurements (Dunlop et al., 1988). Instead, the authors applied the first-order central finite difference method to the Cartesian grid data as performed in Shi et al. (2022). Therefore, the technique the authors employed could be noted as the latter.

Thank you for your comment and suggestion. We agreed with your strict distinction between these two methods. We now explicitly distinguish the first-order central finite difference method from the classical four-spacecraft curlometer in the revised manuscript.

The third comment is regarding field-aligned currents (FACs). Previous reports such as Pump et al. (2024) identified FACs from MESSENGER magnetic field observations in the polar region. Is it possible that the currents identified in the polar region by the authors are FACs instead of ring currents?

Thank you for your comment. The $\boldsymbol{J}_{polar}$ is not a field-aligned current. Field-aligned currents flow parallel or antiparallel to the magnetic field direction, and polar field-aligned currents are dominated by the Z-component in both the MSO and cylindrical coordinate systems. Near the north pole, the

background magnetic field is predominantly directed along +z, whereas the reconstructed $\boldsymbol{J}_{polar}$ is dominated by its azimuthal component and is therefore largely perpendicular, rather than parallel, to the local magnetic field.

Specific comments

Line 54. when in -> during
Corrected.

Lines 82-84. Figure 2 could be mentioned here in addition to Extended Data Figures 6 and 7.
Corrected.

Lines 84-85. The definition of the median relative uncertainty could be mentioned here.
The median relative uncertainty for the current components is defined as the median value of uncertainty of current density divided by the averaged current density. We added the definition in the revised manuscript.

Line 87. close zero -> zero
Corrected.

Line 90. are shown in -> are explained in the Methods section and shown in
Corrected.

Line 120. Jx<0 within y<0, Jx>0 within y>0 -> Jx>0 within y<0, Jx<0 within y>0
Thanks for the reminding. Note that in the revised manuscript, we converted the current density from Cartesian coordinates to cylindrical coordinates and we revised the associated descriptions.

Lines 147-148. Slices in ... measurements -> Slices of the Mercury's current systems in the X-Y plane at different Z values
Corrected.

Lines 153-155. I cannot find bolded black circles in Figures 3a and 3b.
We deleted the bolded black circle in the figure and the associated description.

Line 159. to -> at
Corrected.

Line 172. 21 -> 20
Corrected.

Lines 173-175. It could be difficult to separate J_IRC and J_MP since both could overlap without a gap.
Thanks for the comment. We corrected the sentence to "at noon, it is difficult to distinguish between the $\boldsymbol{J}_{IRC}$ and the $\boldsymbol{J}_{MP}$ since they are both in direction of counter-clockwise and there is no clear

boundary between these two currents."

Line 184. polar data -> data

Corrected.

Line 189. azimuth angle -> local time

Corrected.

Line 190. J_Phi>0 -> J_Phi<0

Corrected.

Lines 201-202. the magnetic induction equation in a steady-state case -> the Ohm's law

Corrected.

Line 219. middle latitude -> low altitude

Corrected.

Line 250. anisotropic -> isotropic

Corrected.

Lines 253-254. The phrase, if protons .. regions, could be deleted.

It is deleted.

Lines 255-256. Why Figure 5 looks different from Figures 3a-c?

Thanks for the comment. We attribute this discrepancy to the limited number of effective plasma sampling channels and low temporal resolution of FIPS onboard MESSENGER, which leads to an underestimation of the plasma pressure.

We have added a dedicated discussion in the revised manuscript to explain this difference between the two results.

Lines 257-259. This may not be the case because ions would perform gradient B/curvature drift in the westward direction due to the Mercury's background magnetic field and this direction is opposite for electrons. However, the current J_IRC would be eastward after the magnetization current is added.

Thank you for pointing this out. As you and Reviewer # 3 suggested, ring currents generally result from magnetization of charged particles. Part of the magnetization current cancels the component of the current due to guiding-center drift of charged particles. The remaining magnetization current, which is proportional to the plasma pressure gradient, presents the circular current. We corrected and updated the entire discussion and description of the ring current formation mechanism in the revised manuscript.

Lines 291-292. Magnetic field vectors are not shown in Figures 1b and 1c.

Thank you for pointing this out. We revised the sentence to "The magnetic field near the north pole

is predominantly z-directed, meaning that the magnetic field vector near the north pole points almost perpendicularly to the planetary surface."

Lines 298-300. Which values of density and temperature were adopted?

The values of the density and temperature of H^+ ions adopted from Sun et al. (2022; doi:10.1029/2022JA030280) are 20 cm^{-3} and 0.7 keV. The values of the density and temperature of H^+ ions adopted from Raines et al. (2014; doi:10.1002/2014JA020120) are 21.4 cm^{-3} and 0.5 keV.

Line 307. How characteristics of J_polar appear in simulation results?

Aizawa et al. (2021, doi:10.1016/j.pss.2021.105176) compared the simulation results of two 3D magnetohydrodynamic models (AMRVAC, Yagi) and two 3D hybrid simulation models (AIKEF, Amitis) regarding the interaction between the dayside magnetosphere of Mercury and the solar wind. There exist current characteristics consistent with $\boldsymbol{J}_{\text{polar}}$ in observations in the results of the AMRVAC and Yagi models. The J_y component of the current density of AMRVAC model is less than 0 (i.e., negative) in the dayside polar regions and greater than 0 (i.e., positive) in the nightside polar regions (seen in the black rectangles of Figure B5). This is consistent with the characteristics of $\boldsymbol{J}_{\text{polar}}$ in observations from our manuscript (Figure B5b-B5c).

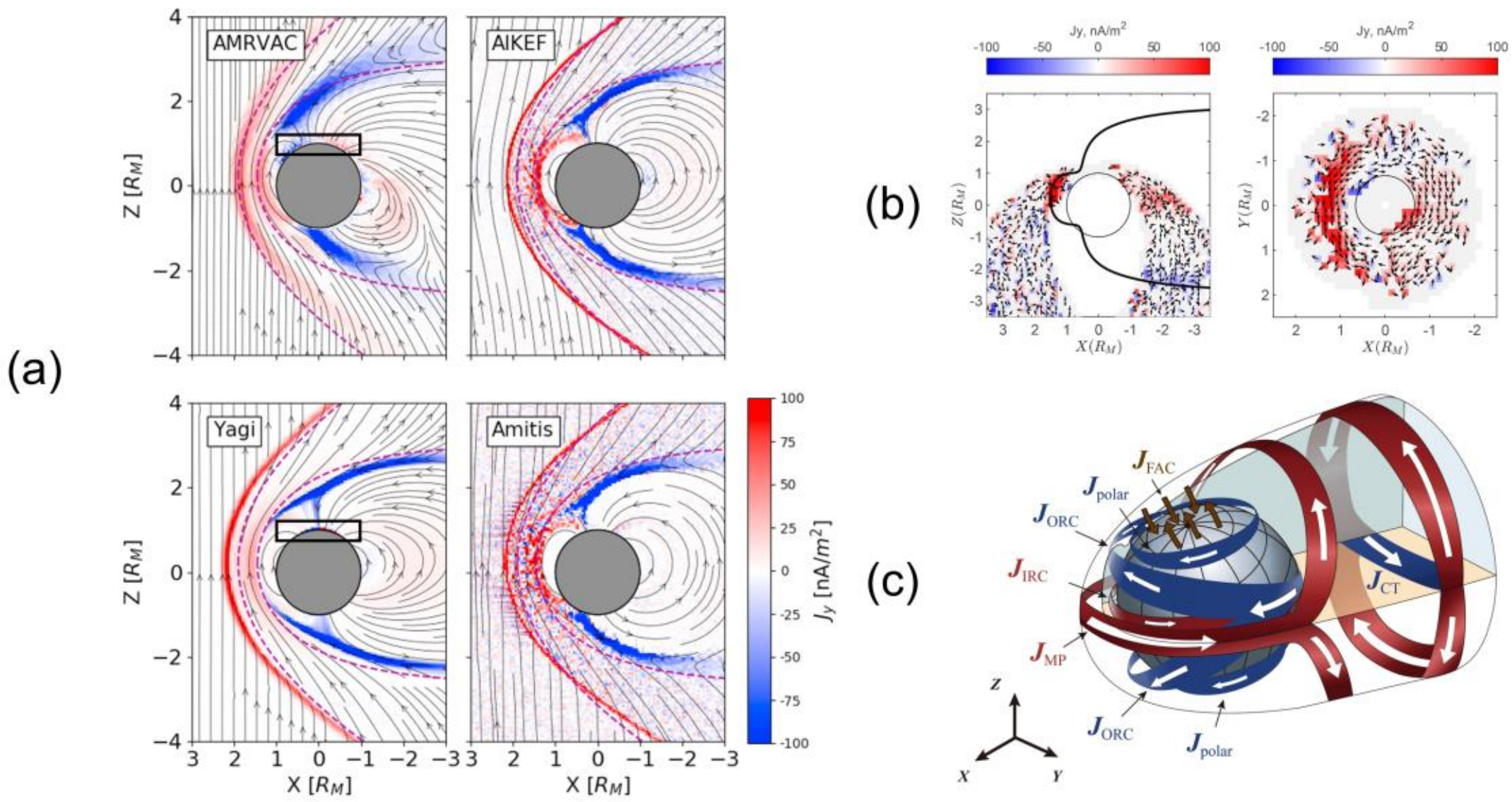

Figure B5. (a) Current density of simulation results from AMRVAC, AIKEF, Yagi and Amitis (Figure 4 from Aizawa et al., 2021). The y component of the current density in X-Z plane. Black lines with arrow represent the magnetic field line and dashed purple lines are the empirical models of bow shock and magnetopause. (b) Current density of observational results (Figure 2e and Figure 3e from our manuscript). (c) Cartoon of current system in Mercury's magnetosphere (Figure 6 from our manuscript). The black rectangles in panel (a) represent the regions of 0.75 $R_M \leq Z \leq 1.20\ R_M$.

In the revised manuscript, Descriptions of comparation between the AMRVAC model and our observations was added.

Line 357. electron -> kinetic

Corrected.

Line 368. consider in investigating the dynamic -> the dynamics
Corrected.

Line 510. pectrometer -> spectrometer
Corrected.

We sincerely appreciate your critical comments and helpful suggestions. They have greatly improved the rigor, clarity, and physical interpretation of our study.

Reply to Prof. Stepanova / Reviewer #3:

In their manuscript, "Global Current Systems in Mercury's Magnetosphere," Rentong Lin et al. analyze five years of magnetic field and plasma measurements obtained by the MESSENGER satellite. The authors propose a reconstruction of the global current system, comprising a magnetopause current, a cross-tail current, inner and outer equatorial ring currents, and a newly identified polar ring current. The authors argue that both the equatorial and polar ring currents are consistent with predictions of the plasma drifting model in regions where a pressure gradient takes place. These currents could reshape Mercury's magnetosphere, revealing new dynamics and energy transfer processes.

The experimental results presented here provide novel insights about the global current systems in Mercury's magnetosphere and warrant publication in Nature Communications. Nevertheless, the manuscript requires substantial revision.

Major points:

1) The introduction should be rewritten to ensure it is accessible to readers unfamiliar with Mercury's magnetosphere. It should provide a clear overview of what is currently known about Mercury's current system while highlighting the key questions that remain unanswered.
Thanks for this helpful suggestion. We substantially revised the Introduction to make it more accessible to readers who may not be familiar with Mercury's magnetosphere. In the revised version, we first summarize the distinctive properties of Mercury's magnetosphere and why current systems are important for understanding its large-scale dynamics, then review the main current systems previously reported from observations and simulations, and finally highlight the key open questions that motivate the present study.

2) Consider integrating parts of the "Obtaining current using magnetometer data" section into the next section and moving the technical details to the Methods section.
Thanks for the suggestion. We streamlined the Results section by retaining only the information needed to understand the reconstruction procedure, and moved the technical details of the current-

density calculation and uncertainty analysis to the Methods section.

3) Results for the global current system in Mercury’ s magnetosphere were obtained using Mercury Solar Orbital (MSO) coordinates as shown in Figures 2 and 3. However, this causes Jx color code to jump whenever the Y-coordinate flips sign. A similar issue affects the Jy component, where sign changes in the X-coordinate obscure the actual direction of the current. This makes it challenging to determine whether the analyzed currents are flowing clockwise or counterclockwise. At the same time, Figure 4 displays the azimuthal component of the current vector, providing a much clearer and more intuitive visualization. I suggest the following improvements to the final stage of the analysis:

- Separate the analysis of the currents surrounding Mercury from the tail current.
- For the currents surrounding the Mercury, transform the Cartesian components (Jx, Jy, Jz) into spherical coordinate system. Cylindrical system might also work. You can still keep the MSO coordinates for the positions of current vectors, but replace the (Jx, Jy, Jz) color coding with values corresponding to spherical or cylindrical components. This will allow for a clear distinction between clockwise and counterclockwise currents. This also makes the outer equatorial ring current easy to identify, as it is marked by a sign change in either the polar angle (latitude or colatitude) or the Z-component, depending on the chosen coordinate system.
- Combine the results shown in Figures 2a-c with those in Figure 3. Figures 2d-f can also be incorporated into this figure.
- For the tail current, the Cartesian components (Jx, Jy, Jz) can still be used (Figures 2 g-i), but they should be relocated to a new figure.

Thanks for this excellent suggestion. Following your advice, we now present the current systems surrounding Mercury primarily in cylindrical coordinates, which makes the clockwise and counterclockwise current patterns much easier to identify. We retain MSO Cartesian coordinates for the cross-tail current in the magnetotail, where that representation remains the most natural.

To improve clarity, we also reorganized the figures by current type: the magnetopause current, cross-tail current, equatorial ring-current-like system, and polar high-latitude current are now shown in separate figures (Figure C1-C4). This structure makes the different current systems easier to distinguish and discuss.

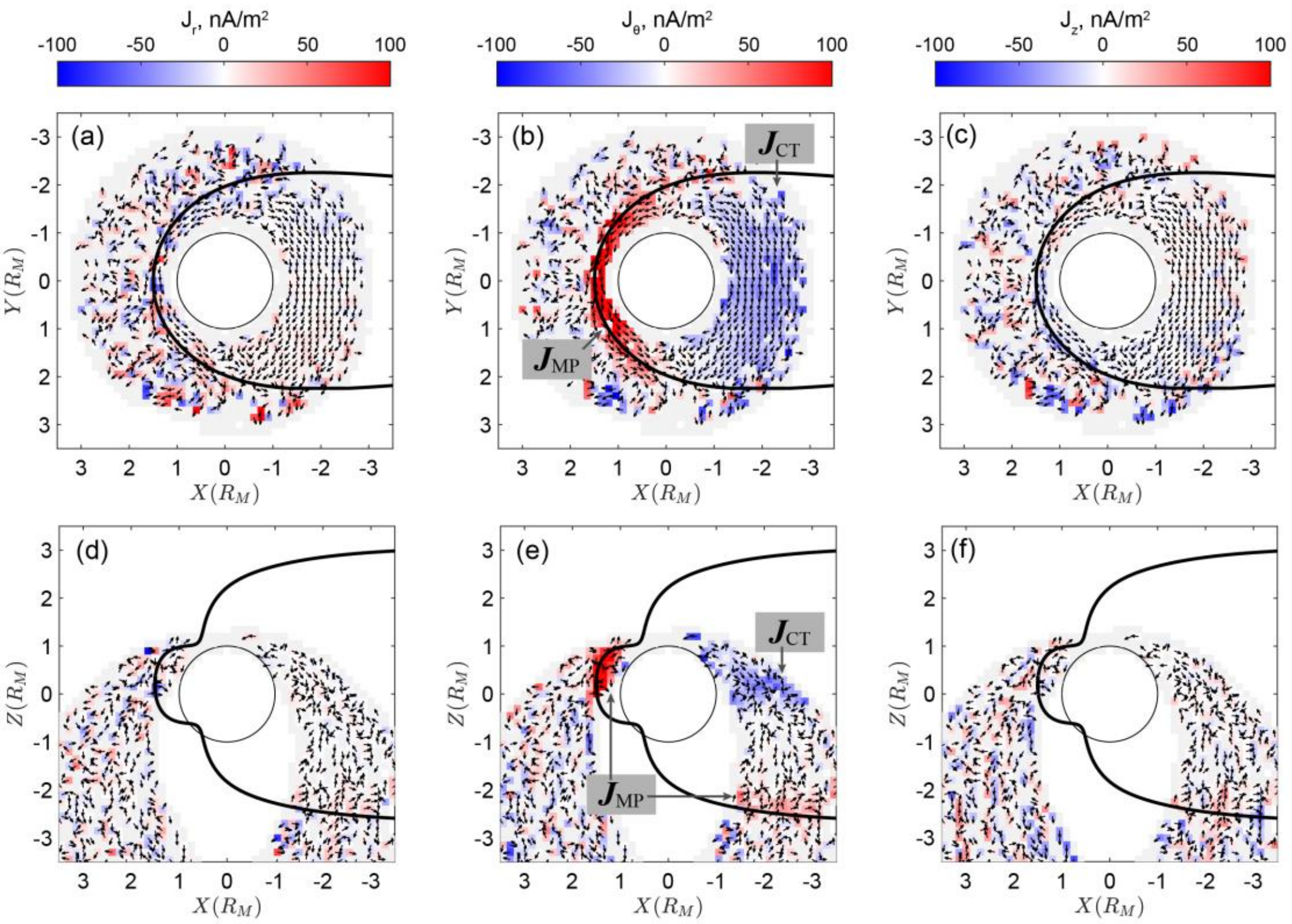

Figure C1. Magnetopause current

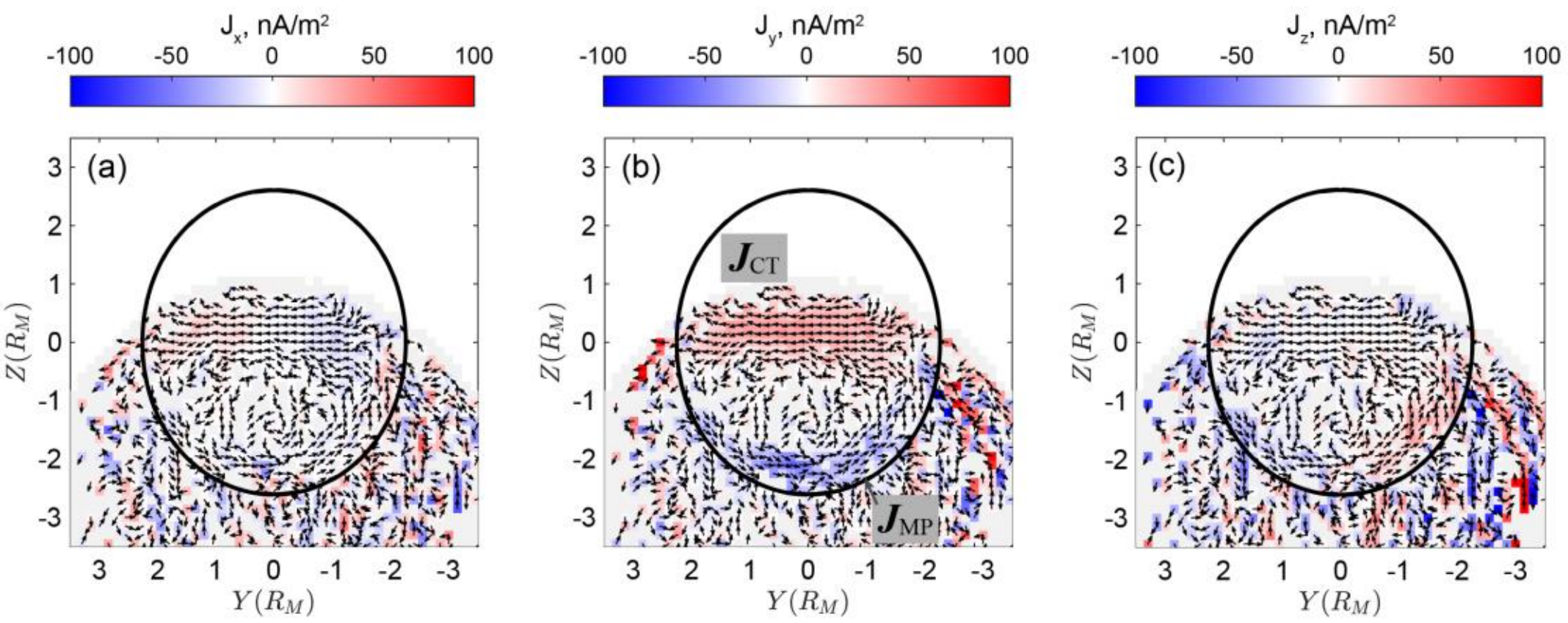

Figure C2. Cross-tail current

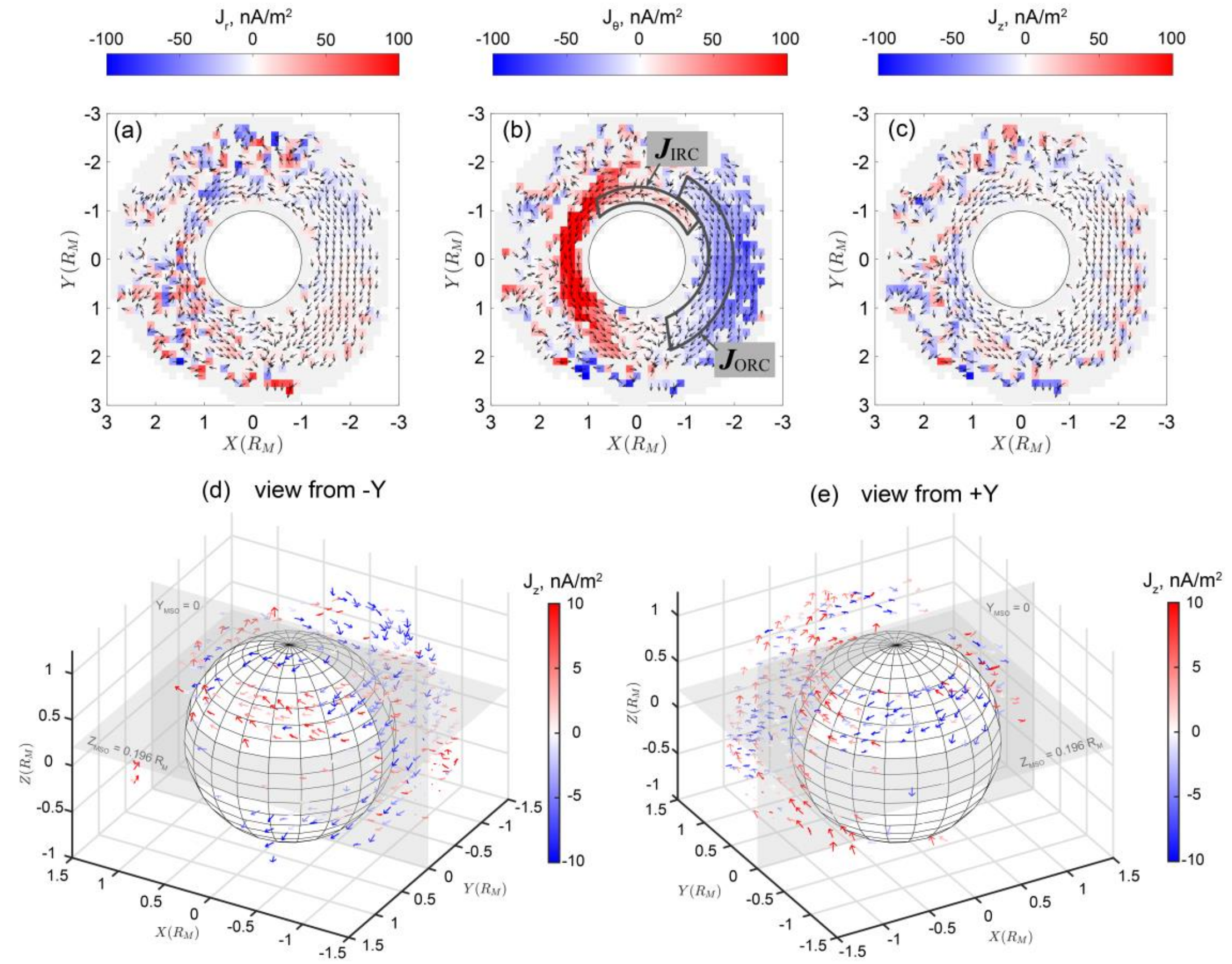

Figure C3. Equatorial current

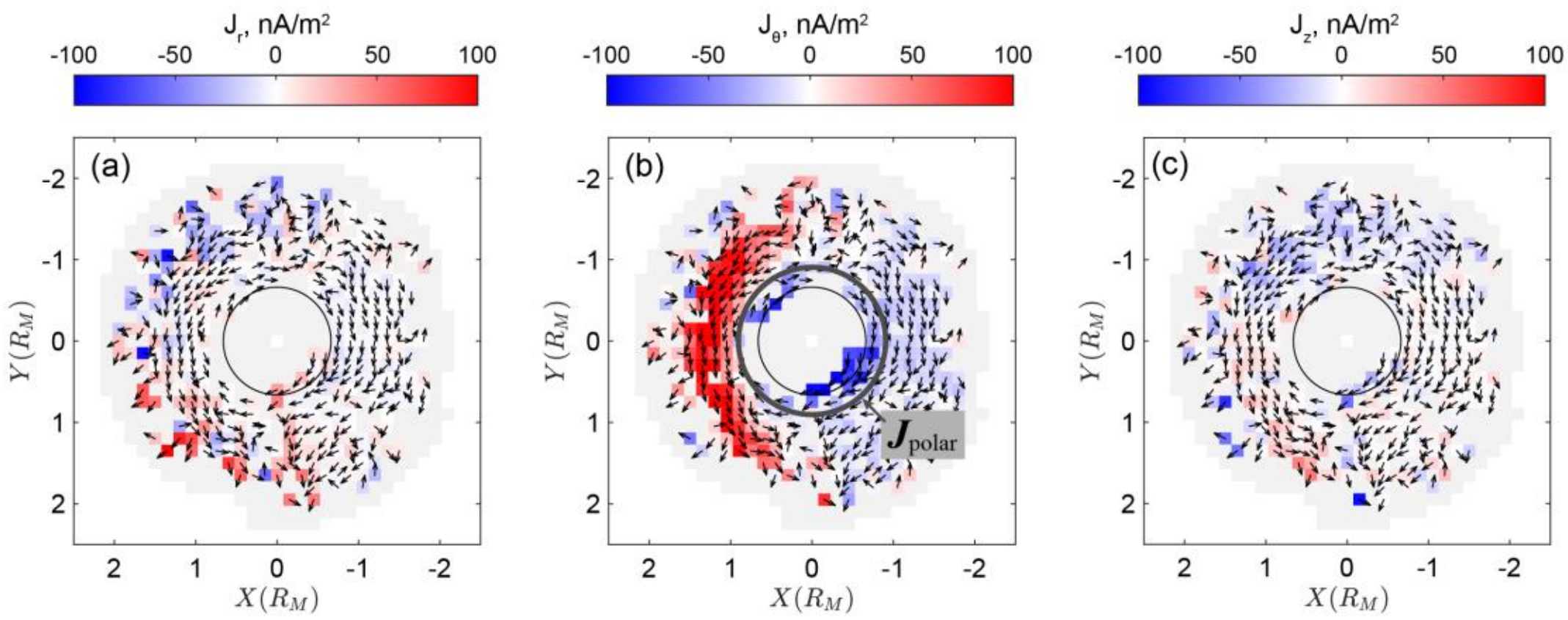

Figure C4. Polar ring current

4) While Figure 1, showing the spatial distribution of the magnetic field's absolute value (|B|), provides useful information, it is entirely disconnected from the subsequent analysis. It would be more effective to remove Figure 1 and add the corresponding |B| slices as an additional column to the new figures described above. Comparing the magnetic field and currents might yield new insights.

We agree that the primary focus of the paper is the current systems rather than the magnetic-field magnitude itself. We therefore moved the original magnetic-field-magnitude figure to the Extended Data material, while keeping it available as supporting information for the reconstruction procedure.

Additionally, we attempted to compare the magnetic field and current results in the same regions, but no additional meaningful conclusions could be drawn. For this reason, we have not added the corresponding magnetic field slices to the figures.

5) The outer equatorial ring current proposed by the authors (lines 157-167) finds a direct analogue in the Earth's magnetosphere: the cut ring current, initially described by Antonova and Ganishkina (2000) and Antonova (2003, 2004). Comprehensive details regarding this current system can be found in Subsection 3.4 and Figure 5 of Ganushkina et al. (2017, Reviews of Geophysics, doi: 10.1002/2017RG000590), a paper cited by the authors as [35]. The explanation for this current proposes that it flows along a surface where the magnetic field strength reaches its minimum along a given field line. While the Earth's magnetic field is predominantly dipolar near the planet, solar wind compression on the dayside displaces these minimum-B locations away from the magnetic equator. This displacement causes the current to split into two distinct branches. This interpretation, grounded in fundamental concepts of plasma physics, offers a more robust and physically consistent explanation than the Shabansky orbit concept cited by the authors (lines 273-281). Please discuss the similarities between the outer equatorial ring current and the cut ring current and incorporate the corresponding references.
Thanks for this valuable suggestion. In the revised manuscript, we now discuss the dayside $\boldsymbol{J}_{\mathrm{ORC}}$ in explicit connection with the cut ring current known from Earth's magnetosphere. In particular, we note that solar-wind compression can displace the minimum-B locations away from the magnetic equator, leading to off-equatorial branches of the ring-current-like structure. We have revised the text accordingly and added the recommended references.

6) The authors state that "Ring currents generally result from opposite drift of ions and electrons in a region with pressure gradient" (lines 249-262). This attribution of the ring current to particle drift is incorrect, despite its prevalence in many space physics textbooks. In reality, the component of the current due to guiding-center drift is entirely canceled by a portion of the magnetization current. The remaining magnetization current, which is proportional to the plasma pressure gradient, is what generates the ring current. As clearly demonstrated in another review by Ganushkina et al. ("Defining and resolving current systems in geospace," Ann. Geophys., doi:10.5194/angeo-33-1369-2015, Subsection 2.4, "Drifts and currents"), stationary transverse currents in geospace are generated by plasma pressure gradients. Consequently, changes in the ring current direction can be readily explained by the presence of a plasma pressure maximum, which leads to a reversal in the sign of the slope of the plasma pressure radial profile. This also will reinforce the explanation of the existence of the polar ring current.
Thanks for this important correction. In the revised manuscript, we no longer attribute the ring current simply to the opposite drift of ions and electrons. Instead, we now explain that the current relevant here is the residual magnetization current associated with the plasma pressure gradient, after cancellation of the guiding-center-drift contribution. We also clarify that the reversal of current direction across the pressure maximum naturally explains the coexistence of $\boldsymbol{J}_{\mathrm{ORC}}$ and $\boldsymbol{J}_{\mathrm{IRC}}$, and strengthens the interpretation of $\boldsymbol{J}_{\mathrm{polar}}$.

7) The authors examined the influence of the solar wind on polar currents (Extended Data Fig. 3)

by dividing the dataset according to heliospheric distance, finding no substantial differences. In my view, it is crucial to extend this analysis to other current systems. For instance, given that solar wind dynamic pressure compresses the dayside magnetosphere, one might expect an observable impact on the outer equatorial ring current.

Thank you for this excellent suggestion. Our original motivation for dividing the dataset by heliocentric distance and calculating polar currents separately was to test whether the polar ring current is a spurious signal arising from magnetospheric disturbances driven by varying solar wind conditions. As you suggested, we have now calculated the current densities at different heliocentric distances to further assess the reliability of our current density estimates.

We find that when Mercury is closer to the Sun, the current densities of both the Mercury magnetopause current and the cross-tail current are higher, and the magnetopause current lies closer to Mercury's surface. Additionally, the inner equatorial ring current is clearly observable when Mercury is farther from the Sun, but its signature becomes barely discernible when Mercury is near perihelion. These trends are consistent with the expected compression of Mercury’s magnetosphere under stronger solar-wind forcing, and therefore support the robustness of the large-scale current patterns inferred here.

It should be noted that the signatures of polar currents are detectable at all heliocentric distances, but the currently available dataset size is clearly insufficient to resolve the detailed differences in the polar ring current under different solar wind conditions.

All these new results are added in the Extended Data Figures, and a brief summary of the findings has been added to the main text. We present these results as sensitivity tests on the statistical reconstruction rather than as separate climatological studies of solar-wind control.

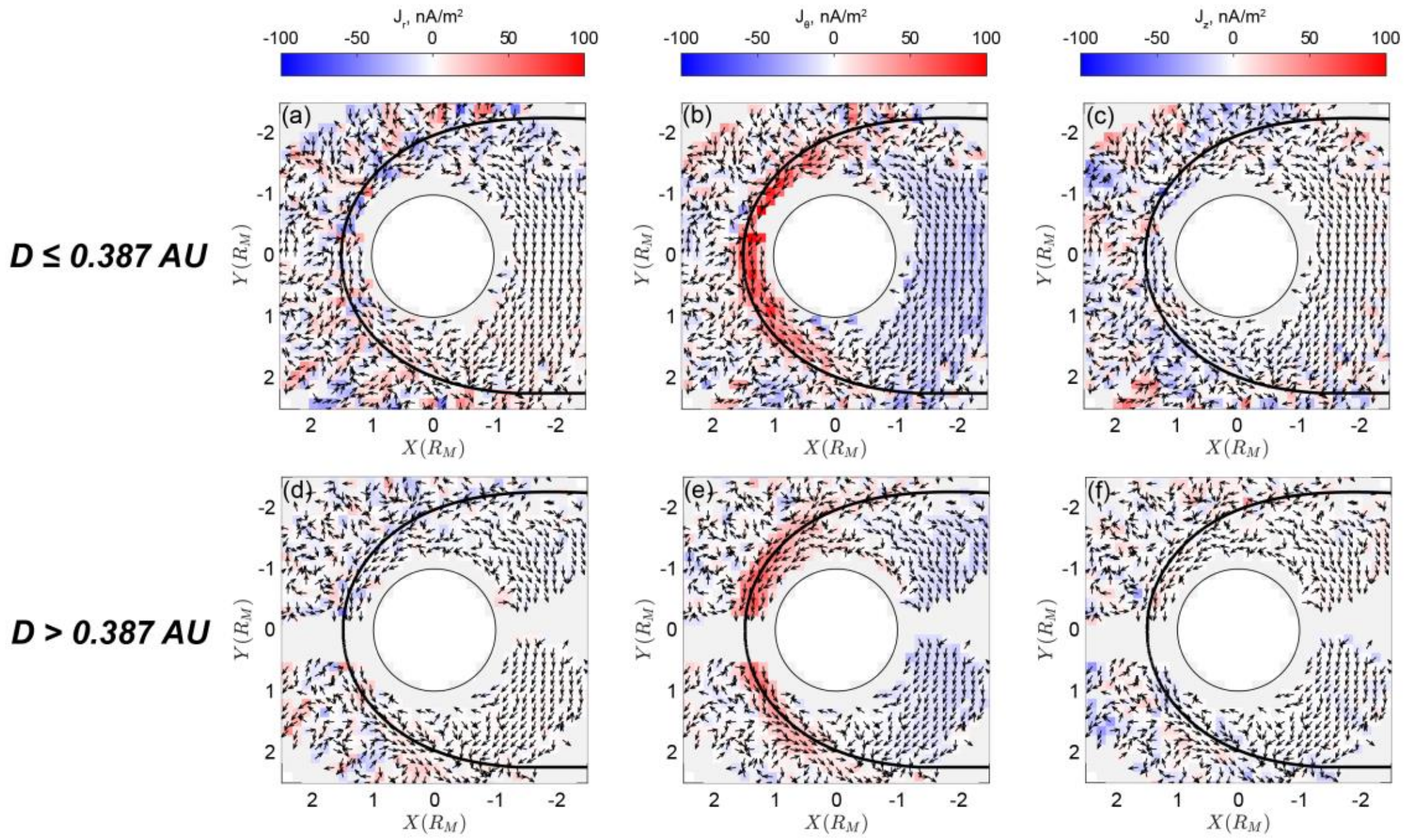

Figure C5. Magnetopause current at different heliocentric distances.

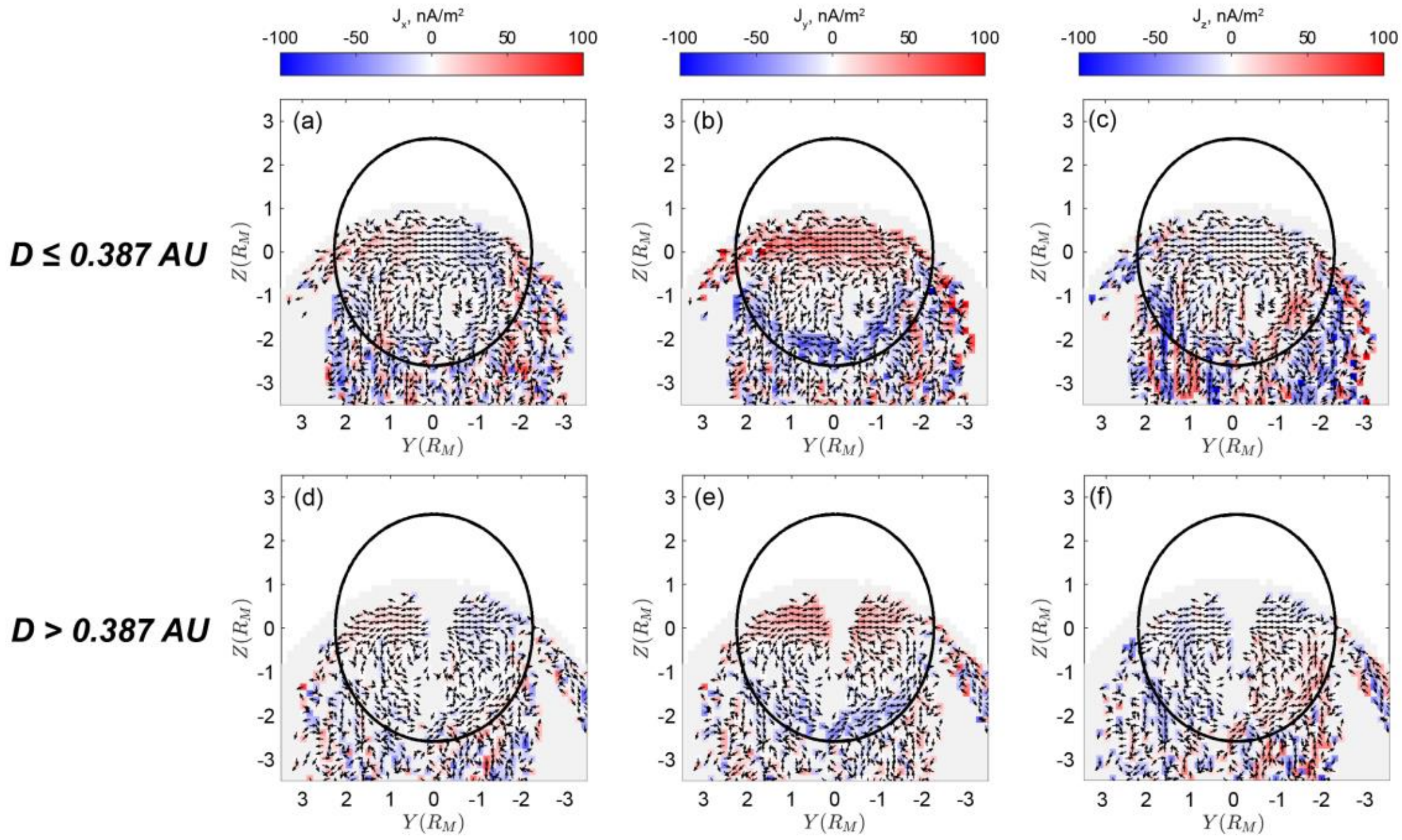

Figure C6. Cross-tail current at different heliocentric distances.

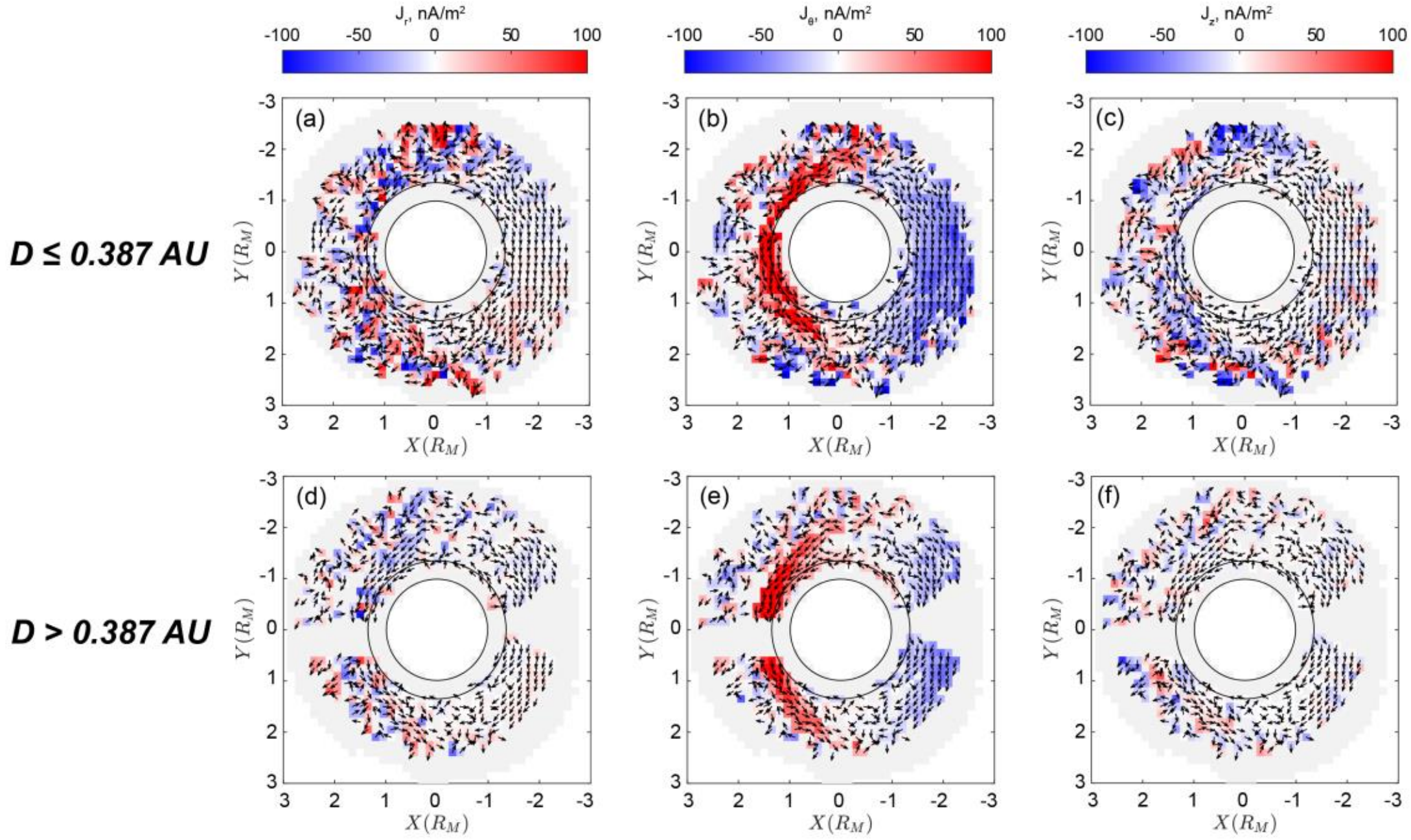

Figure C7. Equatorial ring current at different heliocentric distances.

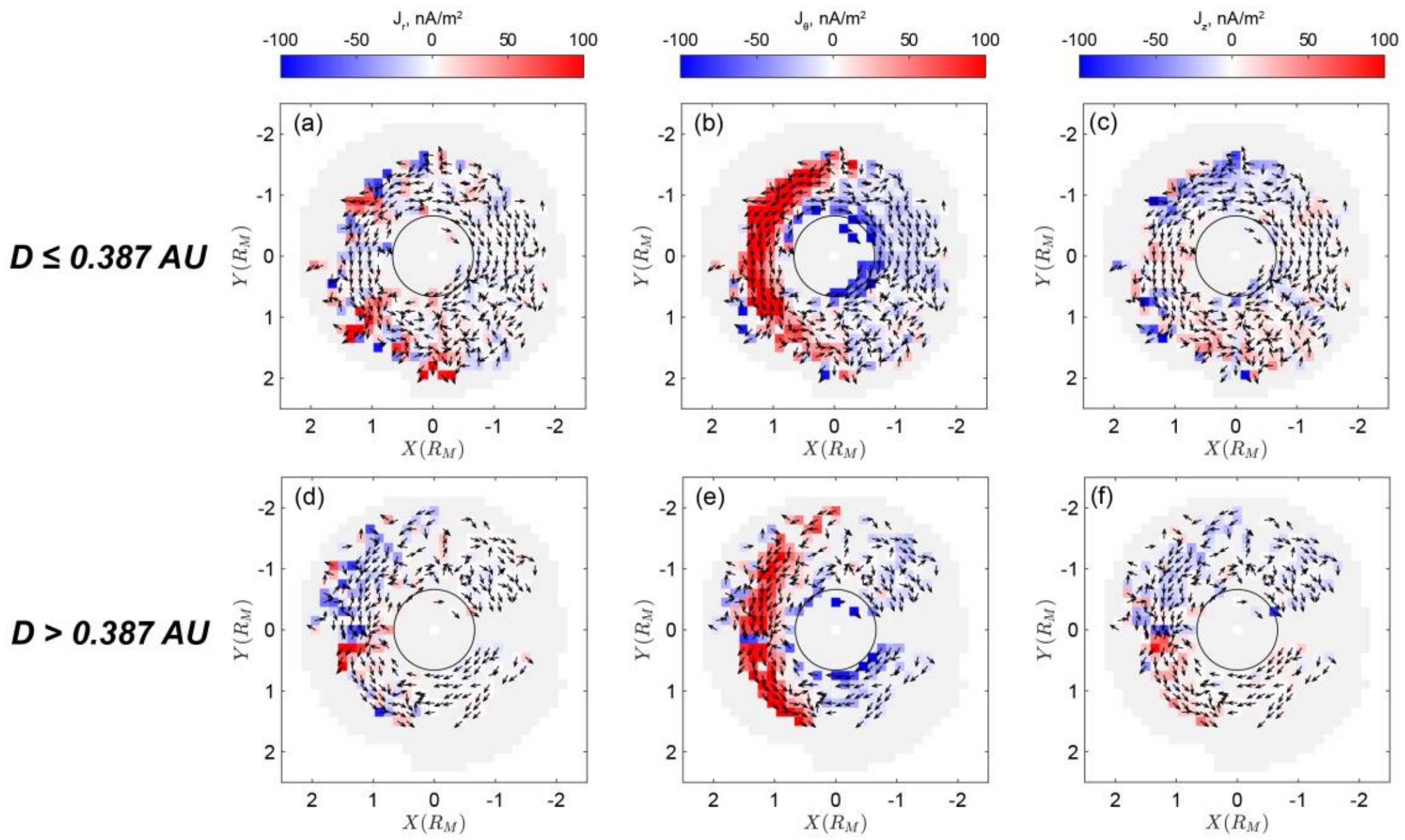

Figure C8. Polar ring current at different heliocentric distances.

Minor points:

lines 32-33

“.... its magnetic moment ranges from 190 to 223 nT・RM^3... “ Please, refrase to state clear that the model gives the values within the given range. Otherwise it gives a sensation that the magnetic moment could vary within the indicated range. Details about the Mercury’s magnetic moment can be moved to data analysis part.

Thanks for the comment. We clarified that this numerical range represents the interval of values derived from models, and moved this description to the Methods section.

lines 64-66

This paragraph is more suitable for the introduction

Thanks for the comment. In the revised manuscript, we moved this paragraph to the rewritten introduction.

line 67

A few words about the Messenger mission are needed before the description of the magnetometer, including the orbit.

Thanks for the comment. We added a brief overview of the MESSENGER mission, including its orbital characteristics, as suggested.

line 187

Please, check capture in Figure 4. Looking from the Northern pole, the predominant direction of the current is clockwise, that correspond to -JΦ, i.e. the azimuthal component is directed contrary to the azimuthal basic vector. This agrees with the color scale on the right side of the graph, but

contracts to the definition “$J\Phi > 0$ represents clockwise and $J\Phi < 0$ represents anti-clockwise”.
Thanks for the comment. The currents in this figure are converted to the cylindrical coordinate system and we corrected the corresponding definition in the main text.

line 590
Extended Data Fig. 5(a) The vertical axis should be Y. This figure is important, and I recommend to move it to the core part of the paper.
Thanks for the comment and reminding. We confirmed that the vertical axis of Extended Data Figure 5(a) should indeed be the Y-axis. We corrected this error in the revised manuscript and moved the figure to the main text.

With best wishes
Marina Stepanova

We sincerely appreciate your insightful comments and valuable suggestions. They have greatly improved the rigor, clarity, and physical interpretation of our study. We hope that our modifications address all your concerns. Thanks for your time and effort!

Reply to the Reviewers

We greatly thank the reviewers for the valuable comments/suggestions that we considered in the resubmitted manuscript. All revised parts are marked in red in the text. Detailed answers to the comments are given below:

Reply to Reviewer #1:

The authors greatly improved the study and made it clearer and physically justified. They have took into account all comments and suggestions, changed the text and figures and added additional figures for clarification of results. There is a minor comment: at lines 87, 160 – "…provided in the Method section …". But the section is called Methods. After this minor correction the paper can be accepted to the journal.

Thanks for the reminding. These typos are corrected. Thanks again for your previous valuable comments and suggestions!

Reply to Reviewer #2:

Each type of ring current is easier to be identified after the data are plotted in the cylindrical coordinates. The explanation in the manuscript has been clearer after the revision. Nevertheless, I have one comment. The current is related to the pressure gradient through the relation: JxB=grad P. The dayside portion of the polar ring current is related to the pressure gradient in the cusp as can be seen in the Extended Data Figure 11. However, there is not significant pressure variation near the location of the nightside polar ring current. The authors could explain that reason.

Thanks very much for this suggestion. The distribution of pressure observed by MESSENGER and from simulations are shown in Figure B1 and Figure B2, respectively. One can see that, on the nightside, there exists a pressure gradient pointing from high latitude to low latitude. The local magnetic field vector roughly points towards the Mercury surface. Therefore, the estimated local current direction by $\boldsymbol{J}_{\perp} = -\frac{\nabla P \times \boldsymbol{B}}{B^2}$ points to +Y-direction in MSO coordinate or clockwise viewed from the north pole, which is illustrated in Figure B3. However, it should be noted that the intensity of current density calculated by $\boldsymbol{J}_{\perp} = -\frac{\nabla P \times \boldsymbol{B}}{B^2}$ based on the plasms pressure observations is less than that calculated by $\boldsymbol{J} = \frac{1}{\mu_0}\nabla \times \boldsymbol{B}$ based on magnetic field observations. We think that it is because of the large deviation of plasma pressure from observations by FIPS/MESSENGER. The upcoming BepiColombo mission will provide more accurate measurements of plasma fluxes and the estimation of the $\boldsymbol{J}_{\text{polar}}$ should be more precise in the future work.

In the revised manuscript, we added explanations of direction of $\boldsymbol{J}_{\text{polar}}$ on the nightside.

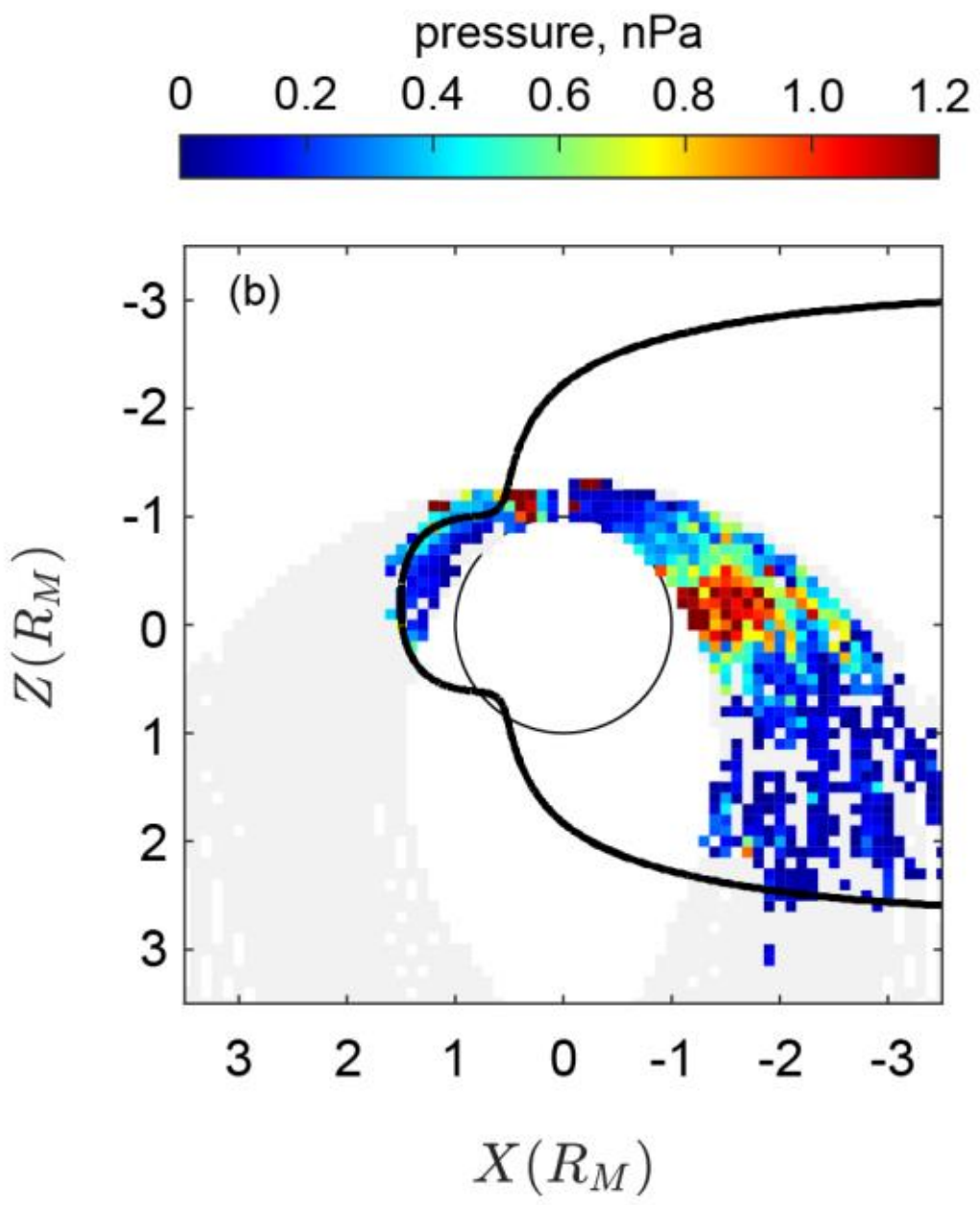

Figure B1 / Extended Data Fig. 12. Distribution of plasma pressure of H^+ ions in X-Z plane centered at Y=0. Distribution of plasma pressure derived from the Fast Image Plasma Spectrometer (FIPS) measurements (30). The grid cells in grey represent no valid measurements.

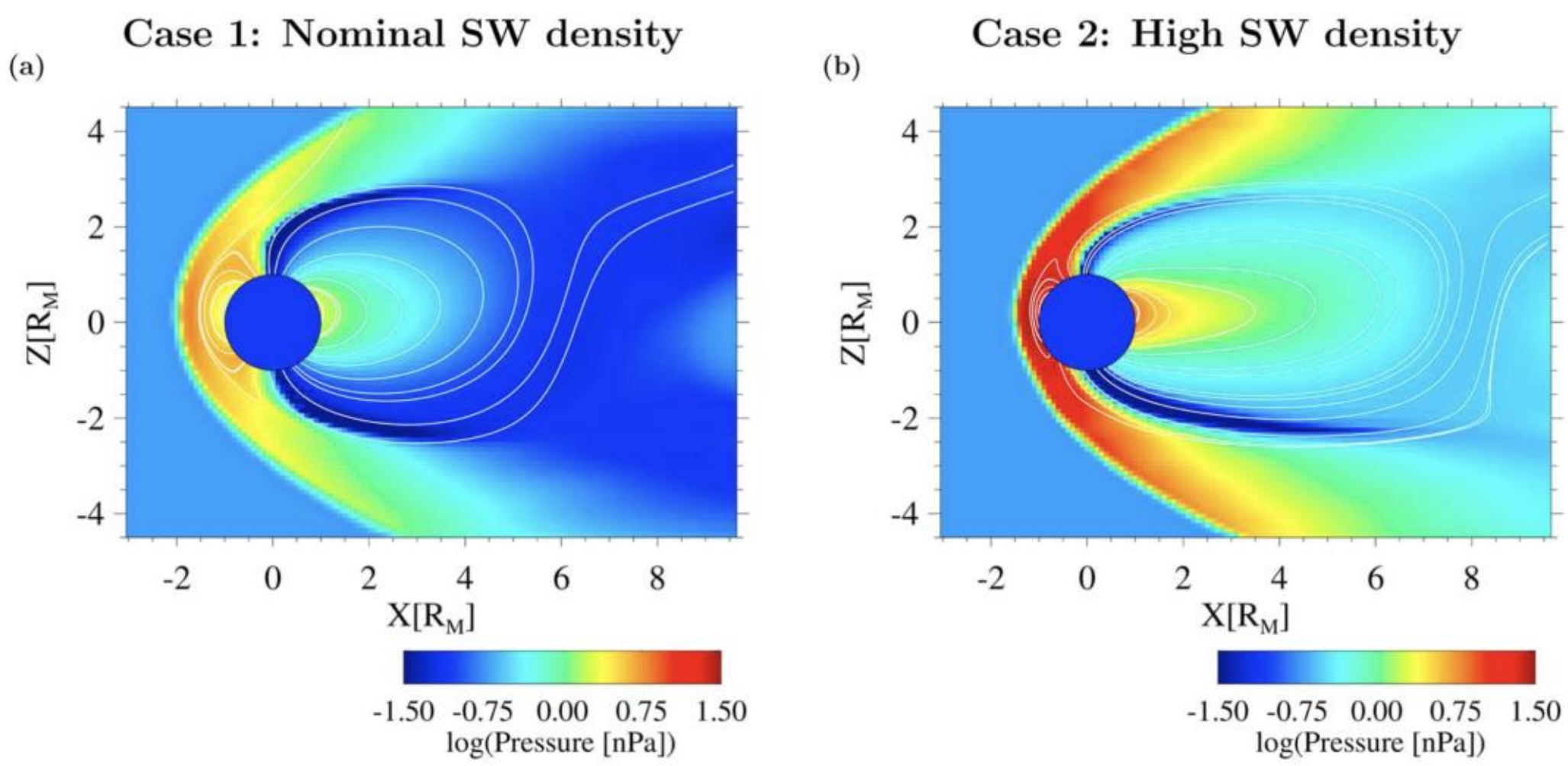

Figure B2. Distribution of plasma pressure of H^+ ions in X-Z plane centered at Y=0 adapted from Yagi et al. (2017).

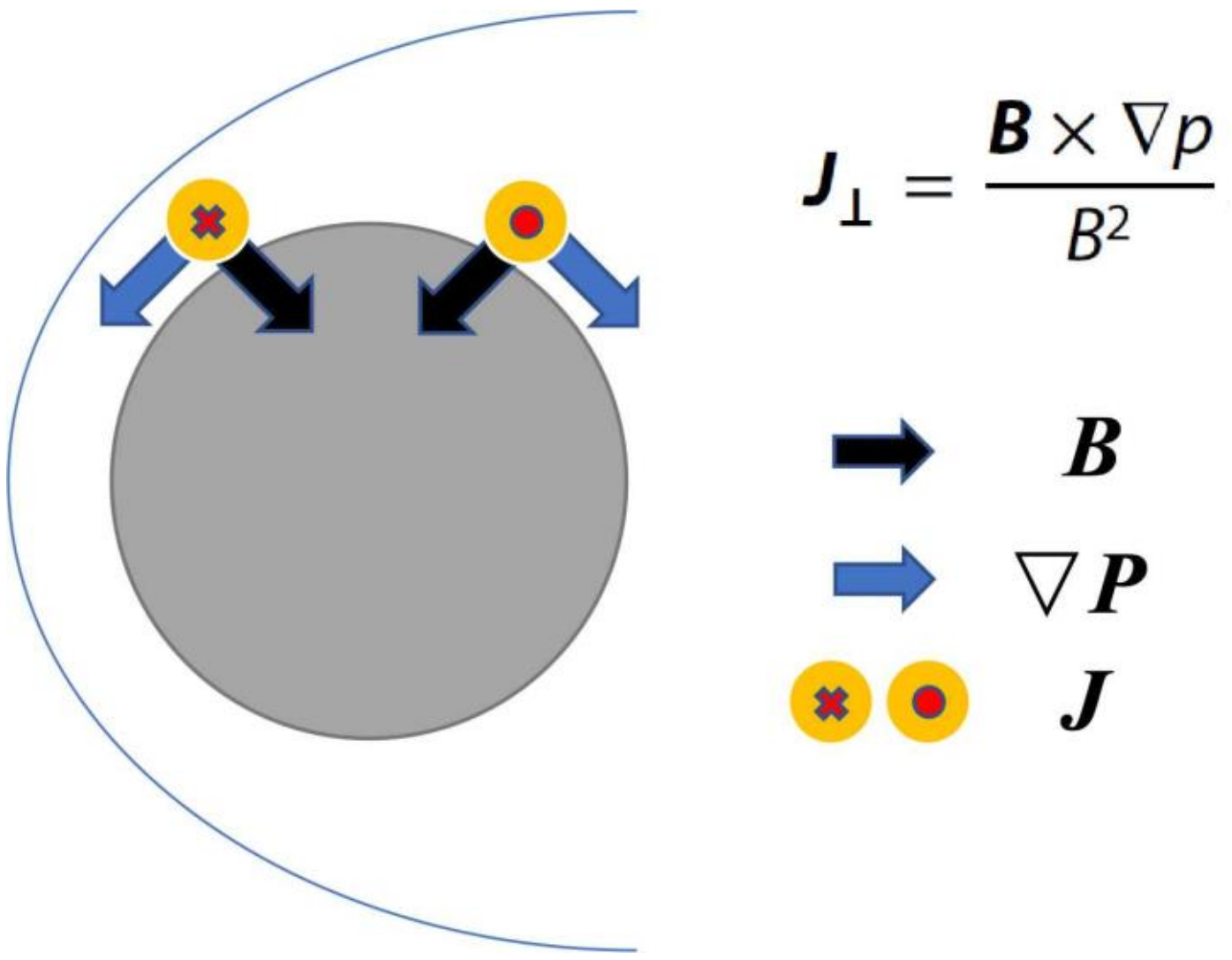

Figure B3. Illustration of direction of current related to pressure gradient in X-Z plane viewed from the dusk side (+Y).

Specific comments

Figure 3. (d) view from -Y -> (d) view from +Y. A similar correction may be made for panel e.
Thanks for the reminding. The descriptions were corrected in Figure 3.

Line 157. direction of counter-clockwise -> the counter-clockwise direction
Corrected.

Line 161. Is rho defined previously?
Thanks for the reminding. Definition of ρ was added in the revised manuscript.

Line 175. The confinement of the polar ring current to high latitudes or the separation between the polar ring current and the outer ring current would be clearer if the current distribution in the X-Y plane at Z=0.30-0.75 RM or those with finer Z resolution are shown.
Thanks for the suggestion. The current distribution in the X-Y plane in different ranges of z intervals are shown (Figure B4). One can see that the current density within 0.45 $R_M \leq z \leq 0.60$ R_M (Figure B4b) is smaller than those at equator and in polar regions (Figure B4a & B4c). It supports that the $\boldsymbol{J}_{ORC}$ and the $\boldsymbol{J}_{polar}$ are separated.

We added descriptions in the text and this figure in the extended data figure in the revised manuscript.

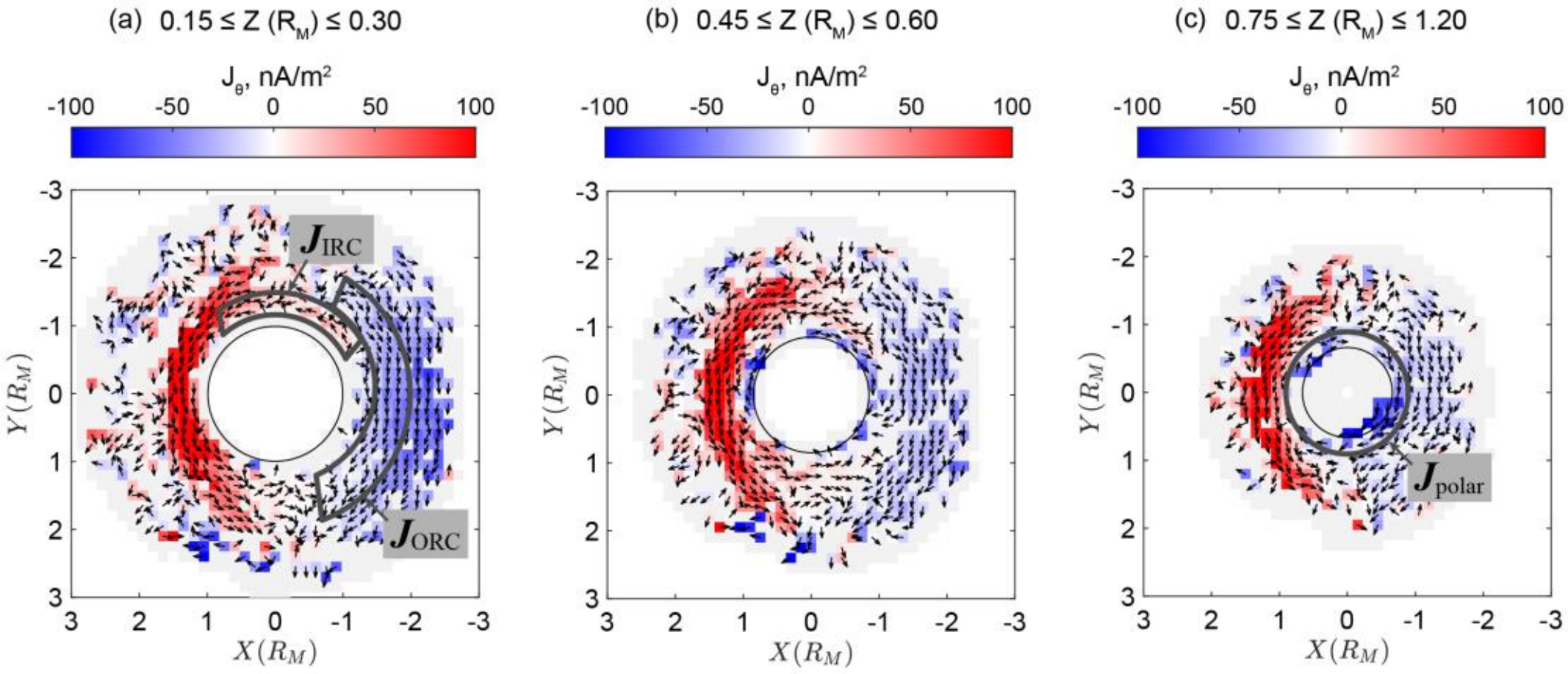

Figure B4. Current density in X-Y plane within different range of z intervals in cylindrical coordinates.

Line 188. The polar ring current seems to be stronger at the nightside when referring to blue colors near the grey circle in Figure 4b.

Thank you for this helpful comment. We re-examined the polar ring current density distribution. When using the less restrictive error criterion of $|\nabla\cdot B|/|\nabla\times B| < 2$ as shown in Figure B5c, the current density on the dayside is indeed larger than that on the nightside.

However, the error criterion adopted in the main analysis of this study is more stringent, namely $|\nabla\cdot B|/|\nabla\times B| < 2$. Under this criterion, we do not consider the day-night difference in the polar ring current density to be sufficiently robust for a quantitative comparison. To avoid overinterpretation and ensure a more rigorous presentation, we have therefore removed the comparison between the dayside and nightside polar current densities.

In the revised manuscript, we now only describe the overall range of the polar ring current density.

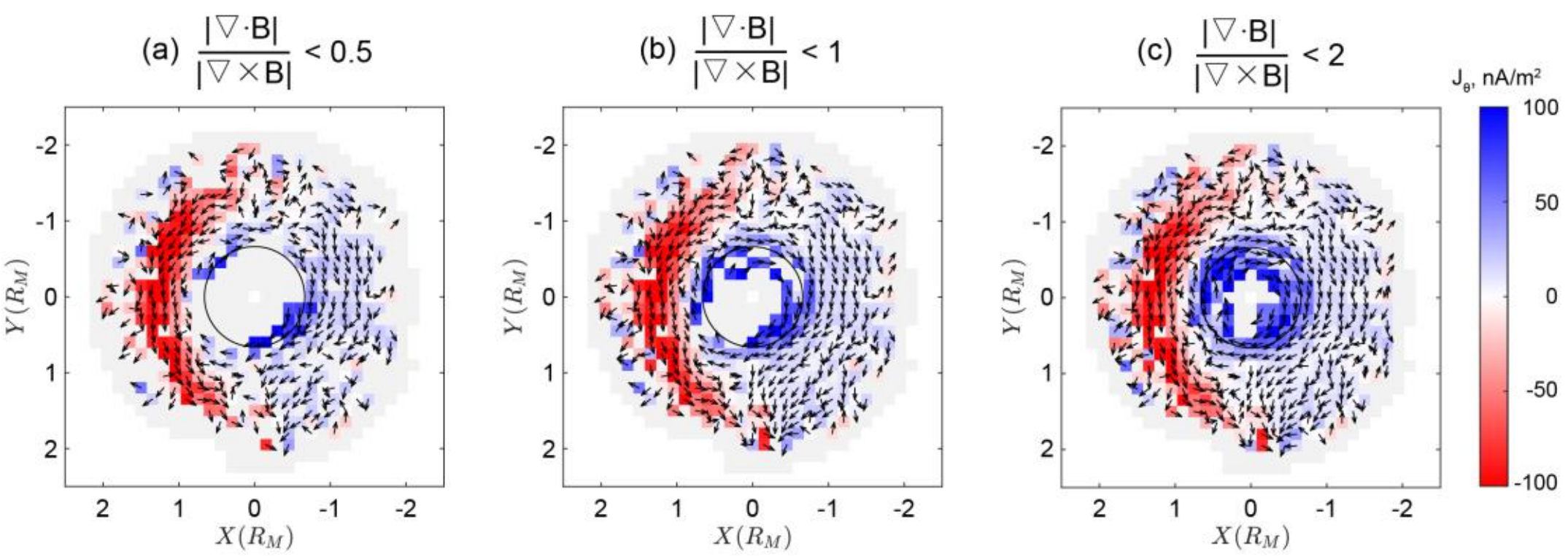

Figure B5 / Extended Data Fig. 4. Current density of the $\boldsymbol{J}_{\text{polar}}$ in different error filtering criterion.

Line 213. latitude -> altitude

We checked the descriptions and it was correct. And “the dawn-to-dusk current at low latitude” is compared to “the dawn-to-dusk current at high latitude, i.e. $\boldsymbol{J}_{\text{polar}}$” in the manuscript.

Line 258. reversal -> reversed

Corrected.

Line 274. Each column of panels is colored by the components ... coordinates, respectively. -> Panel b is colored by the azimuthal component ... coordinates.

Thanks for the reminding. The description of Figure 5b was corrected in the revised manuscript.

Reply to Prof. Stepanova / Reviewer #3:

The authors have successfully addressed the reviewers' suggestions, significantly strengthening the manuscript. The results obtained clearly warrant publication in Nature Communications. However, the manuscript is still challenging to follow in places. I therefore recommend a full editorial revision focused on enhancing the overall style, clarity, and fluency. While I might not typically raise this issue for publication in a specialized journal, Nature Communications is aimed at a broader audience. Therefore, the manuscript needs to be exceptionally clear to ensure a strong scientific impact on the wider scientific community. Next, I will present two paragraphs solely to illustrate my point.

lines 34-39

Mercury has an intrinsic dipole magnetic field with a northward offset (1, 2, 3). Mercury’s intrinsic magnetic field interacts with the solar wind and forms a magnetosphere, which was discovered by Mariner-10 in the 1970s (4) and has been the target of the NASA space mission MESSENGER (5) and of the upcoming ESA-JAXA BepiColombo mission (6). The Mercury’s magnetosphere is highly compressed on the dayside by the conducting solar wind due to Mercury’s proximity to the Sun. ---->

Mercury has an intrinsic dipole magnetic field characterized by a northward offset (1, 2, 3). Its interaction with the solar wind forms a magnetosphere, first discovered by Mariner 10 (4) and subsequently studied by NASA’s MESSENGER (5) and the ESA-JAXA BepiColombo missions (6).

Due to the planet's proximity to the Sun, Mercury’s magnetosphere is highly compressed on the dayside by the dense solar wind.

lines 58-62

The magnetospheric model of Mercury’s magnetosphere by Pump et al. (15) further verified the existence of the eastward ring current. The westward ring current is validated by test-particle simulation (9) and the eastward current is well reproduced by hybrid simulations (10) and the magnetospheric model (15). ---->

While the westward ring current has been validated by test-particle simulations (9), the eastward current is well supported by hybrid simulations (10) and the magnetospheric model of Pump et al. (15).

We thank you for the positive assessment of our work and for the constructive suggestion to improve

the clarity and readability of the manuscript. We agree that, for the broad readership of *Nature Communications*, the manuscript should present the scientific motivation, results, and implications in a clear and accessible way.

In response, we have carried out a thorough editorial revision of the manuscript, with particular attention to the logical flow and fluency.

In the Introduction, we reorganized the narrative to guide readers from Mercury's basic magnetospheric properties, to the importance of electric currents, to the reasons why Mercury's current systems cannot be treated simply as scaled-down terrestrial analogues. We also restructured the review of previous work by grouping reported currents according to current type, and more clearly stated the knowledge gap addressed by this study.

We also revised the Results and subsequent sections to make the presentation easier to follow. We separated observational findings from methodological limitations and robustness tests, particularly for the inner equatorial ring current and the polar ring current.

Throughout the manuscript, we further revised wording, simplified long sentences, reduced ambiguity, and improved transitions between paragraphs. We believe these changes substantially improve the style, clarity, and fluency of the manuscript and make the main findings more accessible to the broader scientific audience of *Nature Communications*.
